# Inhibition of RNA polymerase II-activating CDK9 and CDK12/13, but not of cell cycle relevant CDKs, induces apoptosis by downregulating the short-lived Bcl-2 proteins Mcl1 and Bfl1/A1

**DOI:** 10.1038/s41419-026-08889-6

**Published:** 2026-05-27

**Authors:** Karina S. Krings, Judith Hatzfeld, Sandra Weller, Nadine Borkowski, Tanya R. Llewellyn, Laura Schmitt, Bo Scherer, Harri M. Itkonen, Christoph Peter, Björn Stork, Stefanie Geyh, Jan Eickhoff, Bert Klebl, Nan Qin, Frank Essmann, Sebastian Wesselborg

**Affiliations:** 1 Institute for Molecular Medicine I, Medical Faculty and University Hospital Düsseldorf, Heinrich Heine University Düsseldorf, Düsseldorf, Germany; 2 Robert Bosch Center for Tumor Diseases Stuttgart, Stuttgart, Germany; 3 Institute of Hematology, Oncology and Clinical Immunology, Medical Faculty and University Hospital Düsseldorf, Heinrich Heine University Düsseldorf, Düsseldorf, Germany; 4 Department of Biochemistry and Developmental Biology, Faculty of Medicine, University of Helsinki, Helsinki, Finland; 5 Department of Clinical Molecular Biology, EpiGen, Institute of Clinical Medicine, University of Oslo, Oslo, Norway; 6 EpiGen, Medical Division, Akershus University Hospital, Lørenskog, Norway; 7 Lead Discovery Center GmbH, Dortmund, Germany; 8 Center for Integrated Oncology Aachen-Bonn-Cologne-Düsseldorf (CIO ABCD), Düsseldorf, Germany

**Keywords:** Apoptosis, Targeted therapies, Kinases, Drug development

## Abstract

Cyclin-dependent kinases (CDKs) play a crucial role in cell cycle (such as CDK1 and CDK4/6) and transcription (such as CDK7, CDK9, and CDK12/13). While CDK inhibitors are clinically effective in cancer therapy, their mechanisms of apoptosis induction remain incompletely understood. Here, we demonstrate that inhibition of CDKs involved in cell cycle control (such as CDK1 or CDK4/6) displays no cytotoxic potential. Surprisingly, inhibition of CDK7, which is involved in the control of cell cycle and transcriptional initiation, also showed no cytotoxicity. Only inhibition of CDK9 (by AZD4573 and atuveciclib) or of CDK12/13 (by SR4835 and THZ531)–which target the transcriptional elongation of RNA polymerase II (RNAPII)–exerted a strong apoptotic potential. Since CDK9 and CDK12/13 target different elongation factors associated with RNAPII (such as SPT6 and NELF for CDK9; CDC73, and LEO1 for CDK12/13), they cannot substitute for each other. Consequently, the combination of AZD4573 with SR4835 resulted in significant, synergistic cytotoxicity. Inhibiting CDK9 or CDK12/13 induced the rapid downregulation of the short-lived, anti-apoptotic Bcl-2 proteins Mcl1 and A1/Bfl1 in Jurkat leukemia cells and SUDHL1 lymphoma cells, whereas the expression of Bcl-2 and Bcl-xL remained unaffected. Since Mcl1 and A1 antagonize the pro-apoptotic Bcl-2 proteins Bak and Bax, apoptosis induction by AZD4573 or SR4835 was blocked in Bak- and Bax/Bak-deficient Jurkat cells and strongly reduced in Bax-deficient cells. Because Bcl-2 only inhibits Bax, but not Bak, AZD4573 and SR4835 were able to induce apoptosis in Jurkat cells overexpressing Bcl-2. As tumor cells frequently upregulate Bcl-2, inhibitors of CDK9 and CDK12/13 represent promising anticancer drugs.

## introduction

**Fig. 1:**
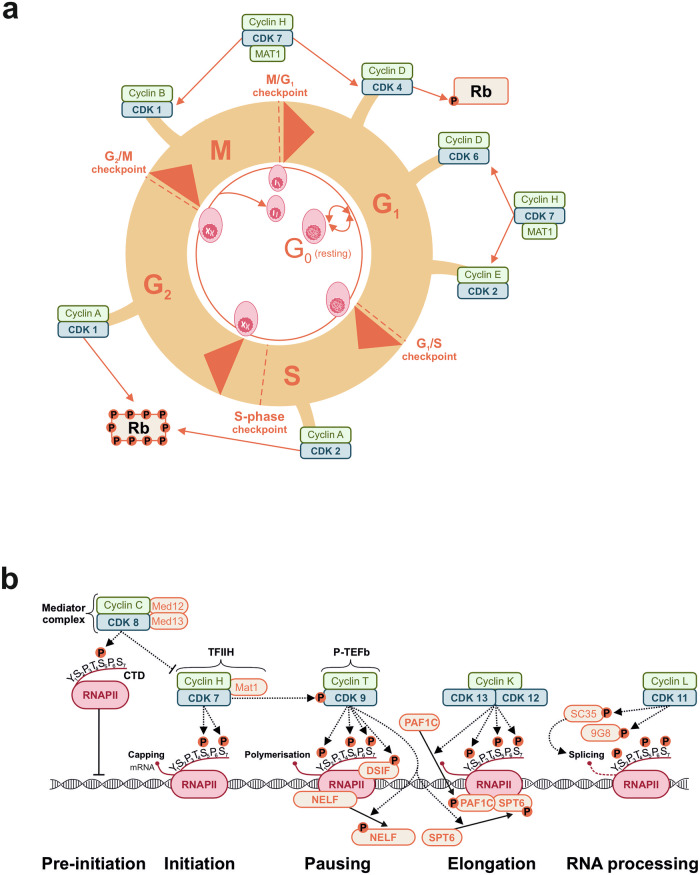
Schematic representation of cyclin-dependent kinases (CDKs) involved in cell cycle regulation and transcription. **a** Depicted are the different CDKs and their associated cyclins, that regulate the progression of the cell cycle, including the key CDKs in G_1_- (CDK4, 6 and 2), S- (CDK2), G_2_- (CDK1) and M-Phase (CDK1) and CDK7, which influences all phases. Adapted from [4]. **b** Shown are the key transcriptional CDKs with their respective cyclin partner and their function in regulating transcriptional pre-initiation, initiation, pausing, elongation, and RNA-processing (termination). The Mediator complex (CDK8/cyclin C/Med12/13) is active before the initiation and negatively regulates the start of transcription. The TFIIH complex with CDK7/cyclin H/Mat1 is involved in transcription initiation, while CDK9/cyclin T (P-TEFb) and CDK12/13/cyclin K regulate transcription pausing and elongation. CDK9 and CDK12/13 phosphorylate different transcription elongation factors and the RNAPII itself. CDK11/cyclin L with the help of cyclin L promote mRNA splicing. Adapted from [1]. Rb retinoblastoma protein, RNAPII RNA polymerase II.

Cyclin-dependent kinases (CDKs) are serine/threonine protein kinases that play a fundamental role in the regulation of cell cycle and transcription (Fig. 1). As their denomination indicates, CDKs require binding to their respective cyclins for activation. They can be divided into two subfamilies: (i) CDKs involved in cell cycle control (e.g., CDK1, 2, 4, 6, and 7), and (ii) CDKs controlling transcription (e.g., CDK7, 8, 9, 11, 12, and 13) [1, 2].

The cell cycle consists of four phases: G_1_ phase, S phase, G_2_ phase, and M phase (mitosis and cytokinesis). CDK1, CDK2, CDK4, CDK6, and CDK7 function as regulators of cell cycle progression and cell division through the control of various transcription factors and regulatory elements such as the retinoblastoma protein (Rb). During the initial G_1_ phase, cyclin D levels increase, which activates CDK4 and CDK6 and subsequently promotes the phosphorylation of Rb. Rb phosphorylation results in the activation of transcription factors of the E2F family, thereby increasing the transcription of E2F-responsive genes that are necessary for cell cycle progression [3, 4]. CDK2/cyclin E mediate the transition from G_1_ to S phase. Similarly, cyclin A, in conjunction with CDK2 or CDK1, facilitates progression of the S phase, the entry into G_2_ phase, and the transition to M phase by phosphorylating specific sets of substrates. As a component of the CDK-activating kinase (CAK) complex, CDK7 catalyzes the phosphorylation of CDK1, CDK2, CDK4, and CDK6, thereby activating their kinase activity. Consequently, CDK7 exerts a regulatory influence over all phases of the cell cycle [3, 4] (Fig. 1a).

Similarly, the coordinated activity of transcriptional CDKs (CDK7, CDK8, CDK9, CDK11, CDK12, and CDK13) is required for RNA polymerase II (RNAPII) mediated transcription. This process comprises five stages: pre-initiation, initiation, pausing, elongation, and termination [5] (Fig. 1b). The C-terminal domain (CTD) of RNAPII’s largest subunit (RFB-1) contains 52 repeats of the heptapeptide Tyr1-Ser2-Pro3-Thr4-Ser5-Pro6-Ser7, which serves as a flexible binding scaffold for numerous nuclear factors. This heptapeptide is phosphorylated at different sites by CDK7, CDK9, and CDK12/13. Thus, studies have described the phosphorylation of Ser5 and Ser7 by CDK7, as well as the phosphorylation of Ser2, Ser5, and Ser7 by CDK9 and CDK12/13 [1, 5–7].

During the pre-initiation phase, CDK8 within the Mediator complex functions as a negative regulator of transcription by phosphorylating the CTD of RNAPII. As a consequence, RNAPII is no longer hypophosphorylated, which disables its DNA binding. Additionally, the Mediator complex inhibits the transcription initiation factor IIH (TFIIH), which includes CDK7, cyclin H, and Mat1. Beyond its function in cell cycle regulation, CDK7, as part of the TFIIH complex, promotes the phosphorylation of Ser5 and Ser7 within the CTD of RNAPII. This contributes to the escape of RNAPII from the promoter region and the induction of RNA capping. In addition to the CTD of RNAPII, CDK7 can also phosphorylate and activate the transcription-associated kinases CDK9, CDK12, and CDK13 [1, 5].

Upon promoter escape, transcription is provisionally paused 20–60 bp downstream of the transcription start site through the association of two negative factors, DRB sensitivity-inducing factor (DSIF) and negative elongation factor (NELF), which is mediated by CDK7 [1, 5, 6, 8]. DSIF consists of the two proteins, SPT4 and SPT5. In its unphosphorylated state, DSIF promotes RNAPII pausing [9], whereas phosphorylation by CDK9/cyclin T (P-TEFb) turns SPT5 from a repressor into an activator of transcription [1, 5, 10]. In addition to SPT5, CDK9 also phosphorylates the elongation factor SPT6 and NELF. This leads to the recruitment of SPT6 to RNAPII and the release of the roadblock NELF. The release of NELF enables the binding of PAF1C, since both complexes share a common binding interface on RNAPII [11]. PAF1C consists of the subunits PAF1, LEO1, CDC73, CTR9, WDR61 and Rtf1. CDK12/13/cyclin K phosphorylate LEO1, thereby stabilizing the association of PAF1C with RNAPII and promoting productive elongation [7, 8, 12, 13] (Fig. 1b). Consequently, CDK9, CDK12, and CDK13 play a central role in regulating RNAPII entry into productive elongation, and thereby ensure efficient mRNA synthesis [1, 5]. Upon completion of transcription, CDK11/cyclin L is involved in RNA processing by phosphorylating factors responsible for pre-mRNA splicing, including SC35 and 9G8 (Fig. 1b).

CDKs are frequently overexpressed in various tumors, thus rendering CDK inhibitors (CDKi) suitable candidates for targeted therapy [1, 2, 4, 5, 14, 15]. While most clinically approved CDKis target cell cycle-related kinases, transcriptional CDKis are becoming an increasingly important focus of clinical development [14]. To date, only CDKis targeting cell cycle control (such as the CDK4/6 inhibitors palbociclib, ribociclib, and abemaciclib) have been approved for anticancer therapy by the FDA. Additionally, several CDK4/6 inhibitors are under investigation, such as lerociclib, trilaciclib, and SHR6390 [2]. So far, there are no FDA approved transcriptional CDK inhibitors–though several inhibitors are in preclinical and clinical studies, such as LDC4297 (CDK7), QS1189 (CDK7), BS181 (CDK7), and ICEC0942 (CDK7), THZ1 (CDK7, CDK12/13), THZ2, (CDK7), YKL-5-124 (CDK7), SY-5609 (CDK7), AZD4573 (CDK9), THZ531 (CDK12/13), and cortistatin A (CDK8/19) [1, 2, 5, 14, 16–18].

Nevertheless, the use of CDK9 inhibitors has led to the partial elucidation of the mechanism of action of apoptosis induction in cancer cells through transcriptional CDK inhibitors. The CDK9 inhibitor AZD4573 has been shown to disrupt transcriptional activity, particularly the expression of the two short-lived Bcl-2 proteins, Mcl1 (BCL2L3) and A1 (BCL2A1, Bfl1), which inhibit apoptosis induction via Bax and Bak. Consequently, downregulation of Mcl1 and A1 by CDK9 inhibition results in apoptosis [19–21].

However, in addition to inhibiting transcriptional CDKs, numerous reports state that CDK inhibitors targeting cell cycle control can also induce apoptosis [1, 15, 22–32]. We therefore performed a comprehensive study to investigate the apoptotic mechanisms of CDK inhibitors, with particular focus on the potential involvement of cell cycle and transcriptional control. In addition to CDK9 inhibitors, our study also focused on CDK12/13 inhibitors, which have not been thoroughly examined concerning their apoptotic signaling to date [17, 33–35]. CDK12/13 inhibitors target both CDK12 and its paralog CDK13 simultaneously, as they are highly similar in structure and function, and they share approximately 92% sequence identity within their kinase domains [18]. As many of the investigated CDKs are vital in dividing cells, we used highly specific CDK inhibitors to evaluate their role in apoptosis signaling.

## Results

### Inhibiting transcriptional elongation (CDK9 and CDK12/13), rather than transcriptional initiation (CDK7) or cell cycle-controlling CDKs (CDK1 and CDK4/6), induces cytotoxicity and apoptosis

**Fig. 2:**
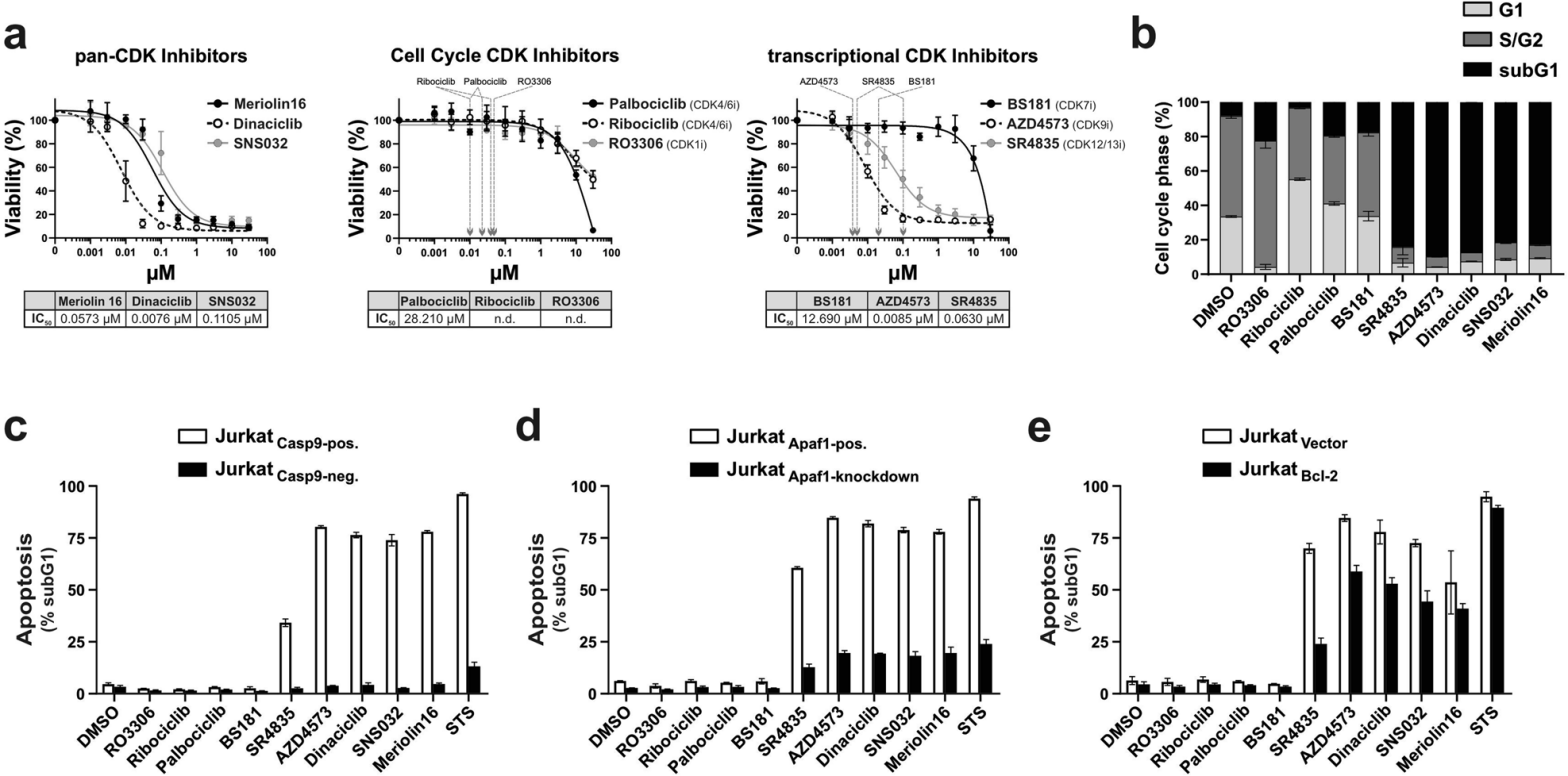
Inhibiting transcriptional elongation (CDK9 and CDK12/13), but not transcriptional initiation (CDK7) or cell cycle-controlling CDKs (CDK1 and CDK4/6), activates the mitochondrial apoptosis pathway, even in the presence of Bcl-2. **a** Jurkat cells were treated with increasing concentrations of pan-CDK, cell cycle-specific, or transcriptional CDK inhibitors. Cell viability was assessed by AlamarBlue^®^ assay after 24 h. The cytotoxicity IC_50_ values are shown below each graph. The enzymatic IC_50_ values of CDK specific inhibitors (obtained from MedChemExpress (https://www.medchemexpress.com); see Table 1) are indicated by gray arrows. Error bars represent the mean ± SD of three independent experiments, performed in triplicate. **b** For cell cycle analysis, Jurkat cells were treated with the indicated inhibitors at a concentration of 10 µM or DMSO (0.1% v/v; diluent control) for 24 h. Detection of cell cycle phases was performed by flow-cytometric analysis of the DNA content of propidium iodide-stained nuclei using the Nicoletti assay [86]. Shown are cells in G_1_ phase, S/G_2_ phase, and hypodiploid apoptotic nuclei (subG1). **c**, **d** CDK inhibitor-induced apoptosis requires caspase-9 and Apaf-1. **c** Caspase-9 proficient (Jurkat Casp9-pos., white bars) or Caspase-9 deficient Jurkat cells (Jurkat Casp9-neg., black bars) and **d** Apaf-1 proficient (Jurkat Apaf1-pos.; white bars) or Apaf-1 knockdown Jurkat cells (Jurkat Apaf1-knockdown; black bars) were treated with 0.1% (v/v) DMSO, with 1 µM of the indicated CDK inhibitors or 2.5 µM staurosporine (STS; as a positive control for apoptosis induction). After 24 h of incubation, apoptosis-related DNA fragmentation was detected via flow-cytometric measurement of propidium iodide stained apoptotic hypodiploid nuclei. **e** CDK inhibitors induce apoptosis in the presence of antiapoptotic Bcl-2. Jurkat cells stably transfected with vectors encoding Bcl-2 (Jurkat Bcl-2; black bars) or empty vector (Jurkat vector; white bars) were treated as in (**c**, **d**). After 24 h, apoptosis was assessed by flow cytometric measurement of apoptotic hypodiploid nuclei. **b–e** Error bars represent the mean ± SD, performed in triplicate.

Besides transcriptional CDK inhibitors, numerous reports claim that CDK inhibitors targeting cell cycle control can also induce apoptosis [1, 15, 22–32]. To investigate the extent to which CDK inhibitors involved in cell cycle or transcription control can induce apoptosis, we used a broad panel of CDK inhibitors (CDKis) with highly specific profiles for their respective target CDKs (see Table 1). To inhibit cell cycle-controlling CDKs, we applied RO3306 (a CDK1 inhibitor) and the two FDA-approved CDK4/6 inhibitors palbociclib and ribociclib [2]. To target transcriptional CDKs, we used the following inhibitors: BS181, LDC4297, and THZ1 (CDK7 inhibitors); AZD4573 and atuveciclib (CDK9 inhibitors); and SR4835 and THZ531 (CDK12/13 inhibitors). As positive control, we used the pan-CDK inhibitors dinaciclib (targeting CDK1, 2, 5, and 9), SNS032 (targeting CDK1, 2, 4, 7, and 9), and meriolin16, which has recently been shown to target CDK1, 2, 7, 9, 12, and 13 [36]. As shown in Fig. 2a, inhibition of cell cycle-controlling CDKs by RO3306 (CDK1i), palbociclib, or ribociclib (CDK4/6i) did not induce any cytotoxicity in Jurkat leukemia cells after 24 h at concentrations that specifically inhibit their respective kinase activity (see Table 1). Since cytotoxicity induced by cell cycle inhibition might require a prolonged exposure to the respective CDK inhibitors, we treated Jurkat cells for 72 h with RO3306 (CDK1i), palbociclib, or ribociclib (CDK4/6i). However, as shown in Supplementary Fig. S1, we observed no increase in cytotoxicity or apoptosis induction at 72 h compared to 24 h, at concentrations that specifically inhibit their respective kinase activity. However, cell cycle progression was impaired, as indicated by an altered phase distribution (Fig. 2b). Surprisingly, the inhibition of CDK7 (by BS181 or LDC4297), which is involved in the control of cell cycle as well as transcriptional initiation, also displayed no cytotoxic potential at concentrations at which the respective inhibitors specifically target CDK7 kinase activity (Fig. 2a and Supplementary Fig. S2a–c). Even after 72 h, BS181 did not induce any cytotoxicity or apoptosis (Supplementary Fig. S1d, h). As shown in Supplementary Fig. S2a–c, THZ1 induced cytotoxicity and apoptosis. However, it should be noted that THZ1 has a higher specificity for CDK12 (enzymatic IC_50_: 33 nM) and CDK13 (IC_50_: 19 nM) than for CDK7 (IC_50_: 110 nM) (see Table 1). Only targeting transcriptional elongation with CDK9 inhibitors (AZD4573 or atuveciclib), or CDK12/13 inhibitors (SR4835 or THZ531) induced pronounced cytotoxicity and apoptosis (Fig. 2a and Supplementary Fig. S2). Similarly, all three pan-CDK inhibitors (meriolin16, dinaciclib, and SNS032) induced cytotoxicity at concentrations in the nanomolar range (Fig. 2a).

**Table 1:** List of applied CDK inhibitors and their respective IC_50_ values of enzymatic activity.

	CDK1	CDK2	CDK4	CDK6	CDK7	CDK8	CDK9	CDK11	CDK12	CDK13
**pan-CDK inhibitors**										
**Dinaciclib**	3 nM^a^ 26 nM^e^	1 nM^a^ 78 nM^e^	37 nM^e^	270 nM^e^	150 nM^e^	37 nM^e^	4 nM^a^ 32 nM^e^	7.9 µM ^h^	34 nM^e^	47 nM^e^
**SNS032**	480 nM^a^ 130 nM^e^	38 nM^a^ 11 nM^e^	925 nM^a^ 350 nM^e^	2.9 µM^e^	62 nM^a^ 100 nM^e^	14 nM^e^	4 nM^a^ 5 nM^e^		<0.05 nM^e^	<0.05 nM^e^
**CDK1 inhibitor**										
**RO3306**	**35** **nM**^a^ **47** **nM**^b^ **203** **nM**^e^	340 nM^a^ 186/253 nM^b^ 490 nM^e^	>20 µM^b^ >10 µM^e^	>10 µM^e^	17.4 µM^b^ >10 µM^e^		14.5 µM^b^ >10 µM^e^		>10 µM^e^	>10 µM^e^
**CDK4/6 inhibitors**										
**Palbociclib**	9.8 µM^b^ >10 µM^e^	9.1/1.8 µM^b^ 8 µM^e^	**11** **nM**^a^ **13** **nM**^b^ **9** **nM**^e^	**16** **nM**^a^ **23** **nM**^e^	>10 µM^b^ >10 µM^e^	>10 µM^e^	1.1 µM^b^		1.3 µM^e^	1.63 µM^e^
**Ribociclib**	>20 µM^b^	>20 µM^b^	**10** **nM**^a^ **30** **nM**^b^	**39** **nM**^a^	>20 µM^b^		3.9 µM^b^		0.95 µM^e^	>10 µM^e^
**CDK7 inhibitors**										
**BS181**	8.1 µM^a,c^ >10 µM^d^	880 nM^a,c^ 4.58 µM ^d^	33 µM^a,c^ >10 µM^d^	47 µM^a,c^ >10 µM^d^	**21** **nM**^a,c^ **57.1 nM**^d^		4.2 µM^a,c^ 1.94 µM^d^			
**LDC4297** (LDC044297)	53.7 nM^d^	6.4 nM^d^	>10 µM^d^	>10 µM^d^	**0.13** **nM**^a^ <**5** **nM** ^d^ **6.67** **nM**^e^	>10 µM^e^	1.71 µM^d^ 2.9 µM^e^		71 nM^e^	209 nM^e^
**THZ1** (LDC198304)	0.89 µM^e^	3.6 µM^e^	>10 µM^e^	>10 µM^e^	**3.2nM**^a^ **110** **nM**^e^		1.78 µM^e^		19 nM^e^	33 nM^e^
**CDK9 inhibitors**										
**AZD4573**	117 nM^f^	52 nM^f^	499 nM^f^	363 nM^f^	1.37 µM^f^		**<4** **nM**^a^ <**4 nM**^f^		8.07 µM^f^	
**Atuveciclib** (LDC208447) (BAY1143572)	1100 nM^g^	1000 nM^g^		>10 µM^g^	>10 µM^e^ >10 µM^g^	>10 µM^e^	**13** **nM**^a^ **6 nM**^g^		468 nM^e^	2.12 µM^e^
**CDK12/13 inhibitors**										
**SR4835**		>10 µM^e^			>10 µM^e^				**99** **nM**^a^ **450** **nM**^e^	**4.9** **nM**^a^
**THZ531** (LDC208434)	4 µM^e^	610 nM^e^	6.6 µM^e^	9.5 µM^e^	894 nM^e^		3.22 µM^e^		**158** **nM**^a^ **15.9** **nM**^e^	**69** **nM**^a^ **34.3** **nM**^e^

^a^https://www.medchemexpress.com

^b^Jorda et al. (2018) J Med Chem [87].

^c^Ali et al. (2009) Cancer Res [39].

^d^Kelso et al. (2014) Mol Cell Biol [49].

^e^Values were determined at LDC (Dr. Bert Klebl & Dr. Jan Eickhoff; Lead Discovery Center GmbH, Dortmund, Germany).

^f^Barlaam et al. (2020) J Med Chem [19].

^g^Lücking et al. (2017) ChemMedChem [88].

### CDK9 and CDK12/13 inhibitors activate the mitochondrial apoptosis pathway, even in the presence of anti-apoptotic Bcl-2

Concerning the apoptosis signaling pathway affected by CDK inhibition, we could recently show that apoptosis triggered by different meriolin derivatives (meriolin16, 31, 36) was mediated by the intrinsic mitochondrial death pathway, as it was blocked in caspase-9 deficient and Apaf-1 knockdown Jurkat cells [37]. However, all meriolin derivatives were able to induce apoptosis in Jurkat cells overexpressing the anti-apoptotic protein Bcl-2. In the same way, apoptosis induction by other pan-CDK inhibitors such as dinaciclib and SNS032 and more selective inhibition of CDK9 via AZD4573 and CDK12/13 by SR4835 was blocked in caspase-9 deficient and Apaf-1 knockdown Jurkat cells, and only partially reduced in Bcl-2 overexpressing Jurkat cells (Fig. 2c–e and Supplementary Fig. S3). Thus, both AZD4573 and SR4835 are capable of activating the mitochondrial apoptosis pathway in the presence of Bcl-2.

### Inhibition of CDK9 and CDK12/13, but not of CDK7, targets RNAPII phosphorylation and downregulates the anti-apoptotic protein Mcl1

**Fig. 3:**
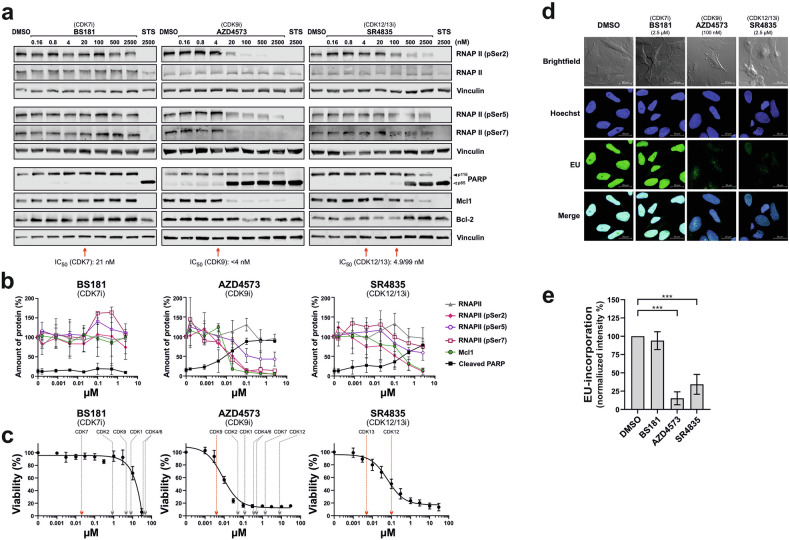
Inhibiting CDK9 and CDK12/13, but not CDK7, targets the phosphorylation of the C-terminal domain of RNAPII and decreases the expression of the anti-apoptotic protein Mcl1. **a** Jurkat cells were treated with increasing concentrations of the CDK7 inhibitor BS181, the CDK9 inhibitor AZD-4573, or the CDK12/13 inhibitor SR-4835, for 6 h and then immunoblotted for: RNA polymerase II (RNAPII), phospho-Ser2 of the CTD of RNAPII (RNAPII pSer2), RNAPII Ser5, RNAPII pSer7, Mcl1, Bcl-2, vinculin (served as loading control), and cleavage of the caspase substrate PARP (solid arrowheads indicate the uncleaved form of PARP (p116); open arrowheads indicate the cleaved form (p85)). The enzymatic IC_50_ values of CDK specific inhibitors (obtained from MedChemExpress (https://www.medchemexpress.com); see Table 1) are indicated by red and gray arrows, respectively. One representative immunoblot from three independent experiments is shown. **b** Quantification of the immunoblot kinetics from (**a**). Protein expression of RNAPII, RNAPII (pSer2, pSer5, pSer7), and Mcl1 was normalized to vinculin. Cleaved PARP (p85) was normalized to the total amount of PARP (p85 + p116). **c** Viability assays of Jurkat cells that have been treated with increasing concentrations of the CDK7 inhibitor BS181, the CDK9 inhibitor AZD4573, or the CDK12/13 inhibitor SR4835 for 24 h. The viability curves depicted in Fig. 2a are shown here in separate graphs, to illustrate the enzymatic IC₅₀ values of all CDKs. Arrows indicate the enzymatic IC_50_ values of targeted CDKs by respective CDK inhibitors (IC_50_ values of BS181 and SR4835 were obtained from MedChemExpress (https://www.medchemexpress.com), and the IC_50_ value of AZD4573 from Barlaam et al. [19]; see Table 1). Error bars represent the mean ± SD of three independent experiments, performed in triplicate. **d** Measurement of transcriptional activity by EU incorporation. HeLa cells were treated with 2.5 µM of the CDK7 inhibitor BS181, 100 nM of the CDK9 inhibitor AZD4573, 2.5 µM of the CDK12/13 inhibitor SR4835, or 0.1% DMSO for 24 h. EU-incorporation was determined by microscopy, and exemplary images are shown (green: EU-incorporation; blue: DAPI stained nuclei). **e** Quantification of EU fluorescence intensity from three independent experiments (two technical replicates, five images per replicate, ≥1670 cells were analyzed per condition). Statistical significance: *p < 0.05, **p < 0.01, ***p < 0.001.

To investigate the role of transcriptional CDKs in the phosphorylation of the C-terminal domain (CTD) of RNAPII, we performed titrations with different CDK7is (BS181, LDC4297, THZ1), the CDK9i AZD4573, and the CDK12/13i SR4835 in Jurkat cells. We then investigated the inhibition of Ser2, Ser5, and Ser7 phosphorylation within RNAPII’s CTD using immunoblotting. In addition, we monitored the expression of the anti-apoptotic Bcl-2 proteins Mcl1 (BCL2L3) and Bcl-2, as previous reports have shown that CDK9i downregulates the protein expression of short-lived Mcl1 [19, 20, 38–40]. As a surrogate marker for caspase activation, the cleavage of the caspase substrate poly(ADP-ribose) polymerase-1 (PARP) was monitored. As shown in Fig. 3a–c and Supplementary Fig. S4, the CDK7 inhibitors BS181 and LDC4297 did not induce dephosphorylation of pSer2, pSer5, or pSer7 of the CTD of RNAPII, downregulation of Mcl1, or cytotoxicity in Jurkat leukemia cells at concentrations that specifically inhibited the enzymatic activity of CDK7. Similarly, no effect of BS181 could be observed in other leukemia cell lines, such as HL60 (AML), KOPTK1 (T-ALL), or SUPB15 (B-ALL) (Supplementary Fig. S5). As mentioned above, the alleged CDK7 inhibitor THZ1 also targets CDK12 and CDK13 at nanomolar concentrations. This complicates determining its specific effect on CDK7 (see Table 1, Supplementary Fig. S2a–c and S4). Only inhibition of CDK9 via AZD4573 or CDK12/13 by SR4835 induced a pronounced dephosphorylation of pSer2, pSer5, or pSer7 of the CTD of RNAPII, downregulation of Mcl1, cleavage of the caspase-substrate PARP after 6 h, and cytotoxicity after 24 h treatment (Fig. 3a–c). However, the expression of Bcl-2 was not affected within 6 h of treatment with AZD4573 or SR4835 (Fig. 3a). Consequently, the concentrations selected for subsequent experiments were based on these data. In subsequent experiments, we used 100 nM for AZD4573 (CDK9i) and 2.5 µM for SR4835 (CDK12/13i), as these concentrations induced pronounced toxicity and RNAPII dephosphorylation, while ensuring inhibitor specificity.

It was quite unexpected that inhibition of CDK7 via BS181 or LDC4297 did not block the phosphorylation of the CTD of RNAPII and had no effect on apoptosis induction. Since CDK7 plays a crucial role in transcriptional initiation, we measured the effect of inhibition of CDK7, CDK9, and CDK12/13 on the de novo RNA-synthesis by the incorporation of 5-ethynyl-uridine (EU) in HeLa cells after 24 h. As shown in the immunofluorescence analysis in Fig. 3d, e, EU incorporation was reduced by ~85% through AZD4573 (CDK9i) and by ~65% by SR4835 (CDK12/13i), whereas BS181 (CDK7i) had no effect on RNA-synthesis. To exclude any influence of caspases on RNA synthesis, we performed the same experiment in the presence of the pan-caspase inhibitor Q-VD-OPh (QVD), which yielded virtually the same results (Supplementary Fig. S6). Taken together, unlike inhibition of CDK9 and CDK12/13, it appears that inhibition of CDK7 does not induce CTD-dephosphorylation, nor downregulation of anti-apoptotic Mcl1 and subsequent apoptosis.

### Inhibition of CDK9 and CDK12/13 induces the rapid downregulation of the anti-apoptotic Bcl-2 proteins Mcl1 and A1/Bfl1

**Fig. 4:**
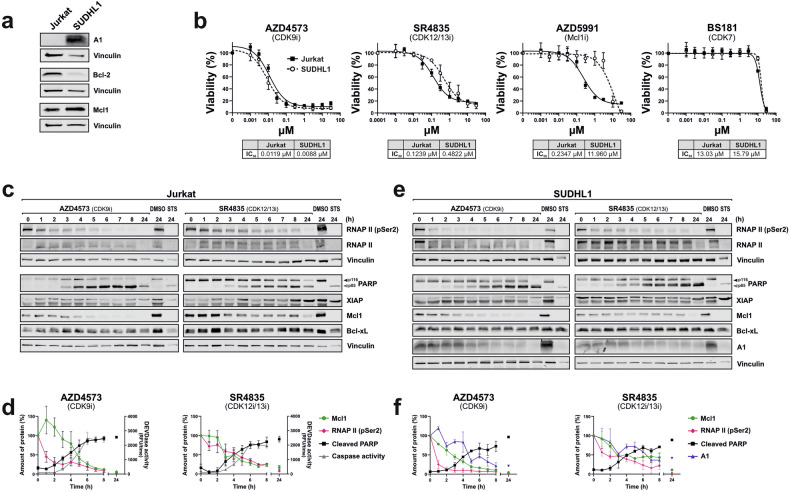
Inhibiting CDK9 or CDK12/13 rapidly downregulates the anti-apoptotic Bcl-2 proteins Mcl1 and A1/Bfl1. **a** Expression of Mcl1, A1, and Bcl-2 in Jurkat leukemia and SUDHL1 lymphoma cells detected by immunoblotting. Vinculin was used as loading control. **b** Cell viability assays were performed on Jurkat and SUDHL1 cells that were treated with AZD4573 (CDK9i), SR4835 (CDK12/13i), the Mcl1 inhibitor AZD5991 for 24 h, or BS181 (CDK7i). The cytotoxicity IC_50_ values are shown below each graph. Error bars represent the mean ± SD of three independent experiments, performed in triplicate. **c**, **e** Monitoring of RNAPII CTD dephosphorylation and protein levels in Jurkat and SUDHL1 cells. **c** Jurkat cells, or **e** SUDHL1 cells were treated with 0.1 µM AZD4573 (CDK9i), or 2.5 µM SR4835 (CDK12/13i) for up to 24 h. DMSO (0.1%) was used as a diluent control, and 2.5 µM staurosporine (2.5 µM; STS) as a positive control for apoptosis induction. Protein expression was monitored by immunoblotting for RNA polymerase II (RNAPII), phospho-Ser2 of the CTD of RNAPII (RNAPII pSer2), XIAP, Mcl1, Bcl-xL, vinculin (loading control), and cleavage of the caspase substrate PARP (solid arrowheads indicate the uncleaved form of PARP (p116); open arrowheads indicate the cleaved form (p85)). One representative immunoblot from three independent experiments is shown. **d**, **f** Quantification of the immunoblot kinetics from (**c**, **e**). Protein expression of Mcl1, A1, RNAPII (pSer2) was normalized to vinculin. Cleaved PARP (p85) was normalized to the total amount of PARP (p85 + p116). In addition, caspase-3 activity was determined in Jurkat cells by fluorometric measurement of the caspase-3 substrate DEVD-AMC, which is shown on the right *y*-axis in (**d**). **d**, **f** Error bars represent the mean ± SD from three independent experiments. Caspase assay experiments were performed in triplicate.

In previous studies, Cidado et al. could demonstrate that inhibition of CDK9 by AZD4573 induces the rapid downregulation of Mcl1 on mRNA and protein level within 2 - 4 h in the AML cell line MV411, whereas the expression of Bcl-2 and Bcl-xL was not affected within 9 h treatment [20]. In subsequent studies, they could identify another short-lived anti-apoptotic Bcl-2 protein, namely A1 (BCL2A1, Bfl1), that was downregulated upon AZD4573 treatment [21]. Therefore, we investigated whether inhibition of CDK12/13 would also suppress the expression of A1. Since Jurkat cells express no A1 (Fig. 4a), we used the anaplastic large cell lymphoma (ALCL) cell line SUDHL1, which has been described to express A1 [21]. As shown in Fig. 4a, SUDHL1 cells are A1-positive and express Mcl1 to a similar extent as Jurkat cells. However, unlike Jurkat cells, SUDHL1 cells express almost no Bcl-2. Application of AZD4573 (CDK9i) or SR4835 (CDK12/13i) induced to a similar extent cytotoxicity in Jurkat and SUDHL1 cells (Fig. 4b). However, SUDHL1 cells were much more resistant to treatment with the Mcl1-inhibitor AZD5991 (a BH3-mimetic) than Jurkat cells (Jurkat IC_50_: 0.24 µM vs. SUDHL1 IC_50_: 11.96 µM), indicating that A1 might compensate for the anti-apoptotic function of Mcl1 in SUDHL1 cells. As expected, treatment with BS181 did not induce cytotoxicity in either Jurkat or SUDHL1 cells at concentrations that specifically inhibited the enzymatic activity of CDK7 (Fig. 4b).

Next, we treated Jurkat and SUDHL1 cells with AZD4573 (CDK9i) or SR4835 (CDK12/13i) for up to 24 h and monitored the enzymatic caspase activity, cleavage of the caspase substrate PARP, and the expression of the anti-apoptotic proteins XIAP, Bcl-xL, Mcl1, A1, and also RNAPII and RNAPII-pSer2 by immunoblotting. As shown in Fig. 4c–f, the inhibition of CDK9 or CDK12/13 rapidly induced the dephosphorylation of RNAPII (pSer2) in Jurkat and SUDHL1 cells. This was followed by the downregulation of Mcl1 and subsequent caspase activation, as determined by PARP-cleavage and enzymatic DEVDase activity. In addition to Mcl1, AZD4573 and SR4835 also induced the rapid downregulation of A1 in A1-positive SUDHL1 cells. However, Bcl-xL expression was unaffected, and XIAP expression decreased only after 24 h. To exclude the possibility of caspase-mediated proteolytic degradation of Mcl1 and A1, we performed the same experiments in the presence of the pan-caspase inhibitor QVD. This yielded virtually the same results. As expected, caspase-mediated cleavage of PARP was blocked in the presence of QVD (Supplementary Fig. S7). However, treatment with BS181 (CDK7i) had no effect on the dephosphorylation of RNAPII (pSer2), the downregulation of Mcl1 and A1, or PARP-cleavage in Jurkat and SUDHL1 cells (Supplementary Fig. S8).

### CDK9- and CDK12/13-induced downregulation of Mcl1 and A1/Bfl1 is mediated by proteasomal degradation

To prove that the downregulation of the short-lived proteins Mcl1 and A1 is mediated by proteasomal degradation, we treated Jurkat and SUDHL1 cells with the proteasome inhibitor MG132 prior to treatment with AZD4573 or SR4835. Since active caspases can cleave and inactivate the 19S regulatory complex of the proteasome [41], we performed the experiments in the presence of the pan-caspase inhibitor QVD. As shown in Supplementary Fig. S9, the inhibition of the proteasome with MG132 increased the expression of Mcl1 in both Jurkat and SUDHL1 cells, and with a similar increasing trend for A1 in SUDHL1 cells. This suggests that the downregulation of Mcl1 and A1 induced by AZD4573 and SR4835 is mediated by proteasomal degradation.

### Inhibitors of CDK9 and CDK12/13 target different proteins within the elongation complex and act synergistically in cell death induction

**Fig. 5:**
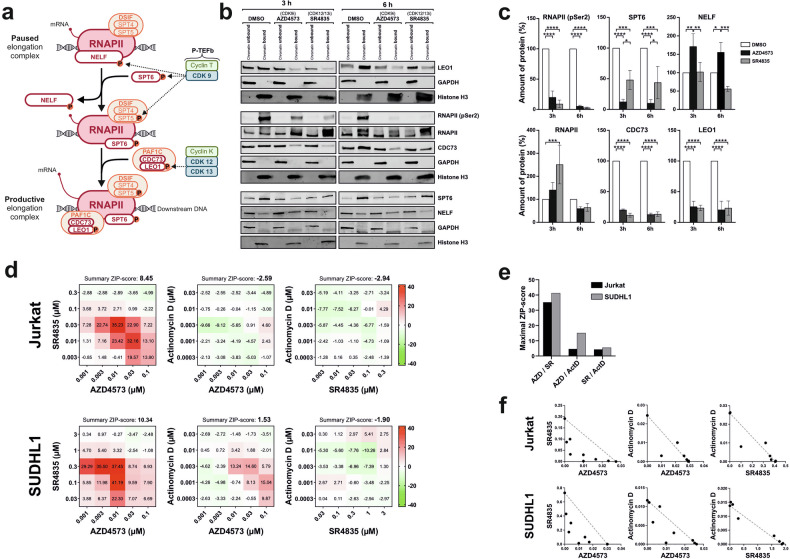
Inhibitors of CDK9 and CDK12/13 target different proteins within the elongation complex and act synergistically in cell death induction. **a** Schematic overview of transcriptional pause release. Productive elongation is mediated by the combined action of CDK9 and CDK12/13. CDK9 phosphorylates SPT5, which turns DSIF from a repressor to an activator of transcription. In addition, CDK9 phosphorylates SPT6 and NELF, which leads to the recruitment of SPT6 and the dissociation of NELF from RNAPII. The release of NELF enables the binding of PAF1C (consisting of PAF1, LEO1, CDC73, CTR9, WDR61, and Rtf1), as both complexes share a common binding interface on RNAPII. CDK12/13 phosphorylate the PAF1C component LEO1, which stabilizes the association of PAF1C with RNAPII and enables productive elongation. **b** Immunoblot analysis of chromatin-bound versus soluble protein fractions after treatment of Jurkat cells with AZD4573 (0.1 µM, CDK9i) or SR4835 (2.5 µM, CDK12/13i) for 3 or 6 h. One representative immunoblot from four independent experiments is shown. **c** Quantification of chromatin-bound proteins from (**b**). Error bars represent the mean ± SD from four independent experiments. Statistical significance: *p < 0.05, **p < 0.01, ***p < 0.001. **d** Jurkat or SUDHL1 cells were treated with AZD4573 (CDK9i) in combination with SR4835 (CDK12/13i). Alternatively, cells were treated with either AZD4573 (CDK9i) or SR4835 (CDK12/13i) in combination with the transcriptional inhibitor actinomycin D. After 24 h, cell viability was assessed by AlamarBlue® assay. Drug concentrations were chosen to cover the full dynamic range of viability responses (from complete survival to complete cell death), to ensure reliable synergy evaluation. Subsequently, SynergyFinder was used to perform a synergy analysis. Combinations of AZD4573 or SR4835 with actinomycin D showed no synergy, whereas cotreatment with AZD4573 and SR4835 yielded a summary ZIP synergy score of ~10, indicating synergistic effects. **e** Maximum ZIP scores from (**d**). Combinations of AZD4573 and SR4835 reached values of 35 and 41, while combinations with actinomycin D remained below 10 (once slightly above 10). **f** The isobologram analysis of the same experimental data shown in (**d**) confirms the strong synergistic effect of AZD4573 and SR4835. **d–f** Results of three independent experiments, performed in duplicate, are shown.

The observation that inhibitors of CDK12/13 display a similar cytotoxic potential as CDK9 inhibitors opens new therapeutic options. However, the question remains: Why can’t they substitute for each other? Both CDK9 and CDK12/13 have been shown to phosphorylate the CTD of RNAPII at Ser2, Ser5, and Ser7 [5], and we also observed the dephosphorylation of these sites upon treatment with the CDK9 inhibitor AZD4573 and the CDK12/13 inhibitor SR4835 (Fig. 3a, b4c–f and Supplementary Fig. S7). Since both CDK9 and CDK12/13 phosphorylate the CTD of RNAPII at the same sites, inhibition of CTD-phosphorylation alone cannot account for apoptosis induction. Therefore, beyond RNAPII, CDK9 and CDK12/13 must target different proteins that enable productive elongation upon phosphorylation. Consequently, we focused on the recruitment of elongation factors to RNAPII. To study how AZD4573 (CDK9i) and SR4835 (CDK12/13i) affect the assembly of the RNAPII-elongation complex, we prepared chromatin-bound protein extracts from Jurkat cells and immunoblotted for RNAPII, RNAPII-pSer2 and the elongation factors SPT6, NELF, LEO1, and CDC73. This approach enables the monitoring of elongation factors associated with DNA-bound RNAPII and thus the composition of the DNA-associated elongation complex (Fig. 5a). As shown in Fig. 5b, c, AZD4573 (CDK9i) and SR4835 (CDK12/13i) reduced the amount of Ser2-phosphorylated RNAPII, though the amount of total RNAPII remained unchanged and rather increased temporarily after 3 h upon SR4835 treatment. Treatment with AZD4573 (CDK9i) strongly reduced the amount of chromatin-associated SPT6 and increased the amount of NELF after 3 h, whereas SR4835 (CDK12/13i) had no pronounced effect. Interestingly, inhibiting CDK9 with AZD4573 or CD12/13 with SR4835 significantly decreased the amount of chromatin-bound CDC73 and LEO1 of the PAF1C complex. Since inhibition of CDK9 disables the association of SPT6 and the dissociation of NELF, the PAF1C components CDC73 and LEO1 can obviously not bind to RNAPII (as NELF and PAF1C bind to RNAPII in a mutually exclusive manner [11]). As CDK12 is required for stable association of PAF1C with RNAPII [7], inhibition of CDK12/13 also disrupts the binding of CDC73 and LEO1 to RNAPII (Fig. 5b, c). These findings suggest that CDK9 inhibition targets different proteins (such as SPT6 and NELF) of the RNAPII machinery, as inhibition of CDK12/13 does. However, both inhibit the association of the PAF1C components, CDC73 and LEO1, albeit most likely through different mechanisms (Fig. 5a).

As CDK9 and CDK12/13 target different elongation factors within the RNAPII elongation machinery, this might explain why they cannot substitute for each other. Consequently, one might expect them to exhibit synergistic features. Accordingly, we performed synergy analyses in Jurkat and SUDHL1 cells and observed that the combination of AZD4573 (CDK9i) and SR4835 (CDK12/13i) displayed a high synergistic cytotoxicity (Fig. 5d–f and Supplementary Fig. 10). In contrast, the combination of AZD4573 or SR4835 with the transcriptional inhibitor actinomycin D or the translational inhibitor cycloheximide did not produce any synergistic effects (Fig. 5d–f and Supplementary Fig. S11). Likewise, we did not observe any synergy when combining AZD4573 or SR4835 with the Mcl1-inhibitor AZD5991. With regard to BS181, we only observed high ZIP scores and synergistic effects at high concentrations of BS181, where other CDKs (such as CDK9) were also affected (Supplementary Fig. S11).

### Knockdown of the pro-apoptotic Bcl-2 proteins Bax and Bak inhibits CDK9i and CDK12/13i induced apoptosis

**Fig. 6:**
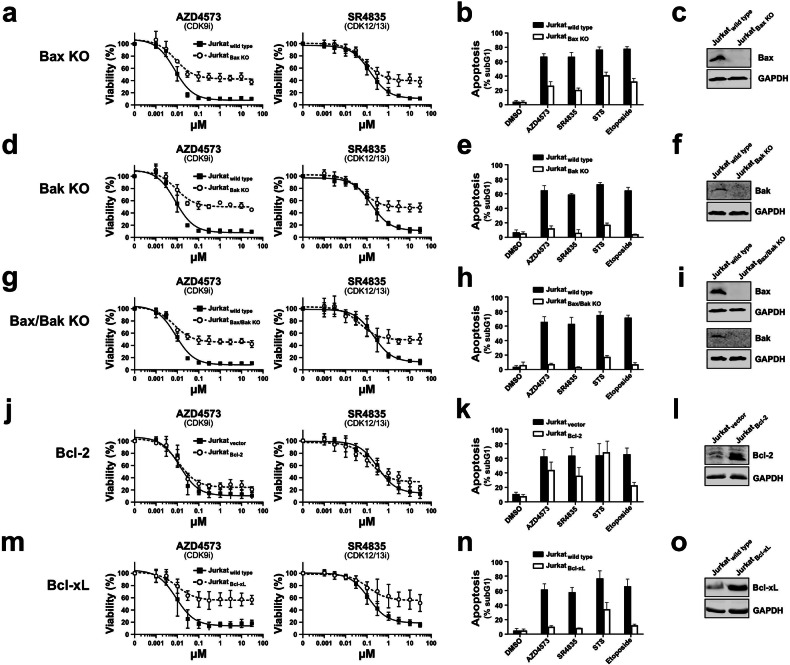
Knockdown of Bak and Bax, as well as overexpression of Bcl-xL, inhibits apoptosis induced by CDK9i and CDK12/13i, whereas Bcl-2 has no protective effect. Jurkat cells with a knockout of Bax (**a–c**), Bak (**d**–**f**), or Bax/Bak double knockout (**g–i**), as well as Jurkat cells overexpressing Bcl-2 (**j–l**) or Bcl-xL (**m–o**), and respective wild type or empty vector control cells, were tested for cell viability (**a**, **d**, **g**, **j**, **m**) or apoptosis induction (**b**, **e**, **h**, **k**, **n**). **a**, **d**, **g**, **j**, **m** Cell viability was assessed by AlamarBlue^®^ assay after 24 h. Error bars represent the mean ± SD from three independent experiments, performed in triplicate. **b**, **e**, **h**, **k**, **n** For apoptosis induction, cells were treated with AZD4573 (0.1 µM, CDK9i), SR4835 (2.5 µM, CDK12/13i), DMSO (0.1%), staurosporine (STS, 2.5 µM), or etoposide (50 µM), or DMSO (0.1%). After 24 h of incubation, apoptosis-related DNA degradation was detected via flow-cytometric measurement of propidium iodide stained apoptotic hypodiploid nuclei. Error bars represent the mean ± SD from three independent experiments, performed in triplicate. The respective knockout of Bax (**c**, **i**), Bak (**f**, **i**), or overexpression of Bcl-2 **l** or Bcl-xL **o** was monitored by immunoblotting. Representative immunoblots are shown.

Since the pro-apoptotic Bcl-2 proteins Bax and Bak exhibit the potential of binding and neutralizing Mcl1 and A1 [42, 43], we knocked out Bax, Bak, and Bax/Bak in Jurkat cells. Subsequently, we investigated in how far apoptosis induction by AZD4573 (CDK9i) and SR4835 (CDK12/13i) is affected in Bax-, Bak-, and Bax/Bak-double knockout cells (Fig. 6a–i). As shown in Fig. 6a, d, g, cell death induced by AZD4573 and SR4835 was strongly attenuated in Bax-, Bak-, and Bax/Bak-double knockout cells. Apoptosis induction was considerably reduced in Bax-deficient cells and completely abolished in Bak- and Bax/Bak-double knockout cells (Fig. 6b, e, h).

### Overexpression of the anti-apoptotic Bcl-2 protein Bcl-xL, but not of Bcl-2, inhibits CDK9i and CDK12/13i induced apoptosis

As shown in Figs. 2e and 6j–l, cytotoxicity and apoptosis induced by AZD4573 and SR4835 were only slightly reduced in Jurkat cells overexpressing Bcl-2. In contrast, apoptosis induction by AZD4573 and SR4835 was completely inhibited in Bcl-xL overexpressing Jurkat cells (Fig. 6m-o). However, when we overexpressed Mcl1 in Jurkat cells, cytotoxicity induced with AZD4573 and SR4835 was only slightly reduced, and apoptosis induction only attenuated in SR4835 treated cells (Supplementary Fig. S12a–e). Though Jurkat cells overexpressing Mcl1 showed a strong increase in Mcl1 expression at the mRNA level, protein expression was only slightly elevated, and treatment with AZD4573 induced Mcl1-degradation with similar rapid kinetics in both Jurkat-vector and Jurkat-Mcl1 cells (Supplementary Fig. S12f–h). Overexpression of A1 in Jurkat cells showed the same outcome, with a clear increase at both the protein and mRNA levels, but it had no effect on cell viability or apoptosis induction (Supplementary Fig. S12i–m). Thus, in contrast to Bcl-xL, which possesses a considerably longer half-life (Fig. 4c, e), overexpressed Mcl1 and A1 are evidently degraded by the proteasome to a comparable extent as their endogenous counterparts. Therefore, Jurkat cells overexpressing Mcl1 or A1 are only minimally protected from AZD4573 or SR4835-induced apoptosis, in contrast to Bcl-xL overexpressing cells (Fig. 6m–o).

## Discussion

CDK inhibitors are successfully applied in cancer therapy. However, the molecular mechanisms underlying apoptosis induction by CDK inhibitors remain a subject of debate. In addition to transcriptional CDK inhibitors, numerous reports state that CDK inhibitors targeting cell cycle control can also induce apoptosis [1, 15, 22–32]. CDK1 comprises the master regulator of the cell cycle and, unlike CDK2, CDK3, CDK4, and CDK6, is indispensable for cell cycle control [44]. However, besides induction of cell cycle arrest, neither the highly specific CDK1 inhibitor RO3306 nor the FDA-approved CDK4/6 inhibitors palbociclib and ribociclib displayed any cytotoxic or apoptotic potential in Jurkat leukemia cells, even after 72 h (Fig. 2a, b and Supplementary Fig. S1). Only inhibition of transcriptional elongation (by CDK9i and CDK12/13i), but not of transcriptional initiation (by CDK7i), induced cytotoxicity and apoptosis (Figs. 2a and 3c and Supplementary Figs. S1, S2, S3). This finding was unexpected since, besides its role in cell cycle control, CDK7 is considered a transcriptional master regulator. During initiation, CDK7 can phosphorylate Ser5 and Ser7 of the RNAPII CTD, which triggers Mediator dissociation and the exchange of initiation factors. It also directly activates the transcription-associated kinases CDK9, CDK12, and CDK13 and is required for the recruitment of the elongation factors DSIF and NELF, which initiate promoter-proximal pausing [1, 5, 6, 45]. Therefore, it was surprising that CDK7 inhibitors (such as BS181 and LDC4297) did not display any cytotoxic potential at concentrations at which they specifically target CDK7. Nevertheless, apoptosis induction upon CDK7 inhibition has been reported for solid tumors and hematological malignancies [39, 46–53]. As with CDK inhibitors that target cell cycle control, this may be an issue of specificity. Thus, we observed apoptosis induction with the CDK7 inhibitor THZ1 (Supplementary Fig. S2a–c), which confirms previous observations [46, 51, 52]. However, THZ1 also targets CDK12 and CDK13 at nanomolar concentrations, making it difficult to determine its specific effect on CDK7 (see Table 1). Similarly, cytotoxicity was only induced by BS181 and LDC4297 at high concentrations, which also inhibited CDK9 (Figs. 2a and 3c and Supplementary Figs. S1, S2, S3). This could explain previous reports indicating that CDK7 inhibitors induce cytotoxicity at micromolar concentrations of BS181 [39, 50].

Another unexpected finding was the observation that the CDK7 inhibitors BS181 and LDC4297 did not mediate the dephosphorylation of the CTD of RNAPII (Fig. 3a and Supplementary Figs. S4 and S5). CDK7 has been shown to mediate the phosphorylation of Ser5 and Ser7 within the heptad sequence of the CTD [1, 5]. However, several reports demonstrated that CDK7 may be entirely dispensable for CTD-phosphorylation and transcription. Although studies have shown that CDK7 deficiency results in severe mitotic defects, there is no evidence of a loss of CTD-phosphorylation [45, 54]. Similarly, application of the highly selective CDK7 inhibitor YKL-5-124 does not affect CTD-phosphorylation [55, 56].

Thus, CDK9 and CDK12/13 appear to be primarily responsible for CTD-phosphorylation and represent the central targets of CDKi-induced apoptosis. Inhibition of CDK9 (by AZD4573) or CDK12/13 (by SR4835) activated the intrinsic mitochondrial death pathway, as apoptosis induction was strongly attenuated in Apaf-1 knockdown Jurkat cells, and completely abrogated in Jurkat cells lacking caspase-9, as the central initiator caspase of the mitochondrial apoptosis pathway (Fig. 2c, d and Supplementary Fig. S3a, b, d, e).

**Fig. 7:**
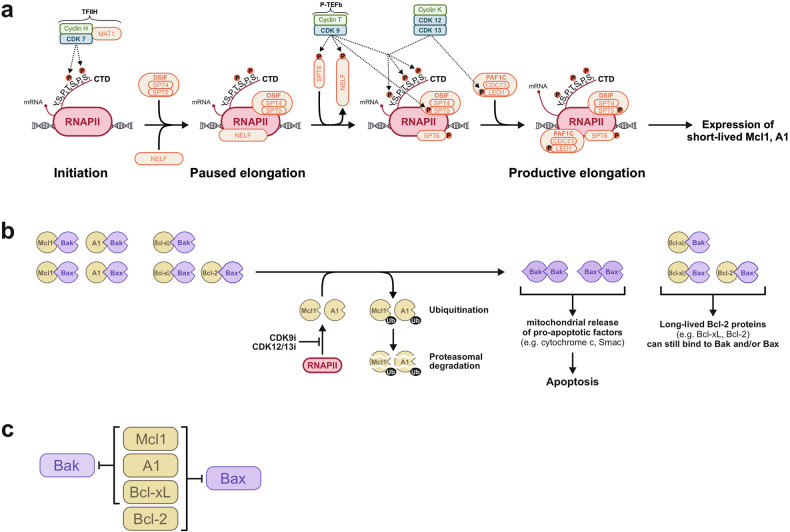
Schematic overview of RNAPII transcriptional activation and the mechanism of CDK9 and CDK12/13 inhibitor-induced apoptosis. **a** After CDK7 mediated initiation, transcription is provisionally paused by the association of two negative factors, DSIF and NELF. DSIF consists of the two proteins, SPT4 and SPT5. In its unphosphorylated state, DSIF promotes RNAPII pausing [9], whereas phosphorylation by CDK9 can switch SPT5 from a repressor to an activator of transcription [1, 5, 10]. In addition to SPT5, CDK9 also phosphorylates SPT6 and NELF. This leads to the recruitment of SPT6 to RNAPII and the subsequent release of the roadblock NELF. The release of NELF enables the binding of PAF1C, since both complexes share a common binding interface on RNAPII [11]. PAF1C consists of the subunits PAF1, LEO1, CDC73, CTR9, WDR61 and Rtf1. The PAF1C component LEO1 is a substrate of CDK12 [12], and both LEO1 and CDC73 are destabilized upon inhibition of CDK12 [7]. The binding of PAF1C and the phosphorylation of LEO1 then promote productive elongation [8, 12, 13]. However, the extent to which CDK12/13 enables the recruitment of PAF1C or stabilizes the association of PAF1C with RNAPII remains unclear. Inhibition of CDK9 and CDK12/13 abrogates productive elongation of RNAPII and prevents the expression of short-lived proteins, such as Mcl1 and A1. **b** The anti-apoptotic Bcl-2 proteins Mcl1, A1, and Bcl-xL bind to and neutralize the pro-apoptotic Bcl-2 proteins Bax and Bak. In contrast, Bcl-2 can only bind to Bax, but not Bak. Inhibition of CDK9 or CDK12/13 interrupts transcription of the short-lived Mcl1 and A1 proteins, whereas the expression of Bcl-2 and Bcl-xL remains unaffected. Mcl1 and A1 are rapidly degraded by the proteasome. Consequently, Bax and Bak are no longer neutralized by Mcl1 or A1, which shifts the balance toward the induction of apoptosis by the Bax/Bak-mediated mitochondrial release of pro-apoptotic factors, such as cytochrome c and Smac. **c** Within the Bcl-2 family, Mcl1, A1, and Bcl-xL inhibit Bax and Bak, whereas Bcl-2 can only inhibit Bax. Accordingly, CDK9 and CDK12/13 inhibitors can target Mcl1 and A1 and display the potential to induce apoptosis in Bcl-2 overexpressing tumor cells (modified from Soderquist et al. [42]).

Though both CDK9 and CDK12/13 inhibition can induce apoptosis, the conundrum remains: Why can’t CDK9 substitute for CDK12/13, and vice versa? Both CDK9 and CDK12/13 phosphorylate the CTD of RNAPII at Ser2, Ser5, and Ser7 [5]. Accordingly, we observed the dephosphorylation of these sites following treatment with the CDK9 inhibitor AZD4573 and the CDK12/13 inhibitor SR4835 (Figs. 3a, b and 4c–f and Supplementary Fig. S7). Thus, it is evident that the inhibition of CTD-phosphorylation alone cannot account for the induction of apoptosis, given that both CDK9 and CDK12/13 phosphorylate the CTD of RNAPII at the same sites. Consequently, CDK9 and CDK12/13 must target different proteins that enable productive elongation upon phosphorylation. In this context, recent structural studies of RNAPII have provided novel insights into the dissolution of promoter-proximal pausing [8, 9]. Following initiation, transcription is provisionally paused 20 - 60 bp downstream of the transcription start site by the association of the elongation factors DSIF and NELF [6, 9]. In its unphosphorylated state, DSIF has been shown to promote RNAPII pausing [9]. However, phosphorylation of SPT5 within DSIF by CDK9 results in a transition from a repressor to an activator of transcription [1, 5, 10]. In addition to SPT5, CDK9 has also been shown to phosphorylate SPT6 and NELF. This results in the recruitment of SPT6 to the RNAPII complex and the release of the roadblock NELF [8, 11]. The release of NELF enables the binding of PAF1C, as both complexes share a common binding interface on RNAPII [11]. PAF1C is crucial for the release of transcriptional pausing and consists of the subunits PAF1, LEO1, CDC73, CTR9, WDR61 and Rtf1. It has been shown that the subsequent recruitment of CDK12 is dependent on PAF1C [13] and that CDC73 is required for the activation of CDK12/13 during transcription elongation [57]. In addition, Qiu et al. demonstrated that LEO1 is a bona fide substrate of CDK12 and depletion of LEO1, or substituting LEO1 phosphorylation sites with alanine, attenuated PAF1C association with RNAPII and impaired transcription elongation [12]. Additionally, inhibiting CDK12 has been shown to result in the loss of the association of LEO1 and CDC73 with RNAPII [7]. Thus, these data imply that, although CDK12 may not be responsible for recruiting PAF1C, it is crucial for stabilizing the interaction between PAF1C and RNAPII (Fig. 7a).

Accordingly, by monitoring chromatin-associated proteins, we could show that treatment with AZD4573 (CDK9i) strongly reduced the amount of SPT6 and increased the amount of NELF, whereas SR4835 (CDK12/13i) had no pronounced effect (Fig. 5b, c). The observation that both AZD4573 (CDK9i) and SR4835 (CDK12/13i) inhibit the binding of the PAF1C components, CDC73 and LEO1, to RNAPII could be explained as follows: Since NELF and PAF1C bind to RNAPII in a mutually exclusive manner [11], the PAF1C subunits, CDC73 and LEO1, cannot bind to RNAPII when NELF remains associated with RNAPII following CDK9 inhibition. As CDK12 is necessary for the stable association of PAF1C with RNAPII [7], inhibiting CDK12/13 abrogates the interaction of the PAF1C components CDC73 and LEO1 with RNAPII (Fig. 5b, c). Thus, both inhibition of CDK9 and CDK12/13 prevent the association of the PAF1C subunits, CDC73 and LEO1, albeit through different mechanisms (Fig. 7a). These findings underscore that CDK9 inhibition targets different elongation factors (such as SPT5, SPT6, and NELF) of the RNAPII machinery, as is the case with CDK12/13 inhibition (e.g., LEO1). This may explain why they cannot substitute for each other, as productive transcription elongation requires the cooperative action of both CDK9 and CDK12/13. Accordingly, we observed that the combination of AZD4573 (CDK9i) and SR4835 (CDK12/13i) displayed a pronounced synergistic cytotoxicity in Jurkat and SUDHL1 cells (Fig. 5d–f and Supplementary Fig. S10).

If inhibition of RNAPII is indeed the predominant mechanism by which CDK inhibitors induce apoptosis, then this should be mediated by the downregulation of short-lived anti-apoptotic proteins, as described for cFLIP, XIAP, and anti-apoptotic Bcl-2 proteins (such as Bcl-xL, Bcl-2, Mcl1, and A1) [20, 21, 38, 58–65]. Inhibition of CDK9 has been shown to downregulate the death receptor antagonist cFLIP (FLICE-like inhibitor protein), which sensitizes non-small cell lung cancer cell lines to treatment with the death receptor ligand TRAIL [58]. However, cFLIP can be ruled out as we previously demonstrated that the pan-CDK inhibitors meriolin16, 31, and 36 can induce apoptosis in Jurkat cells lacking caspase-8, which is the central initiator caspase of the death receptor pathway [37]. In addition, the caspase-3, -7, and -9 inhibitor XIAP (X-linked inhibitor of apoptosis), Bcl-2, and Bcl-xL could also be excluded, as they are stable within 6–8 h and are only slightly degraded after 24 h (Figs. 3a and 4c, e and Supplementary Fig. S7a,c), which has also been shown by other groups [20, 38, 66]. Therefore, the most likely candidates appear to be the two short-lived anti-apoptotic Bcl-2 proteins Mcl1 and A1. Studies using pan-CDK inhibitors [38, 66, 67] or CDK9-specific inhibitors [19, 20] have demonstrated the downregulation of Mcl1. Furthermore, Boiko et al. demonstrated that another Bcl-2 protein, A1/Bfl1, could be downregulated by the CDK9 inhibitor AZD4573 [21]. We corroborated these findings and demonstrate that, in addition to CDK9 inhibitors, inhibition of CDK12/13 by SR4835 also induced the rapid downregulation of Mcl1 and A1 within 3 - 4 h in Jurkat leukemia and SUDHL1 lymphoma cells (Fig. 4c–f).

As demonstrated by Boiko et al., A1 (in contrast to Mcl1) displays a rather limited expression pattern, which is restricted to hematopoietic cancer cell lines, with a higher prevalence in lymphoma than in myeloma or leukemia cell lines [21]. Unlike Mcl1, which was found to be highly expressed in various lymphoma cell lines, only a subset (17 of 52) were A1-positive [21]. Both A1 and Mcl1 have short mRNA and protein half-lives [20, 68–72] and are rapidly degraded by the proteasome, as shown in cycloheximide chase experiments that were inhibited upon addition of the proteasome inhibitor MG132 [21]. In a similar manner, MG132 also inhibited Mcl1 and A1 degradation upon treatment with AZD4573 (CDK9i) or SR4835 (CDK12/13i) (Supplementary Fig. S9). To date, four different E3 ubiquitin-ligases (e.g., Mule, SCF^β-TrCP^, SCF^Fbw7^, and Trim17) have been described to mediate Mcl1 ubiquitination [73]. However, Stewart et al. used an Mcl1 mutant lacking the lysine residues required for ubiquitination and found that it was degraded at a similar rate to wild-type Mcl1. This finding suggests that the proteasomal degradation of Mcl1 can obviously occur independently of ubiquitination [74].

Although CDK9, CDK12, and CDK13 cooperate in the expression of Mcl1 and A1, it is noteworthy that they also exhibit distinct gene expression profiles. Thus, CDK9 inhibition results in depletion of short-lived survival transcripts and broad repression of immediate-early genes. In contrast, CDK12/13 are particularly critical for the transcription of long, complex genes, many of which are involved in DNA damage response (DDR) [1].

Notably, Harper et al. recently demonstrated that apoptosis mediated by α-amanitin- or triptolide-induced degradation of RNAPII is not the result of transcriptional inhibition [75]. They showed that the multifunctional RNA-binding protein PTBP1 and the BH2 homology domain-containing protein BCL2L12 are primarily localized in the nucleus of viable cells, where PTBP1 interacts with the hypophosphorylated form of RNAPII. Upon α-amanitin- or triptolide-induced loss of hypophosphorylated RNAPII, PTBP1 promotes the translocation of BCL2L12 into the cytosol. There, BCL2L12 acts as a pro-apoptotic Bcl-2 protein that activates the intrinsic mitochondrial death pathway. The authors termed this novel pathway “Pol II degradation-dependent apoptotic response” (PDAR) [75]. However, the molecular mechanism of apoptosis induction upon CDK9 and CDK12/13 inhibition obviously differs from PDAR, as RNAPII does not become downregulated within 6–8 h (Figs. 3a, b, 4c, e and 5b, c and Supplementary Fig. S7a, c), when caspase activation and PARP-cleavage already occurred.

Interestingly, SUDHL1 cells and Jurkat cells (which, unlike SUDHL1 cells, express no A1, but a similar amount of Mcl1) displayed equal sensitivity to CDK9i and CDK12/13i. However, SUDHL1 cells were much more resistant when treated with the Mcl1-inhibitor AZD5991 (Fig. 4b). Conversely, Boiko et al. demonstrated that siRNA knockdown of A1 in SUDHL1 cells increased the sensitivity towards the Mcl1 inhibitor AZD5991 [21]. This suggests that A1 can partially substitute for the anti-apoptotic effect of Mcl1.

Both Mcl1 and A1 counteract the two pro-apoptotic Bcl-2 proteins Bax and Bak. Consequently, the induction of apoptosis by AZD4573 or SR4835 was blocked in Bak- and Bax/Bak-deficient Jurkat cells and strongly reduced in Bax-deficient cells (Fig. 6b, e, h). Similarly, Cidado et al. demonstrated that siRNA-mediated knockdown of both Bax and Bak in OCI-LY10 lymphoma cells completely abolished AZD4573-induced caspase activation. Knockdown of Bax reduced caspase activation, but not as potently as Bak knockdown [20]. This indicates that AZD4573 (CDK9i) and SR4835 (CDK12/13i) primarily target Bak and, to a lesser extent, Bax. As Bax and Bak are also antagonized by Bcl-xL we observed the inhibition of apoptosis induction with AZD4573 (CDK9i) and SR4835 (CDK12/13i) in Bcl-xL overexpressing Jurkat cells (Fig. 6m–o). Given its prolonged half-life relative to Mcl1 or A1, Bcl-xL is evidently not subject to proteasomal degradation. Consequently, it can substitute for downregulated Mcl1 and A1 by counteracting Bax and Bak. However, when we overexpressed Mcl1 in Jurkat cells, we observed only a slight reduction in apoptosis upon treatment with AZD4573 (CDK9i) or SR4835 (CDK12/13i), whereas A1 overexpression displayed no protection. Though Mcl1 overexpressing Jurkat cells displayed high mRNA levels, protein expression was only slightly increased (Supplementary Fig. S12f, g). Thus, it appears that, irrespective of protein expression level, the proteasome degrades endogenous as well as overexpressed Mcl1 and A1 to the same extent. Therefore, overexpressed Mcl1 is degraded in the same kinetics as endogenous Mcl1, resulting in only a slight reduction in cytotoxicity and apoptosis induction (Supplementary Fig. S12). Nevertheless, other groups have observed a more pronounced inhibition of apoptosis upon Mcl1 overexpression using different cell lines [21, 40, 76].

Most interestingly, as we could previously show for the pan-CDK inhibitors meriolin16, 31, and 36 [37], AZD4573 (CDK9i) and SR4835 (CDK12/13i) were able to induce apoptosis in Bcl-2 overexpressing Jurkat cells (Figs. 2e and 6j–l). Unlike Bcl-2, which binds exclusively to Bax, Mcl1, A1, and Bcl-xL interact with both Bax and Bak [42, 43]. This explains why Bcl-2 (unlike Bcl-xL) cannot protect against apoptosis induced by AZD4573 (CDK9i) or SR4835 (CDK12/13i), since Bcl-2 can only neutralize Bax, not Bak (Fig. 7b, c).

In addition to targeting transcriptional addiction in cancer, the particular property of CDK9 and CDK12/13 inhibitors to induce apoptosis in Bcl-2 overexpressing tumor cells renders them promising anticancer drugs for overcoming therapy resistance. Unlike cell cycle inhibitors (such as CDK4/6 inhibitors), transcriptional CDK inhibitors have not yet been incorporated into routine clinical treatment regimens. Nevertheless, their potential is quite promising, as tumors frequently overexpress anti-apoptotic Bcl-2 proteins to gain therapy resistance. To neutralize anti-apoptotic Bcl-2 proteins, BH3 mimetics have been developed that target pro-survival Bcl-2 proteins. The selective Bcl-2 inhibitor venetoclax (Venclexta, ABT-199, GDC-0199) has received approval for the treatment of various forms of leukemia [77, 78], and selective Mcl1-inhibitors are also in clinical development for the treatment of hematological diseases [79]. However, it has been shown that tumor cells achieve resistance to Bcl-2 targeting BH3 mimetics (such as venetoclax, ABT-263 (navitoclax), or ABT-737) by overexpressing Mcl1, Bcl-xL, or A1 [79–83]. Consequently, transcriptional CDK inhibitors emerge as a promising alternative to BH3 mimetics. Taken together, our comprehensive study provides new insights into the role of CDK inhibitors in apoptosis induction and underscores the potential of transcriptional CDK inhibitors as promising anticancer drugs.

## Materials and methods

### Reagents

Meriolin16 (4-(4-methoxy-1H-pyrrolo2,3-b] pyridine-3-yl)pyridine-2,6-diamine) was synthesized by the group of Prof. Dr. T. J. J. Müller (Institute of Organic Chemistry of Heinrich Heine University Düsseldorf) and described in [36, 37]. The pan-caspase inhibitor N-(2-Quinolyl)valyl-aspartyl-(2,6-difluorophenoxy)methyl ketone (Q-VD-OPh, QVD, #S7311), dinaciclib (#S2768), RO-3306 (#S7747), palbociclib (#S4482), ribociclib (#S7440), and the selective inhibitors BS181 (#S1572; CDK7i), SR4835 (#S8894; CDK12/13i), and AZD4573 (#S8719; CDK9i) were obtained from Selleckchem (Houston, TX, USA). SNS032 (#SML2218; pan-CDKi), the translational inhibitor cycloheximide (#01810), and the proteasomal inhibitor MG132 (#474790) were obtained from Sigma-Aldrich (St. Louis, MO, USA). Actinomycin D (#11805017) was obtained from Fisher Scientific (Hampton, NH, USA). LDC4297 (LDC44297; CDK7i), atuveciclib (LDC208447; CDK9i), THZ531 (LDC208434; CDK12/13i), and THZ1 (LDC198304; CDK7i) were provided by LDC (Lead Discovery Center Dortmund, Germany). The Mcl1 inhibitor (AZD5991; HY-101533) was obtained from MedChemExpress (Monmouth, NJ, USA). Venetoclax (HY-15531) was obtained from Hoelzel (Cologne, Germany). Staurosporine was purchased from Biozol (#S-9300), and etoposide (#1043) was obtained from Biovision (Waltham, MA, USA). All other reagents for which a manufacturer is not explicitly specified were obtained from Carl Roth (Karlsruhe, Germany).

### Cell lines and cell culture

Jurkat cells (human T cell acute lymphoblastic leukemia (T-ALL); #ACC-282), HeLa cells (human cervix carcinoma; ‘ACC-57), SUDHL1 cells (anaplastic large cell lymphoma, #ACC 356), HL60 (human acute myeloid leukemia; #ACC-483) and SUPB15 (human B cell acute lymphoblastic leukemia; #ACC-389) were obtained from the German Collection of Microorganisms and Cell Cultures (DSMZ). KOPTK1 (human T-ALL; CVCL-4965) was kindly provided by Oskar Haas (Children’s Cancer Research Institute, St. Anna Children’s Hospital, Vienna, Austria). Jurkat cells with a deficiency in caspase-9 were supplied by Klaus Schulze-Osthoff (Interfaculty Institute for Biochemistry, University of Tübingen, Germany) [37] and underwent retrovirus transduction with either an empty pMSCVpuro (Clontech, Heidelberg, Germany) or a pMSCVpuro containing cDNAs encoding for untagged human wild-type caspase-9, as previously described [37]. Bcl-2 and Bcl-xL overexpressing Jurkat cells and the corresponding vector control cells were provided by Claus Belka (Ludwig-Maximilians University, Munich, Germany) and have been previously described [37, 84]. The generation of Jurkat cells with Apaf-1 knockdown was accomplished through the utilization of CRISPR/Cas, as previously described [37]. HeLa cells were cultured in high-glucose Dulbecco’s Modified Eagle’s medium (DMEM), and the suspension cells were cultured in RPMI medium. The medium was supplemented with 10% FCS, 10 mM HEPES, 100 U/ml penicillin, and 100 µg/ml streptomycin, and the cells were cultured at 37 °C in a humidity-saturated atmosphere 5% CO_2_. The knockout of Bax and Bak in Jurkat cells was established using CRISPR/Cas9. For lentivirus production, 1 × 10^7^ HEK2983FT cells /10 cm dish were transfected with 10 µg pMD2.G, 15 µg psPAX2, and 20 µg plentiCRISPRv2 containing the desired guideRNA (*bax*: AAACTCACCCCTGAAGCAAA; *bak1*: GCTCACCTGCTAGGTTGCAG) [85] using PEI (polyethylenimine 40,000; Warrington, PA, USA) reagent (1 mg/mL PEI in 25 mM HEPES pH 7.5 and 150 mM NaCl). Virus-containing supernatant was collected 48 h and 72 h after transfection and concentrated by ultracentrifugation. Jurkat cells were transduced with 10 µl concentrated virus overnight and then cultured with 1 µg/ml puromycin (Thermo Fisher Scientific) for 48 h. Cells were cultured in the presence of puromycin for 4 weeks before experiments were performed. Overexpression of Mcl1 and A1 in Jurkat cells was accomplished by lentiviral transduction. Lentiviruses were generated by transfecting HEK293T cells with packaging plasmids pMDLg/pRRE (Addgene#12251), pRSV-Rev (Addgene#12253), and pMD2.G (Addgene#12259). For MCL1, cells received lentiviral particles carrying the transfer plasmid pLenti CMV MCL1 Puro (Addgene#140746). In the case of A1, the transfer plasmid was created by inserting the A1 cDNA (Promega#FXC03081) into the NheI and NotI digested pCDH-Bcl-XL vector (Addgene#46972). Lentiviral supernatant was harvested 48 hours after transfection, filtered through a 0.4 µm filter, and concentrated with Lenti-X Concentrator (Takara#631232). Jurkat cells (2 × 10^6^ per well in a 6-well plate) were transduced in the presence of 10 µg/ml polybrene (Sigma-Aldrich#TR-1003-G). After incubating for 48 h, the medium was changed, and cells were selected using puromycin (2 µg/ml, InvivoGen#ant-pr-1) for 3 days. All cell lines were tested for mycoplasma contamination.

### Cell viability assay

Cell viability in Jurkat and SUDHL1 cells was assessed via a resazurin reduction assay, also known as AlamarBlue® assay. The cells were initially seeded at a density of 5 × 10^5^ cells/well and subsequently exposed to increasing concentrations of the substance of interest. Following a 22 h incubation period, resazurin (Sigma, #R7017) was added to a final concentration of 40 µM. After an incubation period of 120 minutes, the fluorescence of resorufin (excitation: 560 nm, emission: 590 nm) was measured using a microplate spectrophotometer. The reduction of resazurin to resorufin is proportional to aerobic respiration, thereby serving as an indicator of cell viability. Viability curves were created using Graphpad Prism 8, and the respective IC_50_ was calculated.

### Assessment of cytotoxic synergy in combination treatment

The effects of substance combinations were analyzed by creating and subsequently combining substance concentration series on 96-well plates. This method ensured that each concentration of one substance was combined with each concentration of the other substance. Cells were then added and further processed according to the standard protocol of viability measurement using resazurin staining. For each concentration of one substance, a viability curve of the other substance was generated, and the resulting IC_50_ values were visualized using an isobologram. Furthermore, a Synergy Score was calculated for each concentration of the substance combination using SynergyFinder and displayed graphically using GraphPad Prism 8.

### Flow-cytometry based analysis of apoptotic cell death and cell cycle

The measurement of DNA content during the cell cycle and the presence of hypodiploid apoptotic nuclei were performed according to the method established by Nicoletti et al. [86]. The preparation of nuclei involved the lysis of cells in a hypotonic lysis buffer composed of 1% sodium citrate, 0.1% Triton X-100, and 50 µg/ml propidium iodide, employed for the staining of the DNA. Subsequently, the analysis of nuclei was performed through flow cytometry. To ensure accurate and precise measurements of the cell cycle, the prepared nuclei were analyzed using linear mode, a method that clearly differentiates G_1_-, S-, G_2_/M-phase, and hypodiploid nuclei (HN). The measurement of hypodiploid nuclei in logarithmic mode was utilized for the determination of apoptotic cells. The LSRFortessaTM (Becton, Dickinson, Heidelberg, Germany) was utilized for the flow-cytometry analysis, and FlowJo_V10 (BD Biosciences) was used for the data analysis.

### Immunoblotting

Cells were seeded at a density of 2 × 10^6^ cells/ml in 2 ml, treated as indicated, and incubated for the specified time period. Given the significantly higher protein requirements for the isolation of both chromatin-bound and unbound proteins, a 5-ml volume was employed in this particular experiment. Subsequently, the cells were harvested by centrifugation (3000 rpm, 5 min, 4 °C) and the cell pellet stored at -20°C. The lysis of cells was achieved by employing 40 µl of lysis buffer, which comprised the following components: 20 mM Tris-HCl, 150 mM NaCl, 1% v/v Triton X-100, 0.5 mM EDTA, 10 mM NaF, 2.5 mM Na_4_P_2_O_7_, PhosStop (Sigma, 4906837001), and protease inhibitor (Sigma, P2714). Benzonase (5 U/ml, #70664-3; Sigma-Aldrich) was added to all samples (except those undergoing separate lysis) to degrade the DNA, thereby enabling the examination of DNA-bound proteins. The sample underwent a 30 min incubation period, followed by a centrifugation process (13,300 rpm, 15 min, 4 °C). The final sample, or the chromatin unbound fraction, was then separated as the supernatant. In addition, two washing steps and a further lysis with the addition of benzonase were carried out for the separation of chromatin-bound and unbound proteins. A subsequent analysis of the protein concentration of all samples was conducted by the Bradford assay, after which the samples were diluted in the respective lysis buffer with Laemmli Buffer. The SDS-PAGE and immunoblot procedures were conducted in accordance with standard workflows. These procedures entailed the application of target protein-specific primary antibodies, which were diluted in 1x TBS-T, supplemented with 0.05% NaN3 and 5% BSA, specifically anti-Bfl1/A1 (Cell Signaling, #14093S, 1:500), anti-Bcl-2 (Cell Signaling, #4223S, 1:1000), anti-Bcl-xL (Cell Signaling, #54H6, 1:1000), anti-CDC73 (Fisher Scientific, PA5-26189, 1:1000), anti-GAPDH (glyceraldehyde 3-phosphate dehydrogenase; Abcam, ab8245, 1:5000), anti-Histon H3 (Cell Signaling, #14269, 1:5000), anti-LEO1 (Proteintech, #12281-1-AP, 1:1000), anti-Mcl1 (Cell Signaling, #94296S, 1:500), anti-NELF (Proteintech, #10705-1-AP, 1:2000 anti-PARP (Cell Signaling Technology, #9542S, 1:1000), anti-RNA Pol. II (Santa Cruz, sc-56767, 1:1000), anti-RNA pol. II Ser2 (Abcam, ab5095, 1:1000), anti-RNA pol. II Ser5 (Abcam, #ab5408, 1:500), anti-RNA pol. II Ser7 (abcam, ab252853, 1:500), anti-SPT6 (Novus Biologicals, NB100-2582, 1:1000), anti-vinculin (Sigma, V9131, 1:2000), and anti-XIAP (BD Bioscience, #610762, 1:1000). Protein detection on a nitrocellulose membrane using the LI-COR Odyssey® imaging system was performed using fluorescence coupled secondary antibodies (LI-COR Biosciences). Determination of protein band density was performed using Image StudioTM Lite Version 5.2. Normalization of protein density was conducted relative to the respective loading control (vinculin or GAPDH). To calculate the magnitude of the observed change in protein expression, each normalized ratio was divided by the normalized band of the DMSO control. This calculation yielded a fold change of 100% for the control sample. The results were then analyzed and represented graphically using GraphPad Prism 8.

### Microscopy-based analysis of EU-incorporation

For the analysis of newly synthesized RNA, the Invitrogen Click-iT® RNA imaging kit (#C10329) was used in accordance with the manufacturer’s protocol. 5-ethynyl-uridine (EU) is a non-natural base analog of uracil that is used to directly map global transcription, both temporally and spatially. This enables the visualization of transcriptional activity and the amount of newly synthesized RNA. HeLa cells were seeded at a density of 0.1 × 10^6^ cells/ml and treated on the next day with the specific stimuli for 24 h. After that incubation time, the EU solution was added to a final concentration of 1 mM. The cells were then incubated for an additional hour, during which the EU can incorporate into the newly synthesized RNA. Therefore, only the de novo RNA synthesis of this timeframe is analyzed. The cells were subjected to fixation and permeabilization protocols, followed by analysis of EU detection in conjunction with Hoechst-stained nuclei under the confocal microscope. The Images were analyzed with Fiji, and the average fluorescent intensity per cell was calculated. Afterwards, the fluorescent intensity was averaged per replicate, and the intensity of the blank images (pictures without EU, but with Hoechst staining and fluorescent dye, which binds to the EU) was subtracted. The intensity was normalized to the DMSO control for each replicate.

### Fluorometric caspase-3 activity assay

Jurkat cells were seeded at a density of 0.5 × 10^5^ cells/well and treated with a specific CDK inhibitor for 0–8 h. After this incubation time, the cells were harvested by centrifugation (600 × *g*, 5 min, 4 °C) and stored at −80 °C. Following the thawing process 50 µl of ice-cold lysis buffer (20 mM HEPES, 84 mM KCl, 10 mM, MgCl_2_, 200 μM EDTA, 200 μM EGTA, 0.5% NP40, 1 μg\/ml leupeptin, 1 μg\/ml pepstatin, 5 μg\/ml aprotinin) were added per well. The lysis was performed on ice for 10 min. Subsequently, 150 µl of reaction buffer (50 mM HEPES, 100 mM NaCl, 10% sucrose, 0.1% CHAPS, 2 mM CaCl₂, 13.35 mM DTT, 50 µM Ac-DEVD-AMC) was added to each well. To assess the kinetics of AMC release, the AMC fluorescence intensity (excitation: 360 nm; emission: 450 nm) was measured at 37 °C at 2 min intervals for a total of 2 h using a Synergy Mix microplate reader. By measuring the slope of the linear range of the fluorescence increase (∆rfu/min), it was possible to determine the level of DEVDase activity. This value was then normalized to the DMSO control. Determination of the fluorescence of the profluorescent caspase-3 substrate DEVD-AMC, and consequently DEVDase activity, was regarded as a marker of caspase-3 activity.

### Reverse transcription quantitative PCR

Total RNA extraction was performed using the ReliaPrep™ RNA Miniprep Kit (#Z6012, Promega). One microgram (1 µg) of RNA was reverse transcribed into cDNA using M-MLV Reverse Transcriptase (#M1701, Promega) with random hexamers. The resulting cDNA was subjected to amplification by quantitative reverse transcription PCR (RT-qPCR) using Promega GoTaq PCR Master Mix (#M7132, Promega). The RT-qPCR analysis was performed with the CFX Opus 384 Real-Time PCR System (Bio-Rad, Hercules, CA, USA). For accurate interpretation of RT-qPCR data, all primers were tested for primer–template binding efficiency. RT-qPCR results were evaluated using the 2^−ΔΔCq^ method, with GAPDH and PGK1 expression levels as housekeeping gene controls. The following primers were used: GAPDH: forward primer 5’-3’: GTCAGCCGCATCTTCTTTTG; reverse primer 5’-3’: GCGCCCAATACGACCAAATC; PGK1: forward primer 5’-3’: GACAGCAGCCTTAATCCTCTG; reverse primer 5’-3’: CTAACAAGCTGACGCTGGA; BCL2A1: forward primer 5’-3’: AATTGCCCCGGATGTGGATA; reverse primer 5’-3’: TTTTCCCAGCCTCCGTTTTG; MCL1: forward primer 5’-3’: GAGACCTTACGACGGGTTGG; reverse primer 5’-3’: GAGAGTCACAATCCTGCCCC.

### Statistical analyses

Error bars represent the standard deviation. Statistical analysis was carried out using two-way ANOVA, with significance levels of *p ≤ 0.05; **p ≤ 0.01; ***p ≤ 0.001. If not stated otherwise, all statistical analyses were conducted using Prism v8.02 (GraphPad Software, La Jolla, CA, USA).

### Ethics approval and consent to participate

(a) All methods were performed in accordance with the relevant guidelines and regulations. (b) Approval from ethics committee: Does not apply, as no animals or human subjects were involved in this work. (c) Informed consent was obtained from all participants. (d) Publication of identifiable images from human research participants: Does not apply.

## Supplementary information

Supplemental Figures

Original Immunoblots

## Data Availability

Data were generated by the authors and included in the article.
