# Supplementary material for: Inhibition of RNA polymerase II-activating CDK9 and CDK12/13, but not of cell cycle relevant CDKs, induces apoptosis by downregulating the short-lived Bcl-2 proteins Mcl1 and Bfl1/A1
Source: Cell Death Dis. 2026 May 27;17(1):512. doi: 10.1038/s41419-026-08889-6 (PMC13216340; doi:10.1038/s41419-026-08889-6)
Supplement: Supplementary file 1 — Supplemental Figures [file 41419_2026_8889_MOESM1_ESM.pdf]

Supplemental Information

Supplemental Figures

Supplemental Figure S1: Inhibition of cell cycle control (CDK1i or CDK4/6i) or transcriptional initiation (CDK7i) does not induce cytotoxicity or apoptosis after 72 h

Supplemental Figure S2: Inhibition of transcriptional elongation (CDK9i and CDK12/13i), but not of transcriptional initiation (CDK7i) induces cytotoxicity and apoptosis

Supplemental Figure S3: Apoptosis induced by pan-specific and transcriptional CDK inhibitors, but not by cell cycle-specific CDK inhibitors, is dependent on caspase-9 and Apaf1, but largely independent on Bcl-2

Supplemental Figure S4: Effects of different CDK7 inhibitors on RNA polymerase II phosphorylation and Mcl1 expression in Jurkat cells

Supplemental Figure S5: CDK7 inhibition by BS181 does not affect RNA polymerase II phosphorylation or Mcl1 expression in different leukemia cell lines

Supplemental Figure S6: Transcriptional inhibition by CDK9 and CDK12/13 inhibitors is independent of caspase activity in HeLa cells

Supplemental Figure S7: CDK9 and CDK12/13 inhibitor-induced reduction of Mcl1, A1, and RNA polymerase II phosphorylation occurs independently of caspase activity in Jurkat and SUDHL1 cells

Supplemental Figure S8: Inhibition of CDK7 has no effect on the downregulation of the anti-apoptotic Bcl-2 proteins Mcl1 and A1/Bfl1

Supplemental Figure S9: Proteasome inhibition by MG132 prevents the reduction of short-lived proteins Mcl1 and A1 induced by CDK9 and CDK12/13 inhibitors

Supplemental Figure S10: Inhibitors of CDK9 and CDK12/13 act synergistically in cell death induction

Supplemental Figure S11: Combinatorial treatment of AZD4573 or SR4835 with the translational inhibitor cycloheximide, the Mcl1 inhibitor AZD5991, or BS181 (CDK7i) does not display synergy in Jurkat and SUDHL1 cells

Supplemental Figure S12: Effect of Mcl1 overexpression on CDK9 and CDK12/13 inhibitor-induced cytotoxicity and apoptosis in Jurkat cells

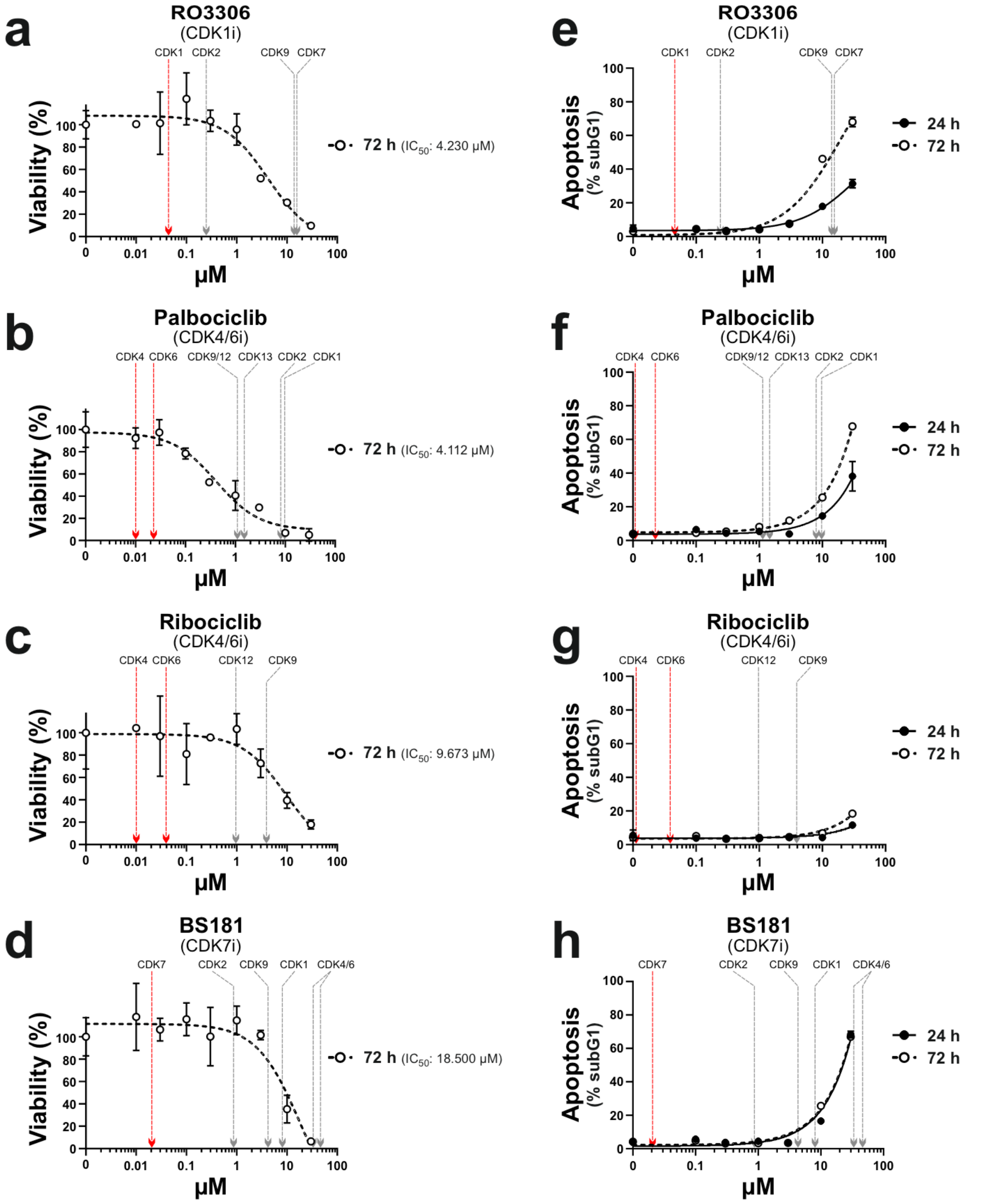

Supplementary Fig. S1: Inhibition of cell cycle control (CDK1i or CDK4/6i) or transcriptional initiation (CDK7i) does not induce cytotoxicity or apoptosis, even after after 72 h

(a-d) Cell viability of Jurkat cells treated with the indicated CDK inhibitors was measured by AlamarBlue® assay after 72 h. The cytotoxicity IC_{50} values are shown in the curve labeling (in parentheses). The experiments were performed in addition to the 24 h stimulation, as shown in Fig. 2a. Error bars represent the mean ± SD from one experiment, performed in triplicate. **(e-h)** Jurkat cells were treated with with the indicated CDK inhibitors for 24 h and 72 h. Apoptosis was assessed by flow cytometric measurement of apoptotic hypodiploid nuclei. Error bars represent the mean ± SD from one experiment, performed in triplicate. **(a-h)** The enzymatic IC_{50} values of CDK specific inhibitors (see Table 1) are indicated by red and gray arrows, respectively.

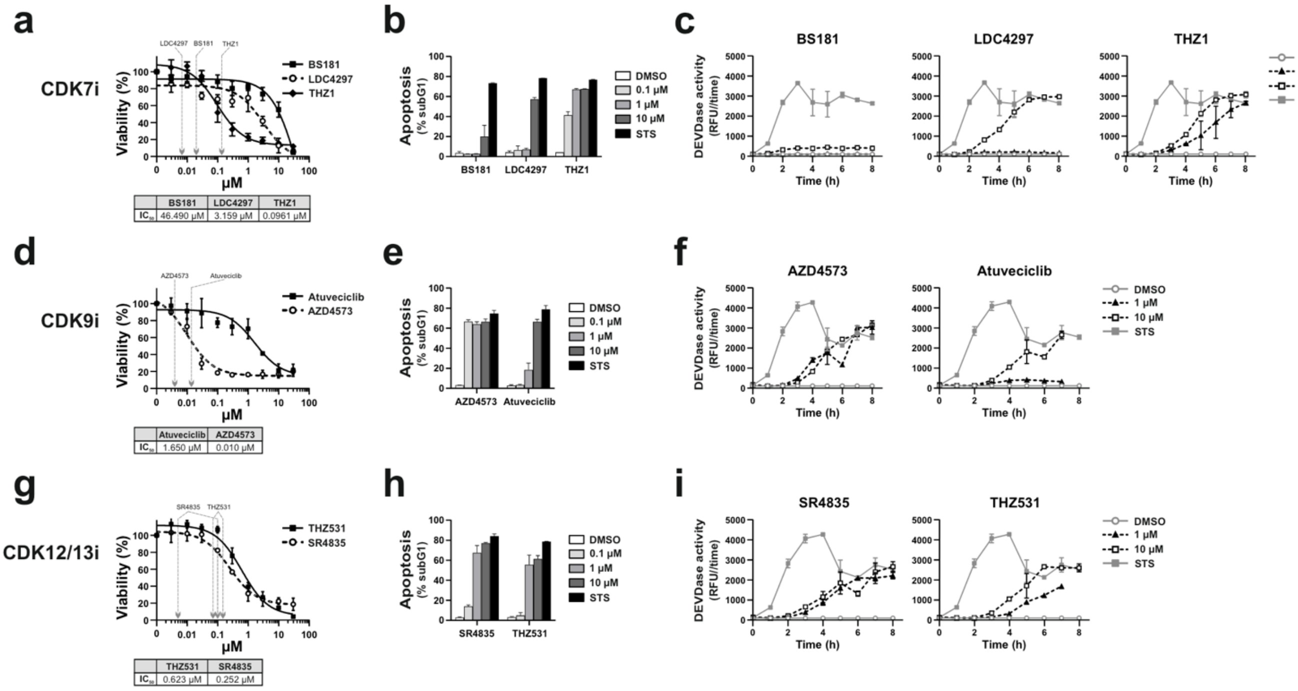

Supplementary Fig. S2: Inhibition of transcriptional elongation (CDK9 and CDK12/13), but not of transcriptional initiation (CDK7) induces cytotoxicity and apoptosis

(a,d,g) Cell viability of Jurkat cells treated with the indicated CDK inhibitors was measured by AlamarBlue® assay after 24 h. The cytotoxicity IC_{50} values are shown below each graph. The enzymatic IC_{50} values of CDK specific inhibitors are indicated by gray arrows (IC_{50} values of BS181, THZ531, SR4835, AZD4573, and atuveciclib were obtained from MedChemExpress (https://www.medchemexpress.com), and the IC_{50} values of THZ1 and LDC4297 from Lead Discovery Center GmbH; see Table 1). Error bars represent the mean ± SD of three independent experiments, performed in triplicate. **(b,e,h)** Jurkat cells were treated with 0.1, 1, or 10 μM of the indicated CDK inhibitors. DMSO (0.1%) served as diluent control, and staurosporine (STS, 2.5 μM) as a positive control for apoptosis induction. After 24 h, apoptosis was assessed by flow cytometric measurement of apoptotic hypodiploid nuclei. Error bars represent the mean ± SD of two independent experiments, performed in triplicate. **(c,f,i)** Jurkat cells were treated with 1, or 10 μM of the indicated CDK inhibitors, 0.1% DMSO, or 2.5 μM staurosporine (STS) for up to 8 hours. Caspase-3 activity was determined by measurement of the fluorescence of the profluorescent caspase-3 substrate DEVD-AMC in a spectrofluorometer. Error bars represent the mean ± SD from one experiment, performed in duplicate.

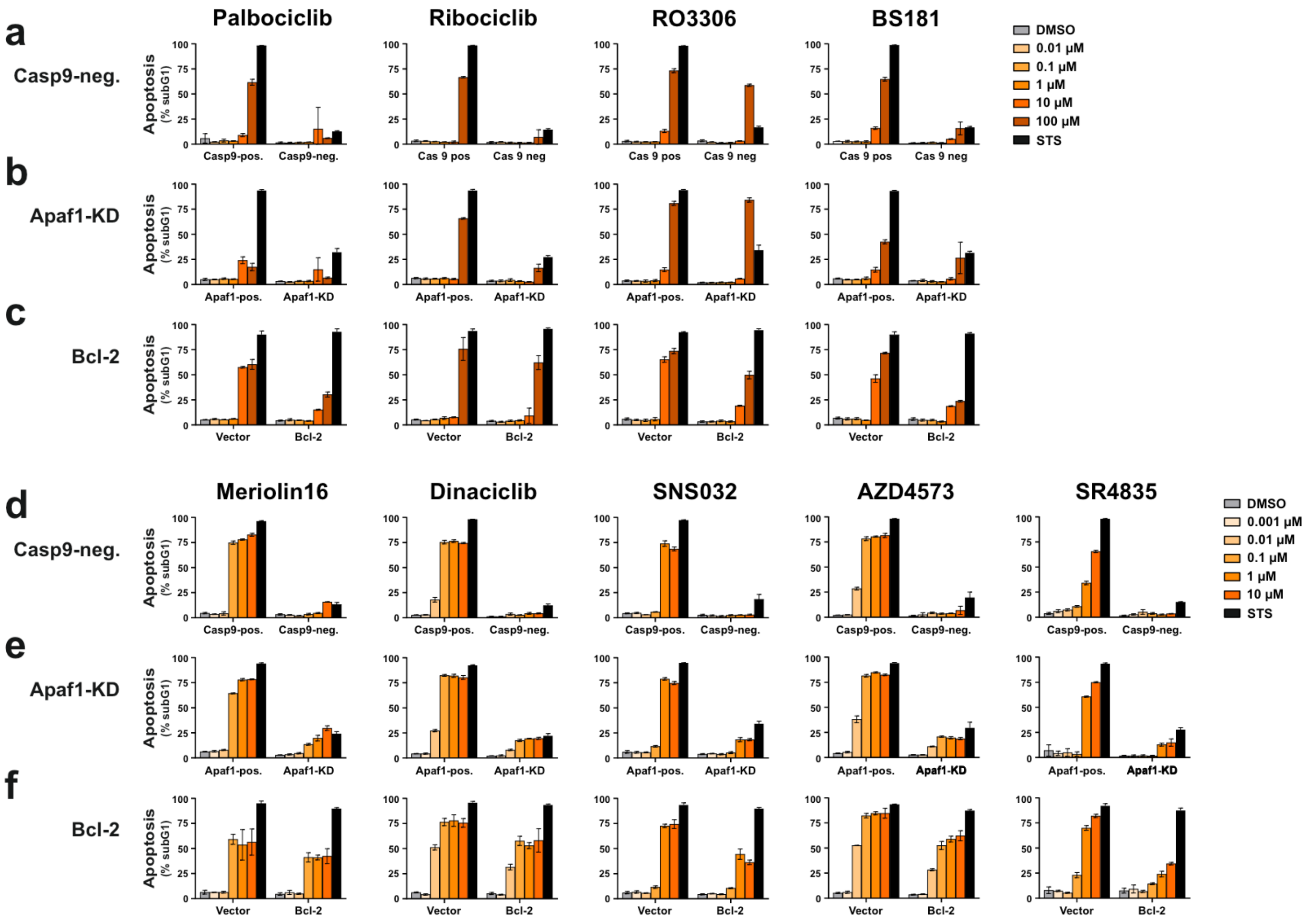

Supplementary Fig. S3: Apoptosis induced by pan-specific and transcriptional CDK inhibitors, but not by cell cycle-specific CDK inhibitors, is dependent on caspase-9 and Apaf1, but largely independent on Bcl-2

(a,d) Caspase-9 proficient (Casp9-pos.) or caspase-9 deficient Jurkat cells (Casp9-neg.), **(b,e)** Apaf-1 proficient (Apaf1-pos.) or Apaf-1 knockdown Jurkat cells (Apaf1-KD), or **(c,f)** Jurkat cells stably transfected with vectors encoding Bcl-2 (Bcl-2) or an empty vector (Vector) were treated at the indicated concentrations of the respective inhibitors (CDK4/6i: palbociclib and ribociclib; CDK1i: RO0336; CDK7i: BS181; pan-CDKi: meriolin16, dinaciclib, and SNS032; CDK9i: AZD4573; CDK12/13i: SR4835). DMSO (0.1%) was used as a diluent control, and 2.5 µM staurosporine (STS) as a positive control for apoptosis induction. After 24 h, apoptosis was assessed by flow cytometric measurement of apoptotic hypodiploid nuclei. **(a-f)** The results of the same experiments using a treatment of only 1 µM of the respective CDK inhibitors are shown in Fig. 2c-d. Error bars represent the mean ± SD from one experiment, performed in triplicate.

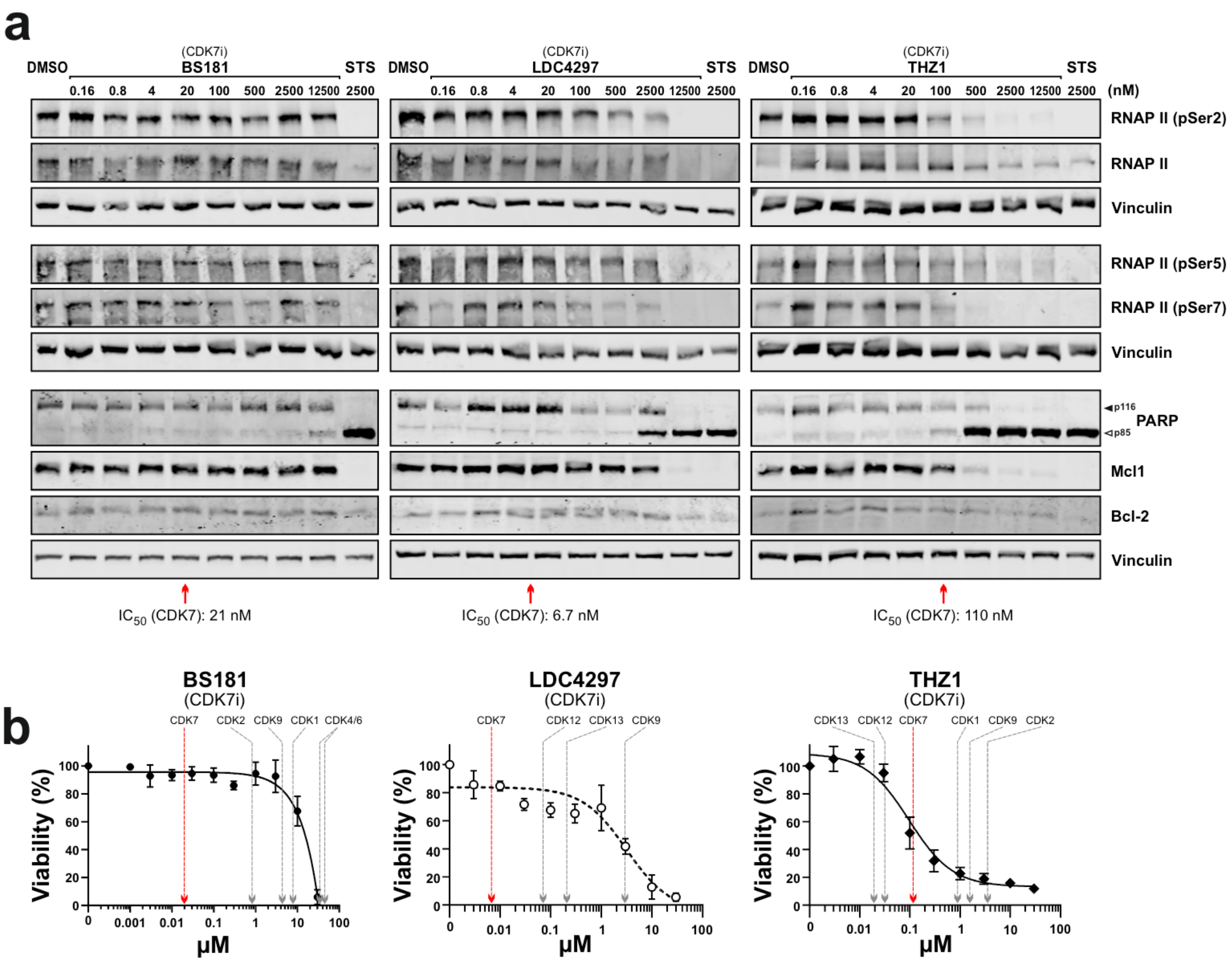

Supplementary Fig. S4: Effects of different CDK7 inhibitors on RNA polymerase II phosphorylation and Mcl1 expression in Jurkat cells

(a) Jurkat cells were treated with increasing concentrations of the CDK7 inhibitors BS181, LDC4297, or THZ1 for 6 h and then immunoblotted for: RNA polymerase II (RNAPII), phospho-Ser2 of the CTD of RNAPII (RNAPII pSer2), RNAPII Ser5, RNAPII pSer7, Mcl1, Bcl-2, vinculin (served as loading control), and cleavage of the caspase substrate PARP (solid arrowheads indicate the uncleaved form of PARP (p116); open arrowheads indicate the cleaved form (p85)). The enzymatic IC_{50} values of CDK specific inhibitors are indicated by red arrows (see Table 1). One representative immunoblot from two independent experiments is shown. **(b)** Viability assays of Jurkat cells that have been treated with increasing concentrations of the CDK7 inhibitors BS181, LDC4297, or THZ1 for 24 h. Arrows indicate the enzymatic IC_{50} values of targeted CDKs by respective CDK inhibitors (IC_{50} value of BS181 was obtained from MedChemExpress (https://www.medchemexpress.com), and the IC_{50} values of LDC4297 and THZ1 from Lead Discovery Center GmbH; see Table 1). The viability curve of BS181 was reused from Fig. 2a. Error bars represent the mean ± SD of three independent experiments, performed in triplicate.

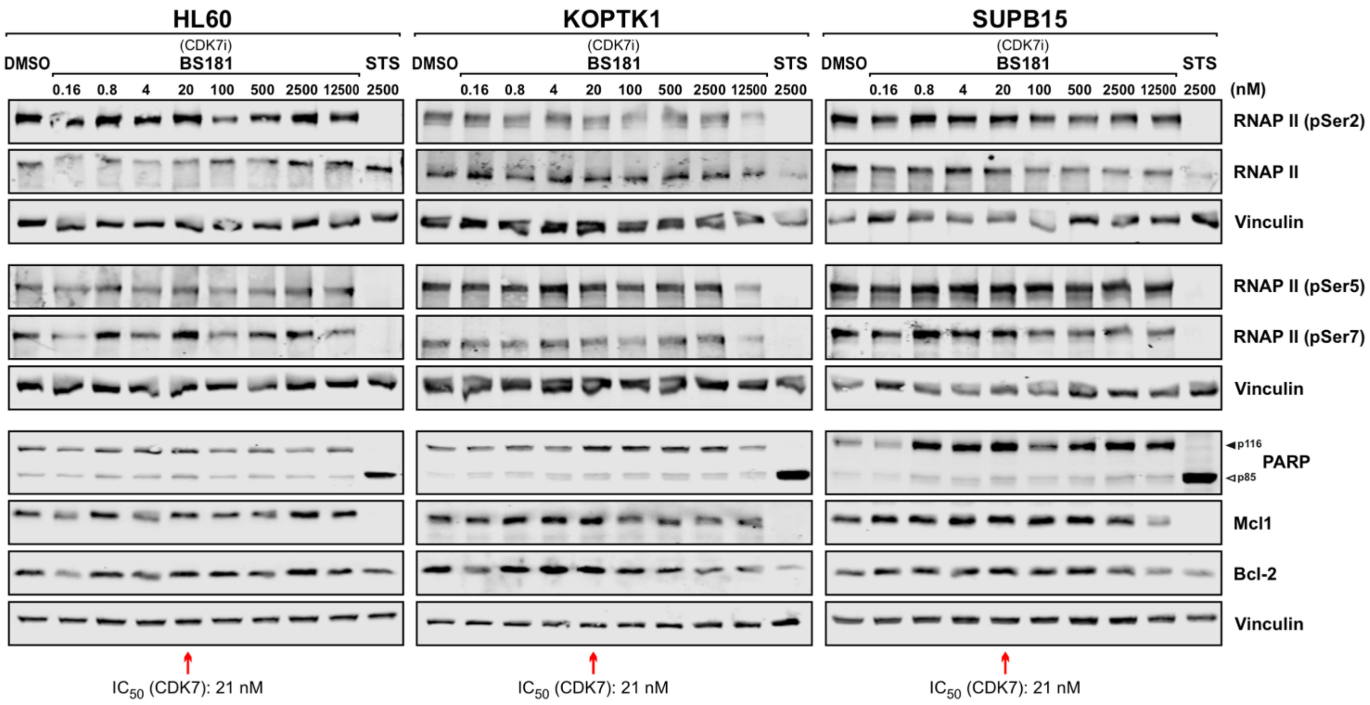

Supplementary Fig. S5: CDK7 inhibition by BS181 does not affect RNA polymerase II phosphorylation or Mcl1 expression in different leukemia cell lines

The different human leukemia cell lines HL60 (AML), KOPTK1 (T-ALL), and SUPB15 (B-ALL), were treated with increasing concentrations of the CDK7 inhibitor BS181 for 6 h and then immunoblotted for: RNA polymerase II (RNAPII), phospho-Ser2 of the CTD of RNAPII (RNAPII pSer2), RNAPII Ser5, RNAPII pSer7, Mcl1, Bcl-2, vinculin (served as loading control), and cleavage of the caspase substrate PARP (solid arrowheads indicate the uncleaved form of PARP (p116); open arrowheads indicate the cleaved form (p85)). The enzymatic IC_{50} value of BS181 (obtained from MedChemExpress (https://www.medchemexpress.com); see Table 1) is indicated by the red arrow. One representative immunoblot from two independent experiments is shown.

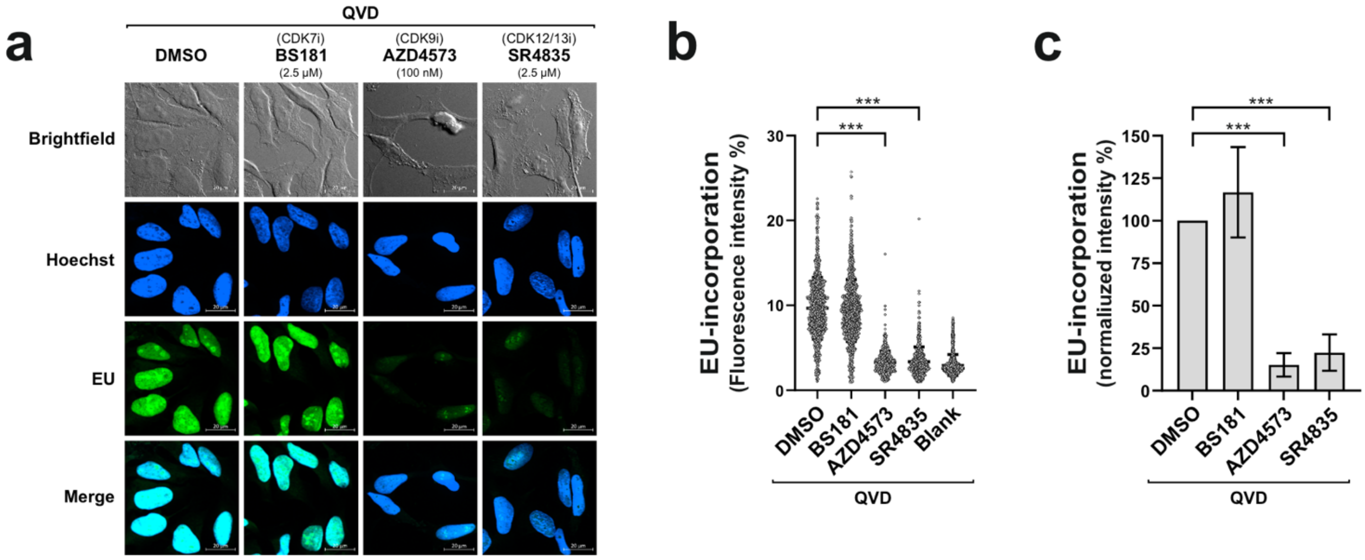

Supplementary Fig. S6: Transcriptional inhibition by CDK9 and CDK12/13 inhibitors is independent of caspase activity in HeLa cells

Experiments were performed as in Fig. 3c-d with the addition of the pan-caspase inhibitor Q-VD-OPh (QVD). **(a)** HeLa cells were pre-incubated with 10 μM QVD for 30 min, and subsequently treated with 2.5 μM of the CDK7 inhibitor BS181, 100 nM of the CDK9 inhibitor AZD4573, 2.5 μM of the CDK12/13 inhibitor SR4835, or 0.1% DMSO for 24 h. EU-incorporation was determined by microscopy, and exemplary images are shown (green: EU-incorporation; blue: DAPI stained nuclei). **(b)** Quantification of EU fluorescence intensity without normalization, with each point representing a single cell. **(c)** Summary quantification of EU incorporation from three independent experiments (two technical replicates, five images per replicate, ≥2175 cells were analyzed per condition). Statistical significance: *p<0.05, **p<0.01, ***p<0.001.

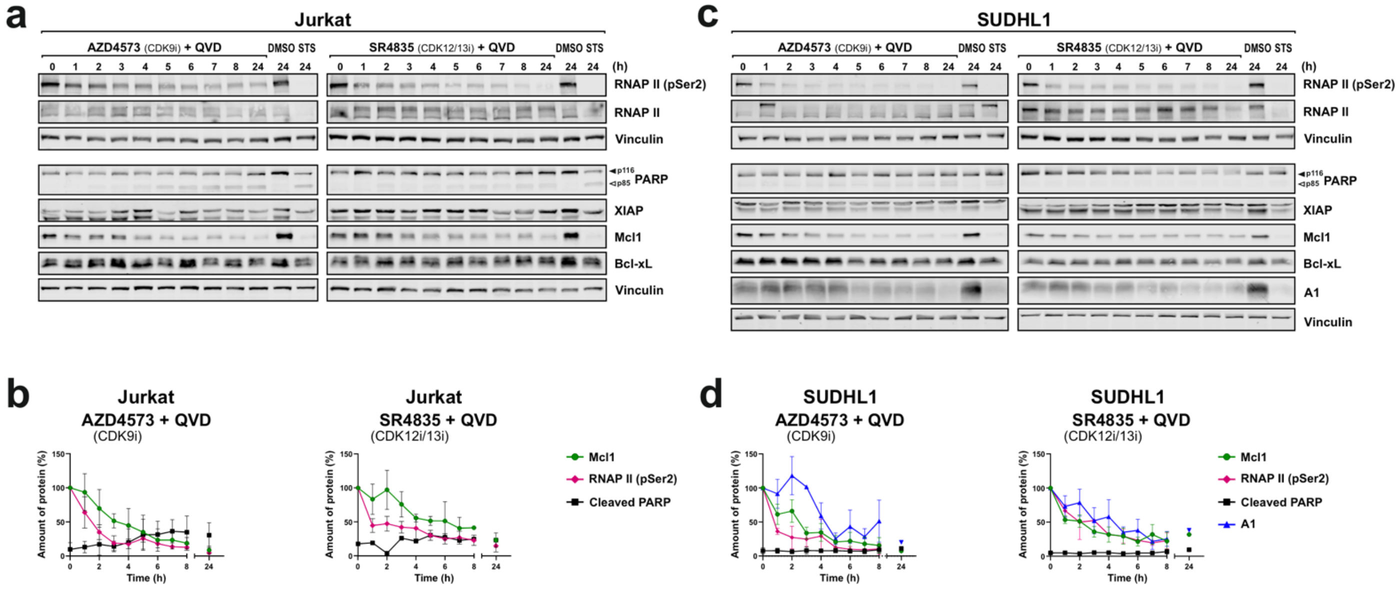

Supplementary Fig. S7: CDK9 and CDK12/13 inhibitor-induced reduction of Mcl1, A1, and RNA polymerase II phosphorylation occurs independently of caspase activity in Jurkat and SUDHL1 cells

Monitoring of RNAPII CTD dephosphorylation and protein degradation in Jurkat and SUDHL1 cells was performed as in Fig. 4c-f, with the addition of the pan-caspase inhibitor QVD. **(a,b)** Jurkat cells, or **(c,d)** SUDHL1 cells, were pre-incubated with 10 µM QVD for 30 min, and subsequently treated with 0.1 µM AZD4573 (CDK9i), or 2.5 µM SR4835 (CDK12/13i) for up to 24 h. DMSO (0.1%) was used as a diluent control, and 2.5 µM staurosporine (STS) as a positive control for apoptosis induction. Protein expression was monitored by immunoblotting for RNA polymerase II (RNAPII), phospho-Ser2 of the CTD of RNAPII (RNAPII pSer2), XIAP, Mcl1, Bcl-xL, vinculin (loading control), and cleavage of the caspase substrate PARP (solid arrowheads indicate the uncleaved form of PARP (p116); open arrowheads indicate the cleaved form (p85)). **(b,d)** Quantification of the immunoblot kinetics of Jurkat cells from (a) and SUDHL1 cells from (b). Protein expression of Mcl1, A1, RNAPII (pSer2) was normalized to vinculin. Cleaved PARP (p85) was normalized to the total amount of PARP (p85 + p116). **(a,c)** One representative immunoblot from three independent experiments is shown. **(b,d)** Error bars represent the mean ± SD from three independent experiments.

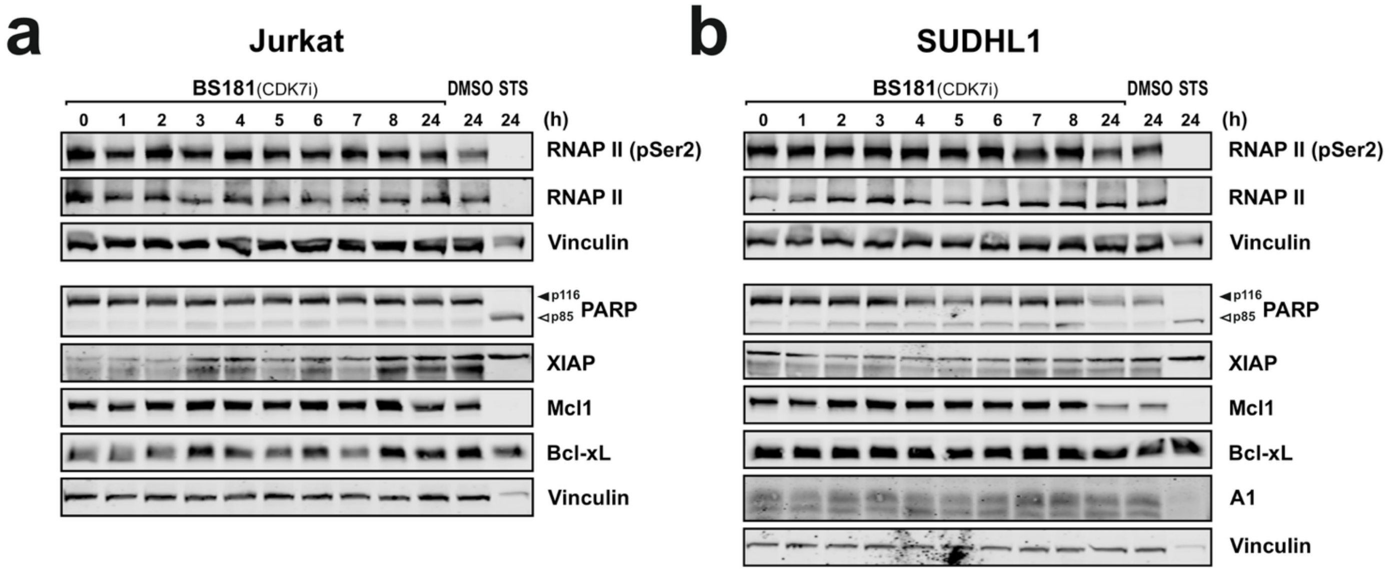

Supplementary Fig. S8: Inhibition of CDK7 has no effect on the downregulation of the anti-apoptotic Bcl-2 proteins Mcl1 and A1/Bfl1

Jurkat and SUDHL1 cells were treated with 2.5 µM BS181 (CDK7i) for up to 24 h. DMSO (0.1%) was used as a diluent control, and 2.5 µM staurosporine (2.5 µM; STS) as a positive control for apoptosis induction. Protein expression was monitored by immunoblotting for RNA polymerase II (RNAPII), phospho-Ser2 of the CTD of RNAPII (RNAPII pSer2), XIAP, Mcl1, Bcl-xL, A1, vinculin (loading control), and cleavage of the caspase substrate PARP (solid arrowheads indicate the uncleaved form of PARP (p116); open arrowheads indicate the cleaved form (p85)).

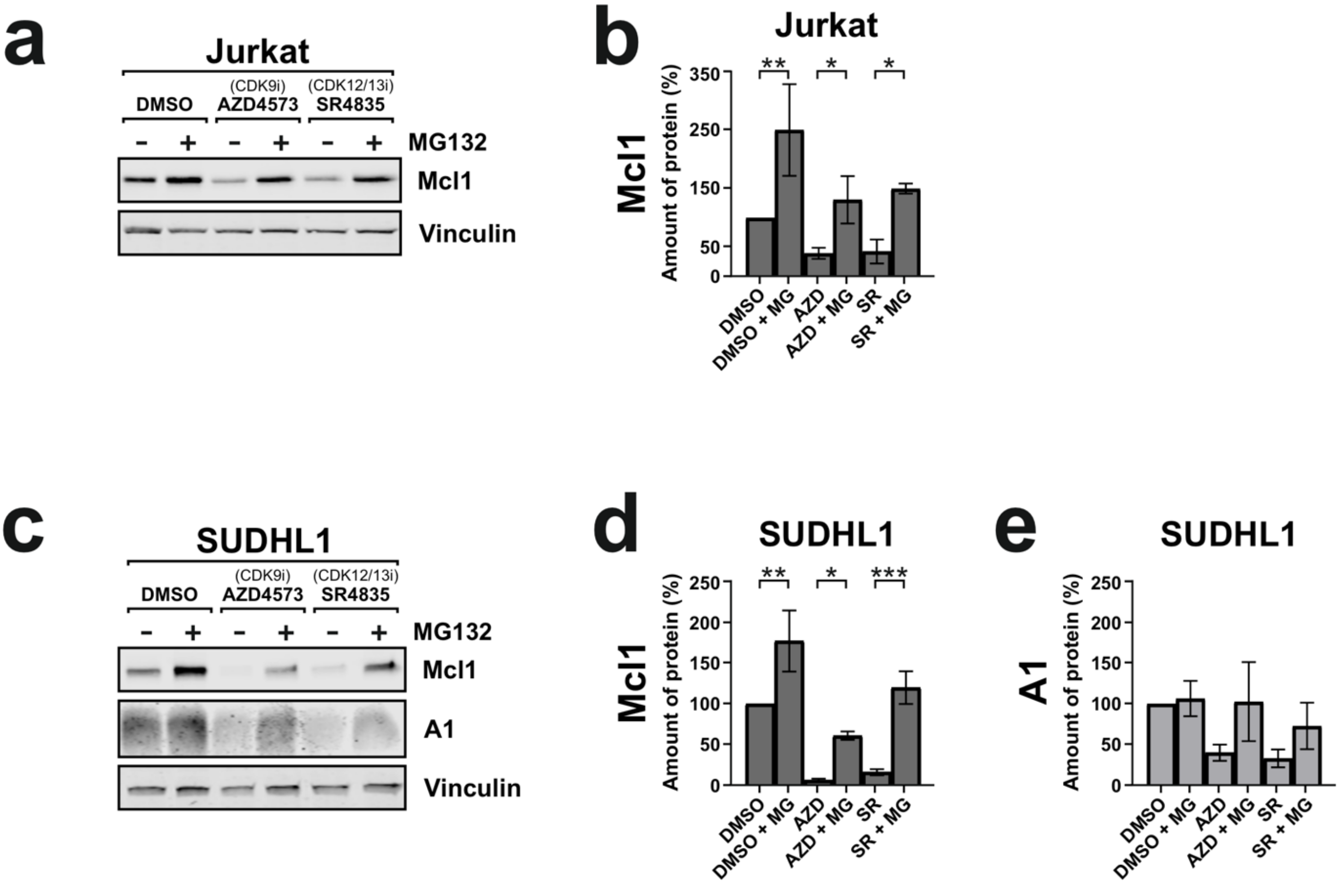

Supplementary Fig. S9: Proteasome inhibition by MG132 prevents the reduction of short-lived proteins Mcl1 and A1 induced by CDK9 and CDK12/13 inhibitors

(a) Jurkat or **(c)** SUDHL1 cells were pre-incubated for 30 min with the proteasome inhibitor MG132 (10 µM) in the presence of the pan-caspase inhibitor QVD (10 µM). Subsequently, cells were treated with either 0.1 µM AZD4573 (CDK9i) or 2.5 µM SR4835 (CDK12/13i) for 6 h, and immunoblotted for Mcl1, A1, and vinculin (loading control). **(b)** Quantification of Mcl1 protein levels from the immunoblot in panel A. Quantification of **(d)** Mcl1 or **(e)** A1 protein levels from the immunoblot in panel C. **(a,c)** One representative immunoblot from three independent experiments is shown. **(b-e)** Error bars represent the mean ± SD from three independent experiments. Statistical significance: *$p<0.05$, **$p<0.01$, ***$p<0.001$.

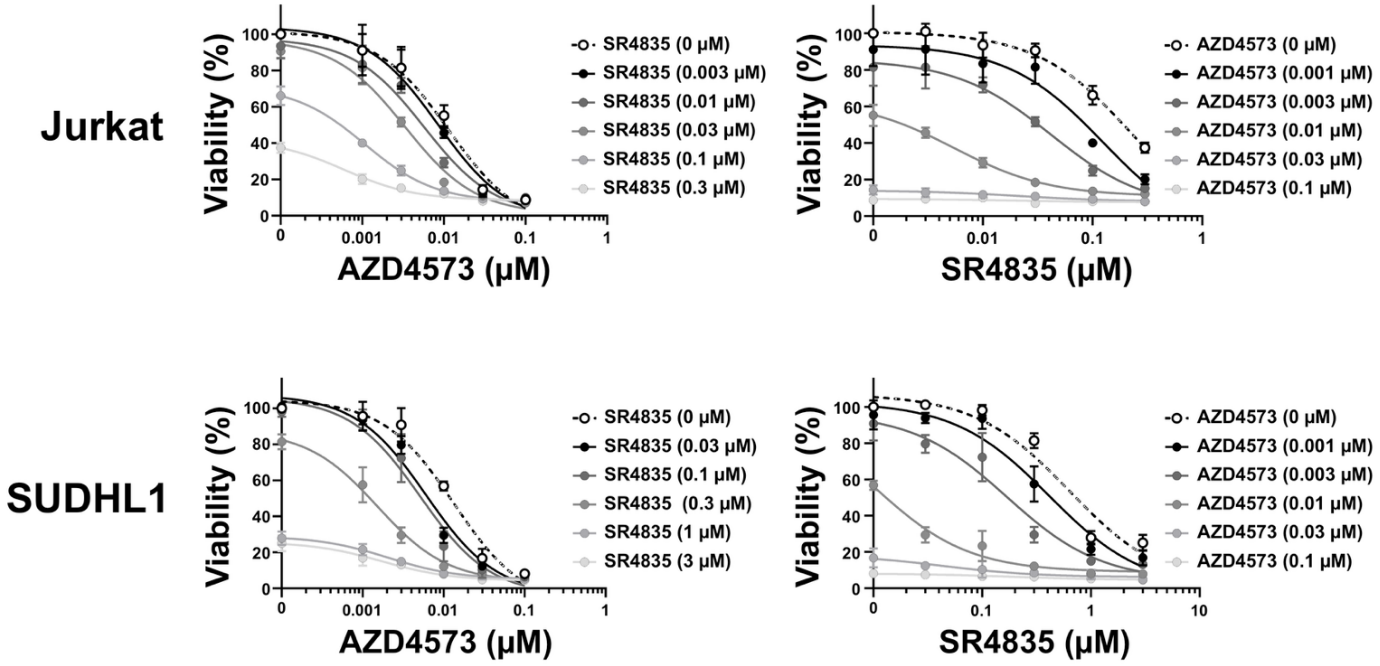

Supplementary Fig. S10: Inhibitors of CDK9 and CDK12/13 act synergistically in cell death induction

Jurkat or SUDHL1 cells were treated with AZD4573 (CDK9i) in combination with SR4835 (CDK12/13i). After 24 h, cell viability was assessed by AlamarBlue® assay. Drug concentrations were chosen to cover the full dynamic range of viability responses (from complete survival to complete cell death), to ensure reliable synergy evaluation. Viability curves for the co-treatments used to determine IC_{50} values for the isobologram analysis are shown in Fig. 5f. These data are derived from the same experimental dataset used for the synergy analysis presented in Fig. 5d. For Jurkat cells, the appropriate synergy concentration range is 0.003 - 0.03 µM for SR4835 and 0.001 - 0.1 µM for AZD4573. For SUDHL1 cells, it is 0.03 - 0.3 µM for SR4835 and 0.001 - 0.1 µM for AZD4573. Results of three independent experiments, performed in duplicate, are shown.

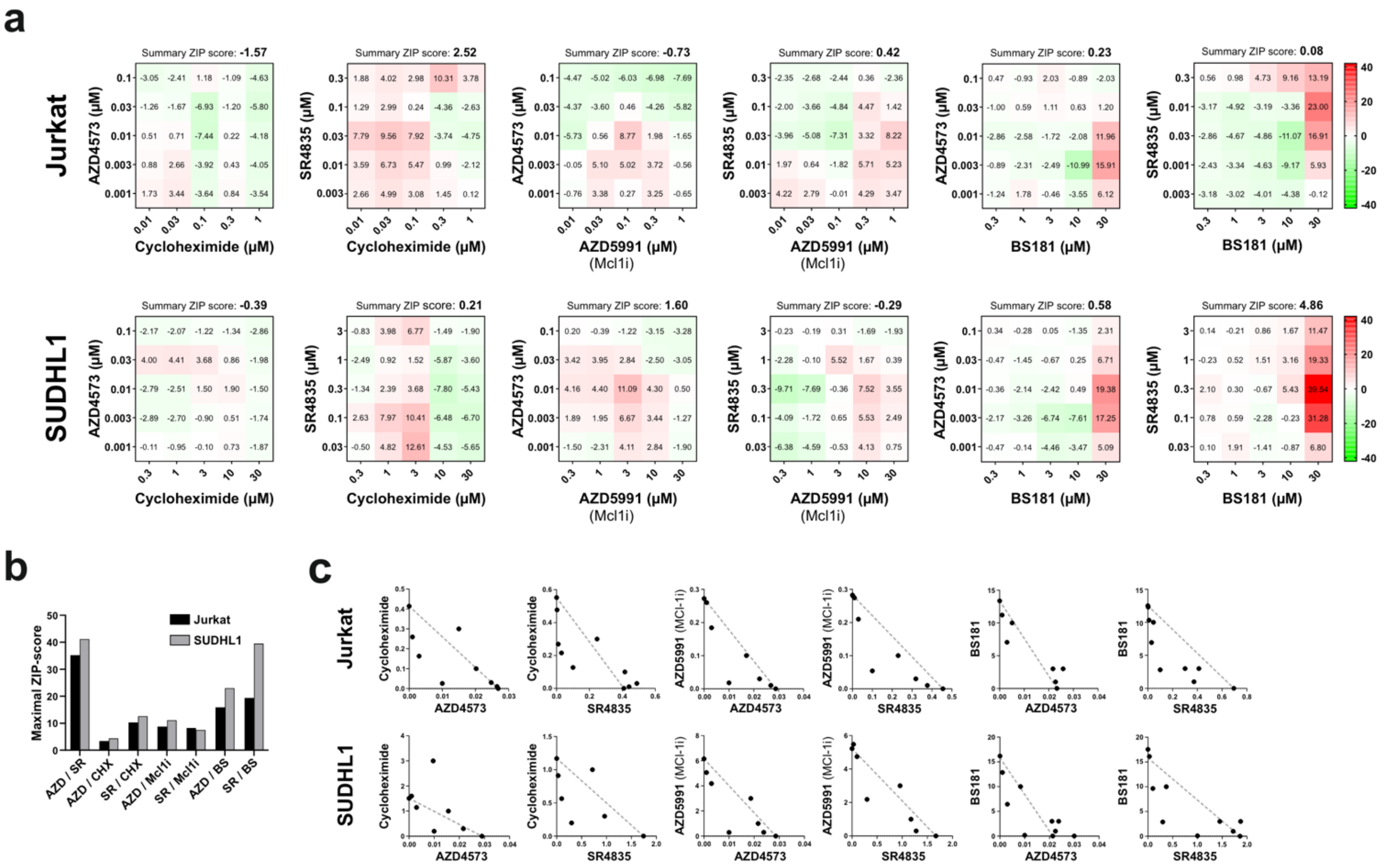

Supplementary Fig. S11: Combinatorial treatment of AZD4573 or SR4835 with the translational inhibitor cycloheximide, the Mcl1 inhibitor AZD5991, or BS181 (CDK7i) in Jurkat and SUDHL1 cells

(a) Jurkat or SUDHL1 cells were treated with either AZD4573 (CDK9i) or SR4835 (CDK12/13i), in combination with the translation inhibitor cycloheximide, the Mcl1 inhibitor AZD5991, or the CDK7 inhibitor BS181. After 24 h, cell viability was assessed by AlamarBlue® assay. Drug concentrations were chosen to cover the full dynamic range of viability responses (from complete survival to complete cell death), to ensure reliable synergy evaluation. Subsequently, SynergyFinder was used to perform a synergy analysis. All summary ZIP scores range between -2 and 5, indicating no significant synergy. **(b)** Maximum ZIP scores of the same combinations, analogous to Fig. 5e. All treatments show maximal ZIP scores around 10 (with exception of BS181), substantially lower than the synergy observed for AZD4573/SR4835. **(c)** Isobologram analysis of the co-treatments, analogous to Fig. 5f, confirms the absence of synergistic effects (with exception of BS181). Note that the high ZIP scores (a, b) and synergistic effects (c) of BS181 cotreatment are only observed at high concentrations of BS181, where other CDKs are also affected (such as CDK9; see Table 1). Results of three independent experiments, performed in duplicate, are shown.

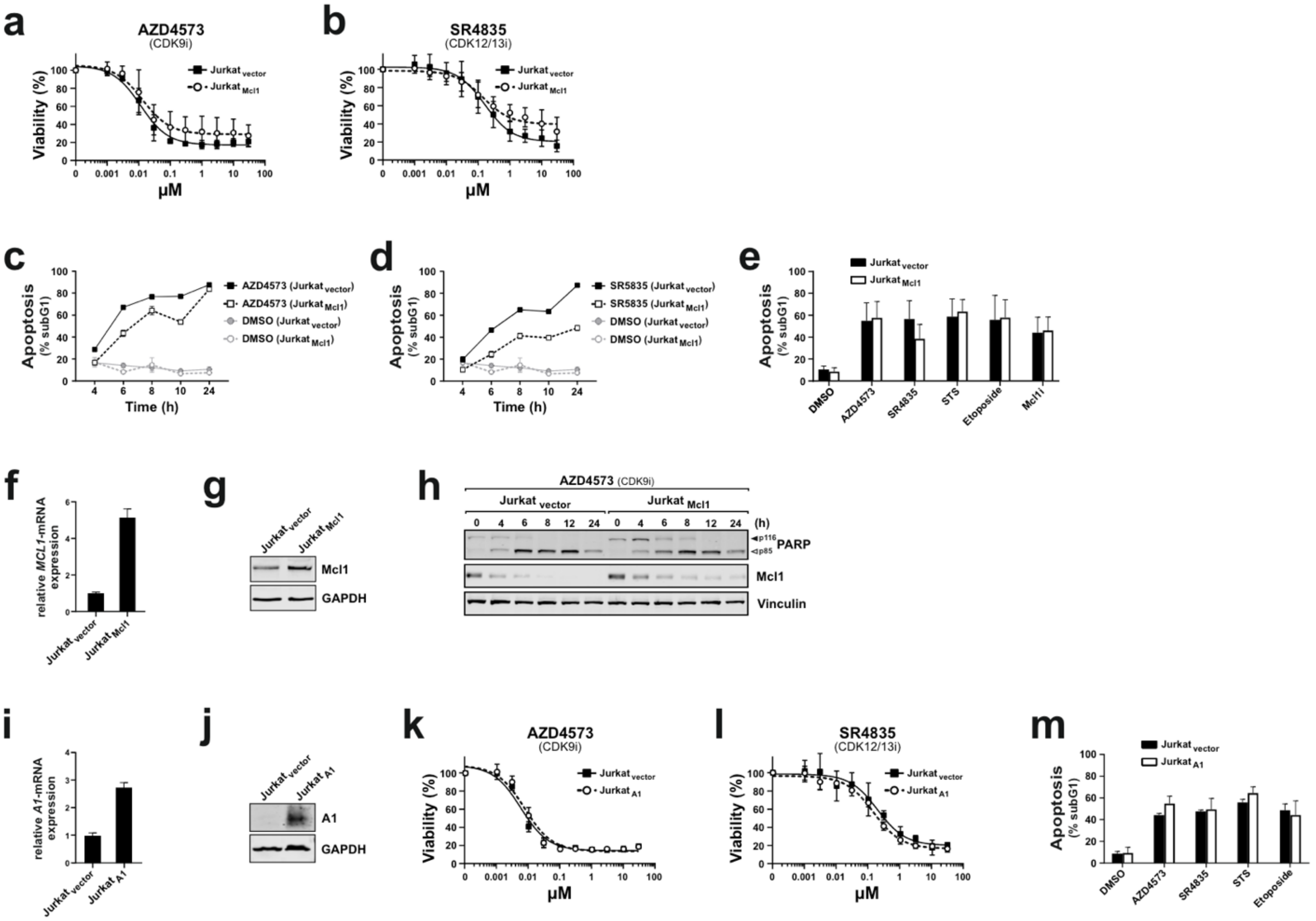

Supplementary Fig. S12: Effect of Mcl1 and A1 overexpression on CDK9 and CDK12/13 inhibitor-induced cytotoxicity and apoptosis in Jurkat cells

(a) Jurkat cells overexpressing Mcl1 (Jurkat Mcl1) or empty vector (Jurkat vector) were treated with 0.1 µM AZD4573 (CDK9i), or **(b)** with 2.5 µM SR4835 (CDK12/13i) for 24 h. Subsequently, cell viability was assessed by AlamarBlue® assay. Error bars represent the mean ± SD of three independent experiments, performed in triplicate. Jurkat Mcl1 or Jurkat vector cells were treated with **(c)** 0.1 µM AZD4573 (CDK9i), or **(d)** 2.5 µM SR4835 (CDK12/13i) for up to 24 h. Subsequently, apoptosis was assessed by flow cytometric measurement of apoptotic hypodiploid nuclei. Measurements were performed in triplicate. **(e)** Jurkat Mcl1 or Jurkat vector cells were treated with 0.1 µM AZD4573 (CDK9i), 2.5 µM SR4835 (CDK12/13i), 2.5 µM staurosporine, 50 µM etoposide, or 2.5 µM of the Mcl1-inhibitor AZD5991. After 24 h, apoptosis was assessed by flow cytometric measurement of apoptotic hypodiploid nuclei. Error bars represent the mean ± SD of three independent experiments, performed in triplicate. **(f)** qPCR analysis of basal Mcl1 mRNA levels in untreated Jurkat Mcl1 or Jurkat vector cells shows ~5-fold higher expression in Mcl1-overexpressing cells. Error bars represent relative quantity (mean ± SEM) from one representative experiment, performed in triplicate. **(g)** Immunoblot of Mcl1 protein expression in Jurkat Mcl1 or Jurkat vector cells. GAPDH was used as loading control. **(h)** Jurkat Mcl1 or Jurkat vector cells were treated with 0.1 µM AZD4573 (CDK9i) for up to 24 h and then immunoblotted for Mcl1 and the cleavage of the caspase substrate PARP (solid arrowheads indicate the uncleaved form of PARP (p116); open arrowheads indicate the cleaved form (p85)). Vinculin served as a loading control. **(i)** qPCR analysis of basal A1 mRNA levels in untreated Jurkat A1 or Jurkat vector cells shows ~2.5-fold higher expression in A1-overexpressing cells. Bars represent relative quantity (mean ± SEM) from one

representative experiment, performed in triplicate. **(j)** Immunoblot of A1 protein expression in Jurkat A1 or Jurkat vector cells. GAPDH was used as loading control. **(k)** Jurkat cells overexpressing A1 (Jurkat A1) or empty vector (Jurkat vector) were treated with 0.1 µM AZD4573 (CDK9i), or **(l)** with 2.5 µM SR4835 (CDK12/13i) for 24 h. Subsequently, cell viability was assessed by AlamarBlue® assay. Error bars represent the mean ± SD of three independent experiments, performed in triplicate. **(m)** Jurkat A1 or Jurkat vector cells were treated with 0.1 µM AZD4573 (CDK9i), 2.5 µM SR4835 (CDK12/13i), 2.5 µM staurosporine (STS) or 50 µM etoposide. After 24 h, apoptosis was assessed by flow cytometric measurement of apoptotic hypodiploid nuclei. Error bars represent the mean ± SD of three independent experiments, performed in triplicate.
