# Supplementary material for: Inhibition of RNA polymerase II-activating CDK9 and CDK12/13, but not of cell cycle relevant CDKs, induces apoptosis by downregulating the short-lived Bcl-2 proteins Mcl1 and Bfl1/A1
Source: Cell Death Dis. 2026 May 27;17(1):512. doi: 10.1038/s41419-026-08889-6 (PMC13216340; doi:10.1038/s41419-026-08889-6)

Original Western Blots

(all western blots were scanned via LI-COR Odyssey® imaging system)

Figure 3a (left panel)

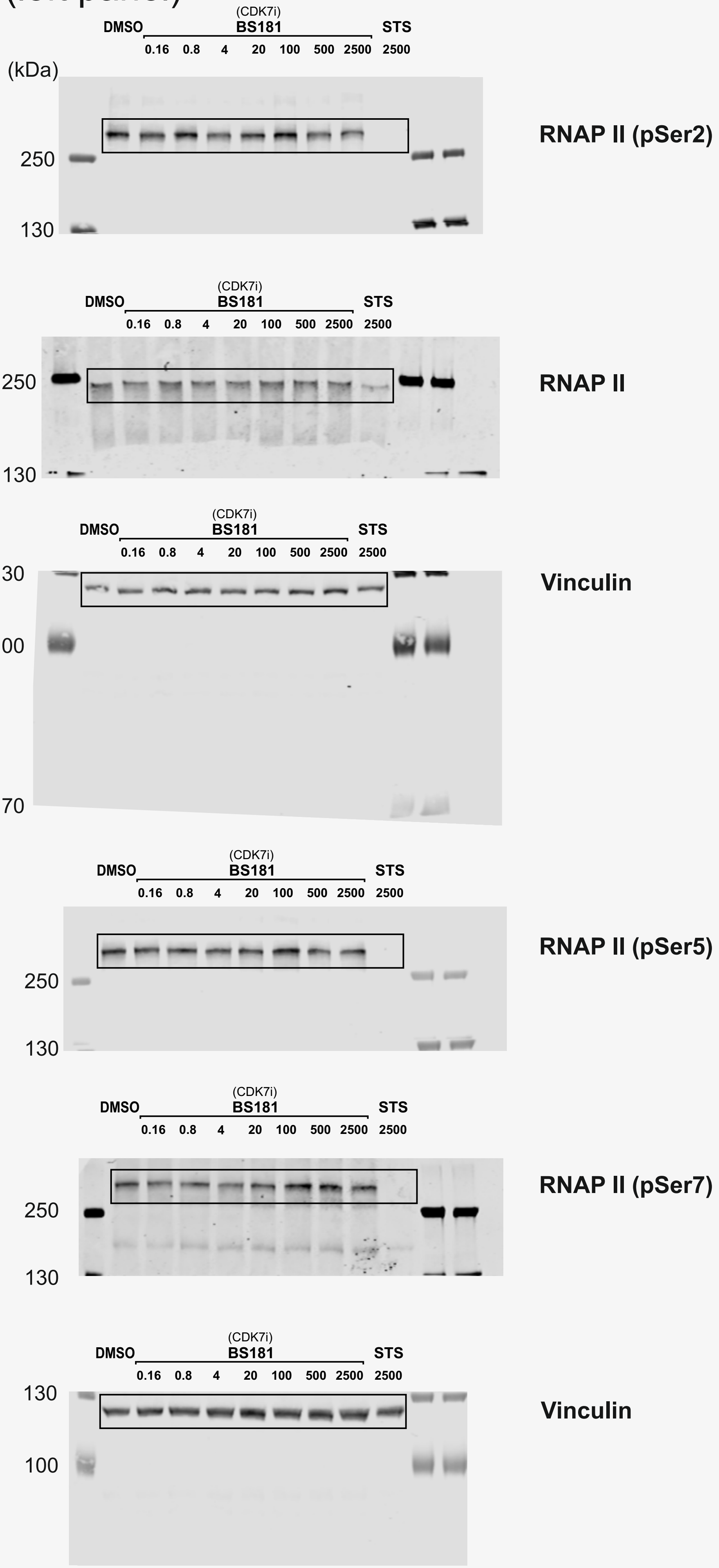

Figure 3a (left panel)

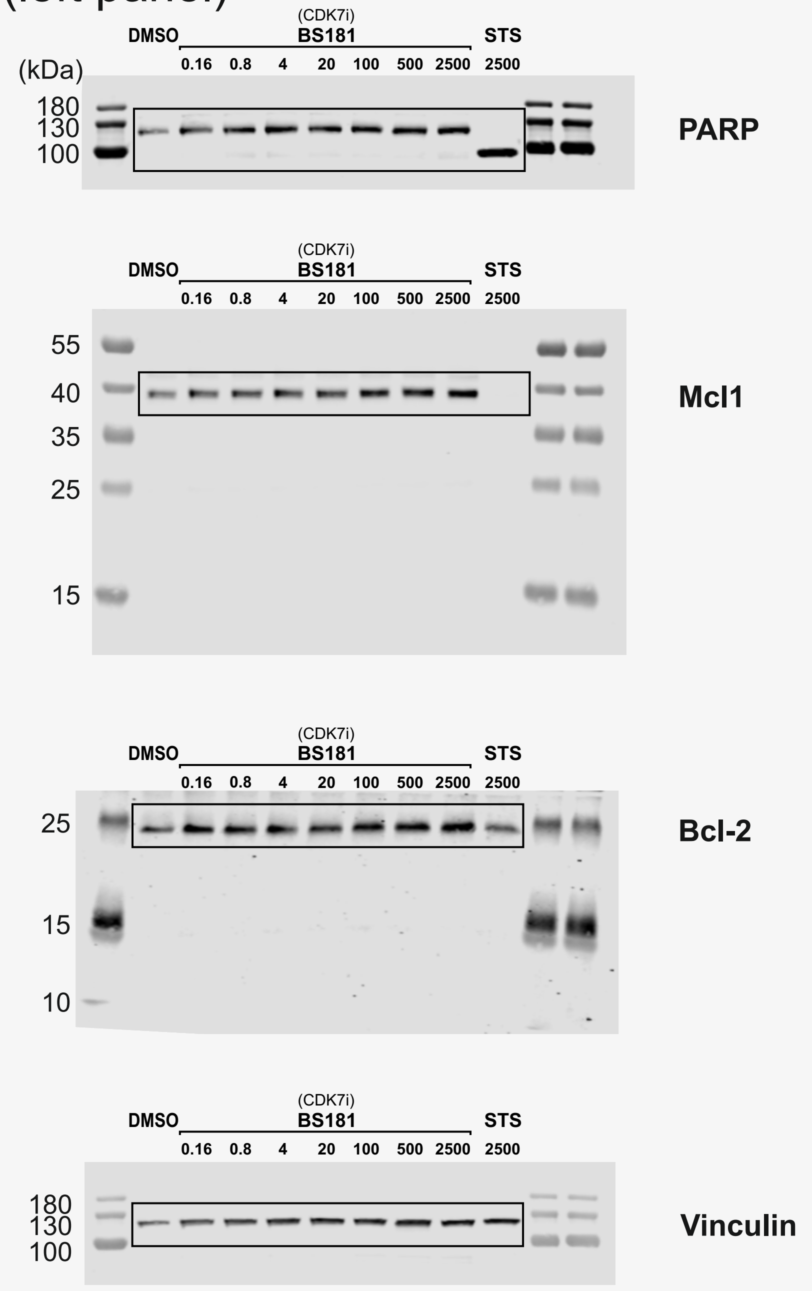

Figure 3a (middle panel)

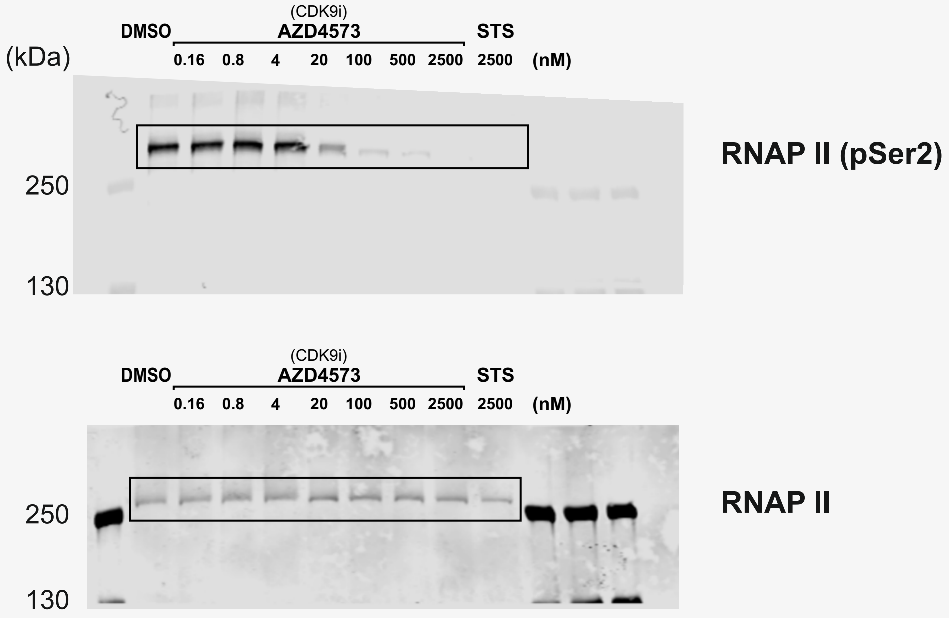

Figure 3a (middle panel)

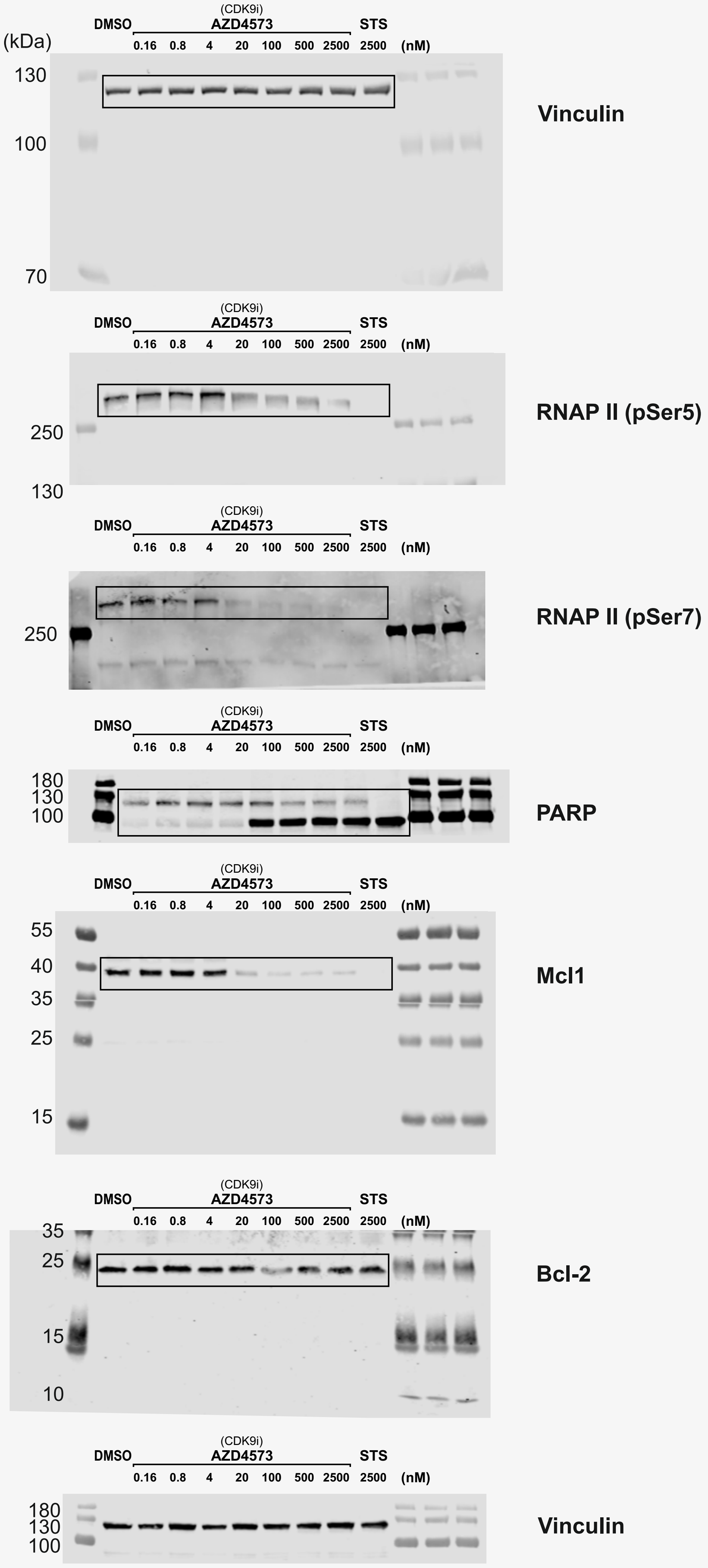

Figure 3a (right panel)

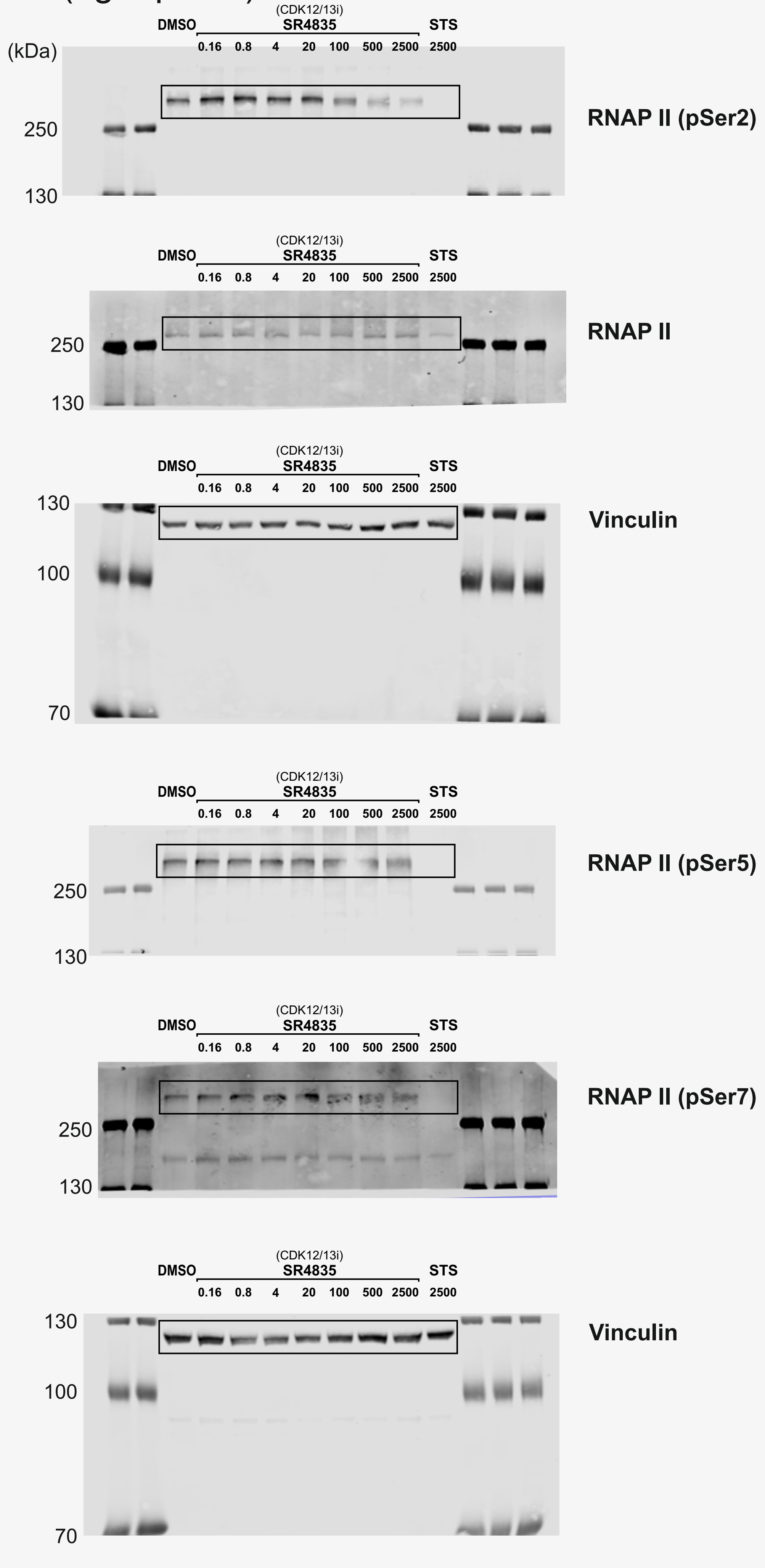

Figure 3a (right panel)

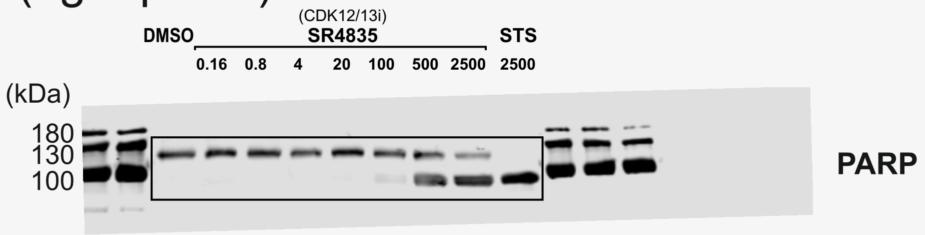

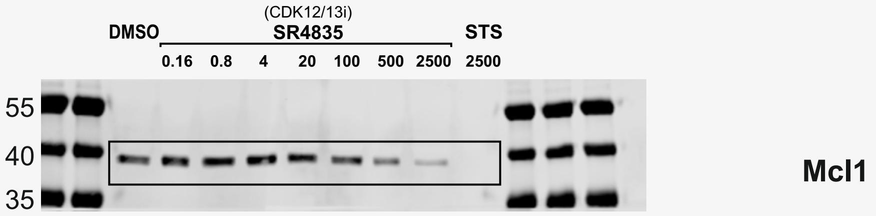

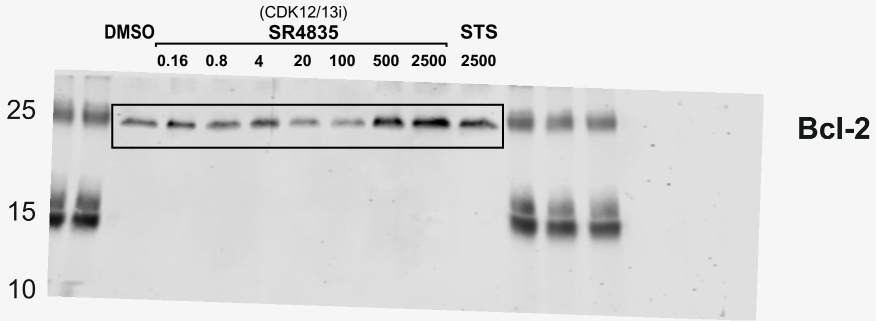

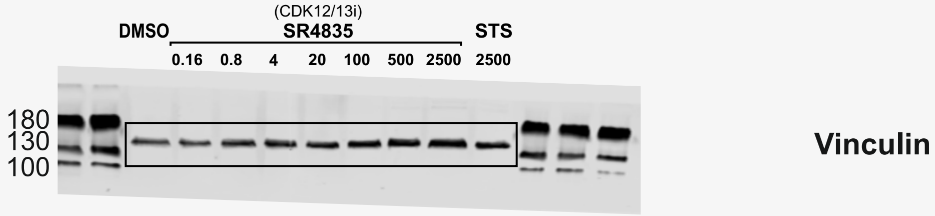

Figure 4a

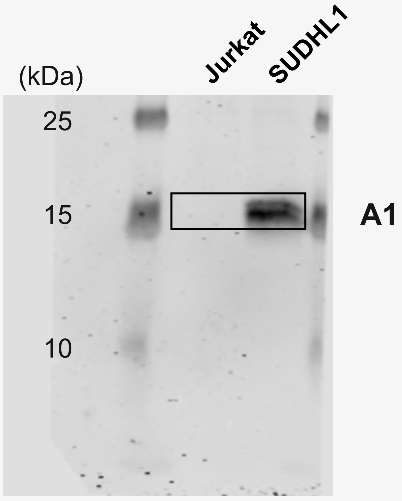

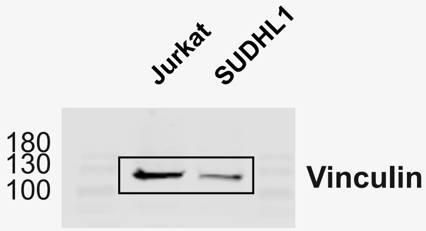

Figure 4a

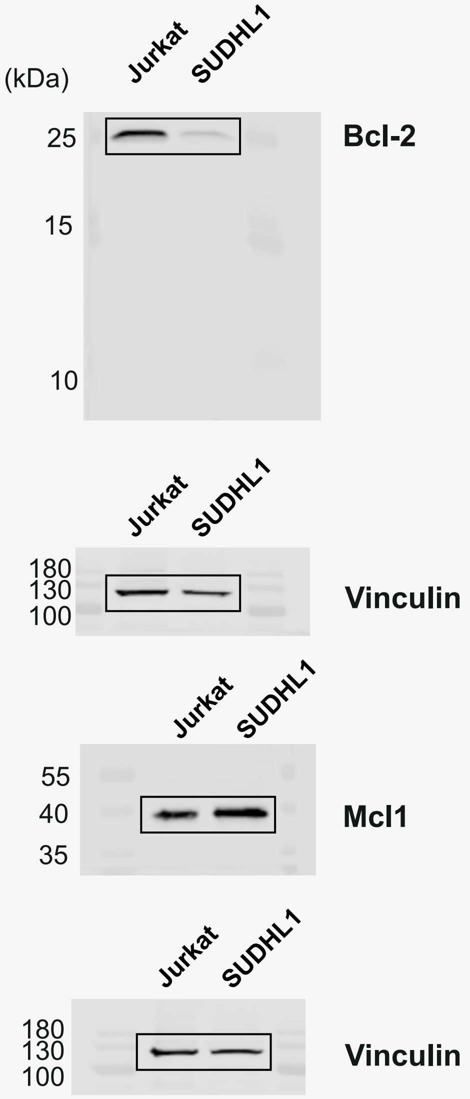

Figure 4c (left panel)

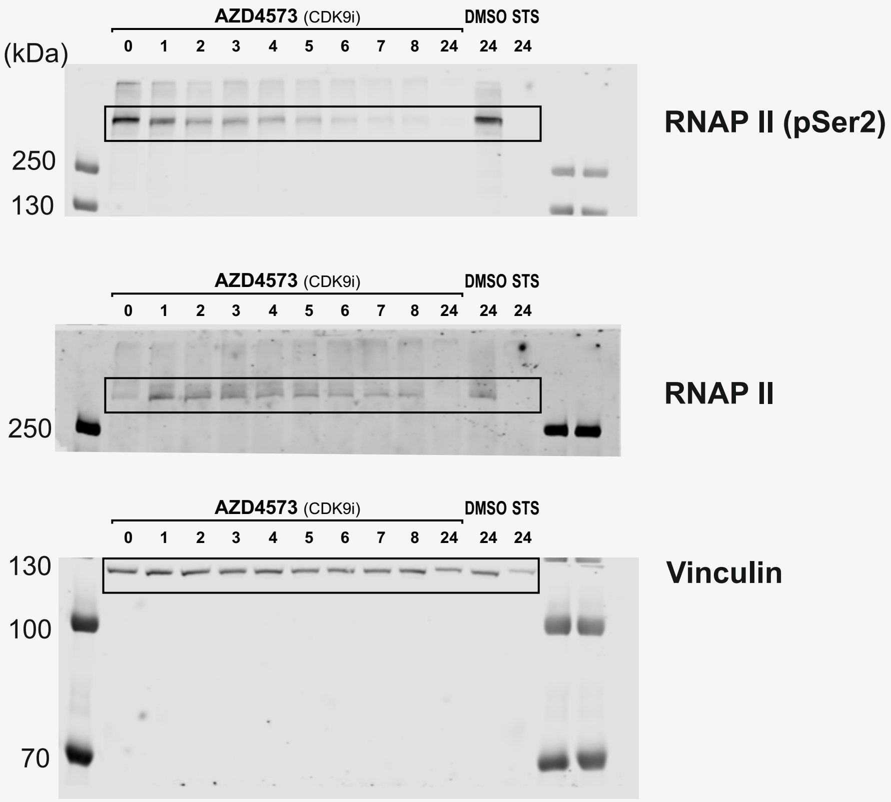

Figure 4c (left panel)

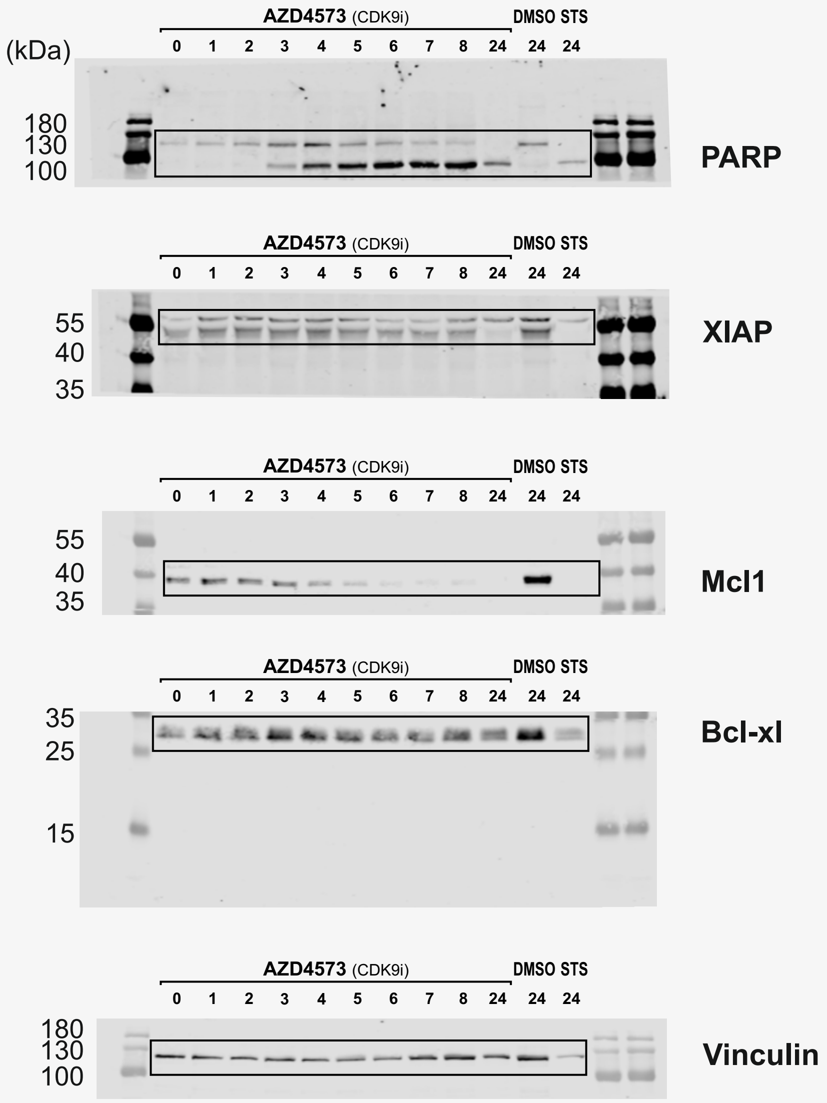

Figure 4c (right panel)

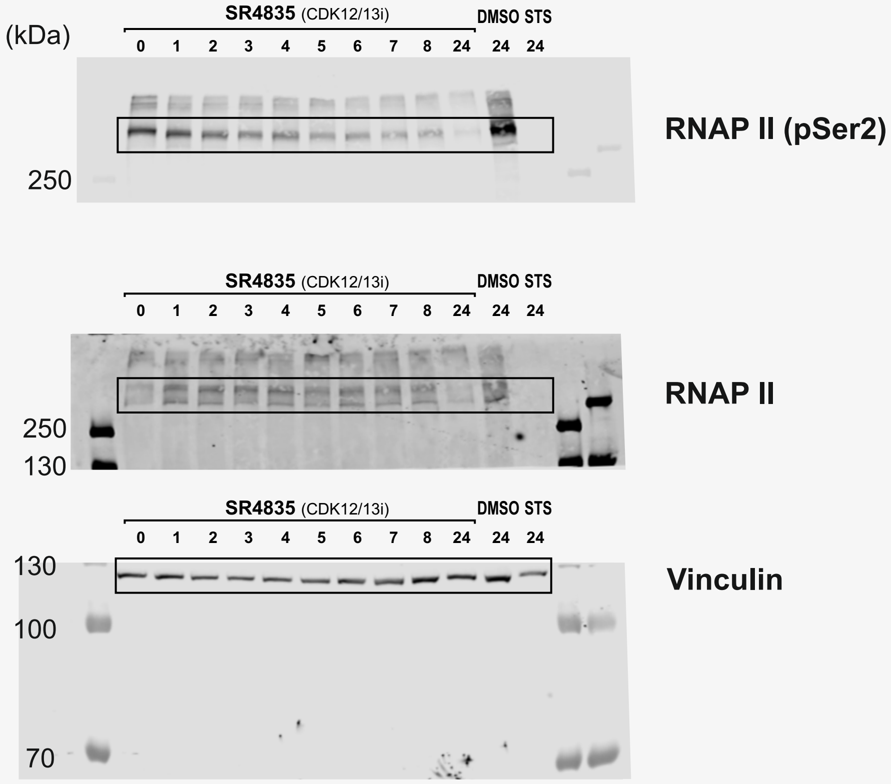

Figure 4c (right panel)

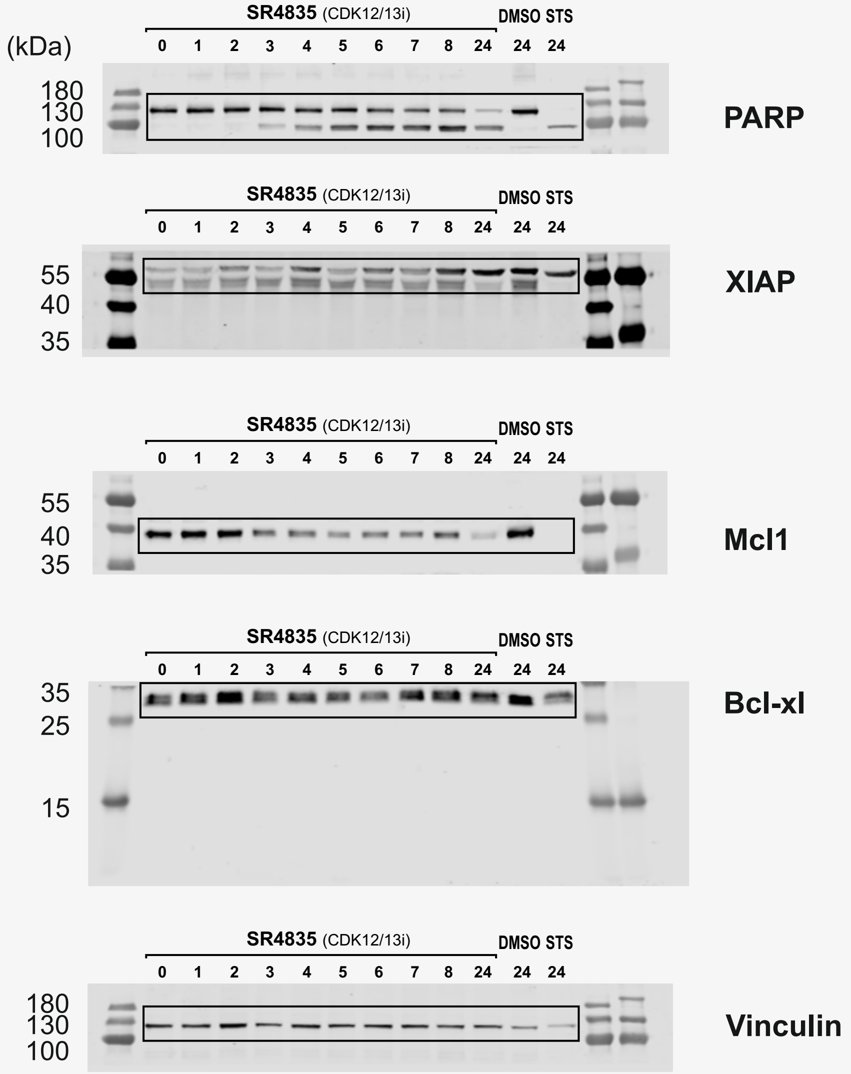

Figure 4e (left panel)

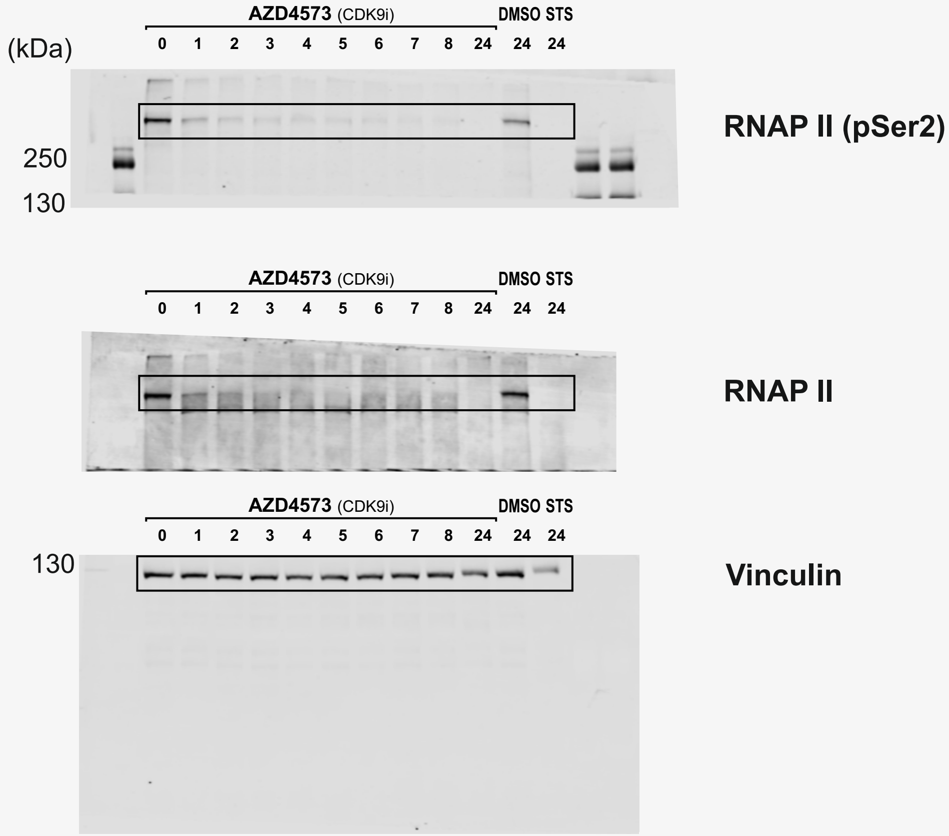

Figure 4e (left panel)

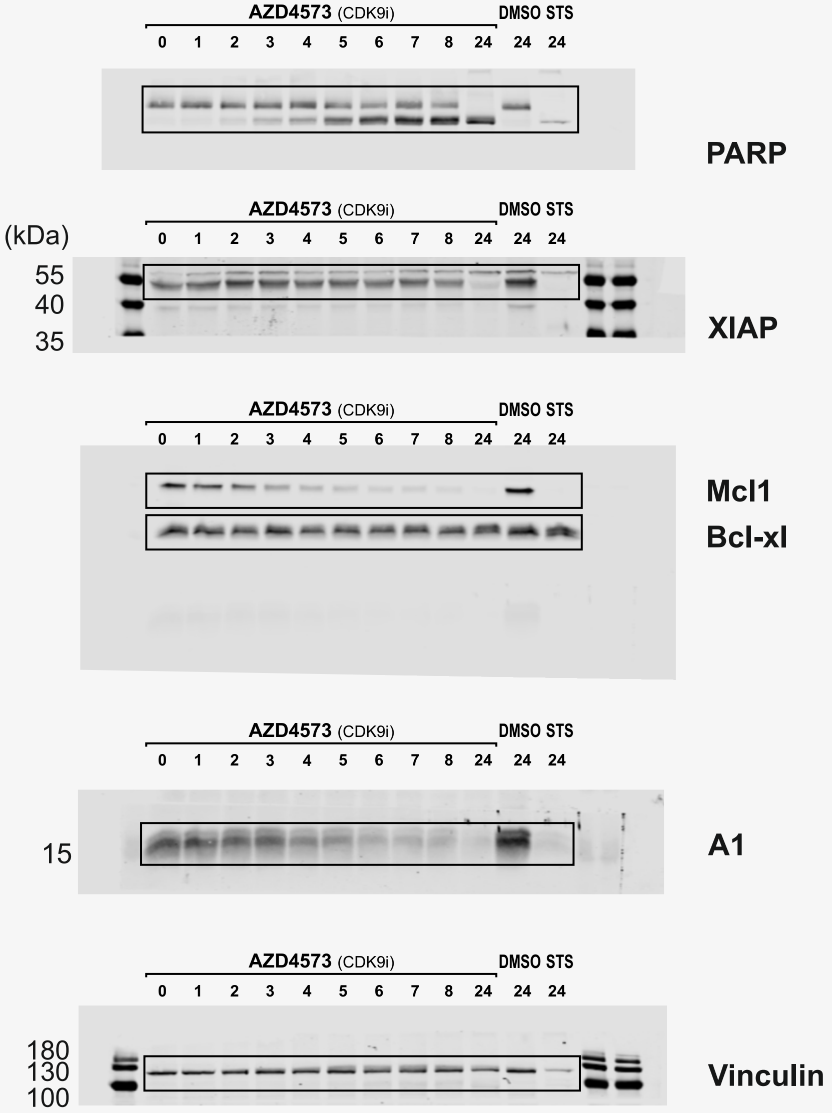

Figure 4e (right panel)

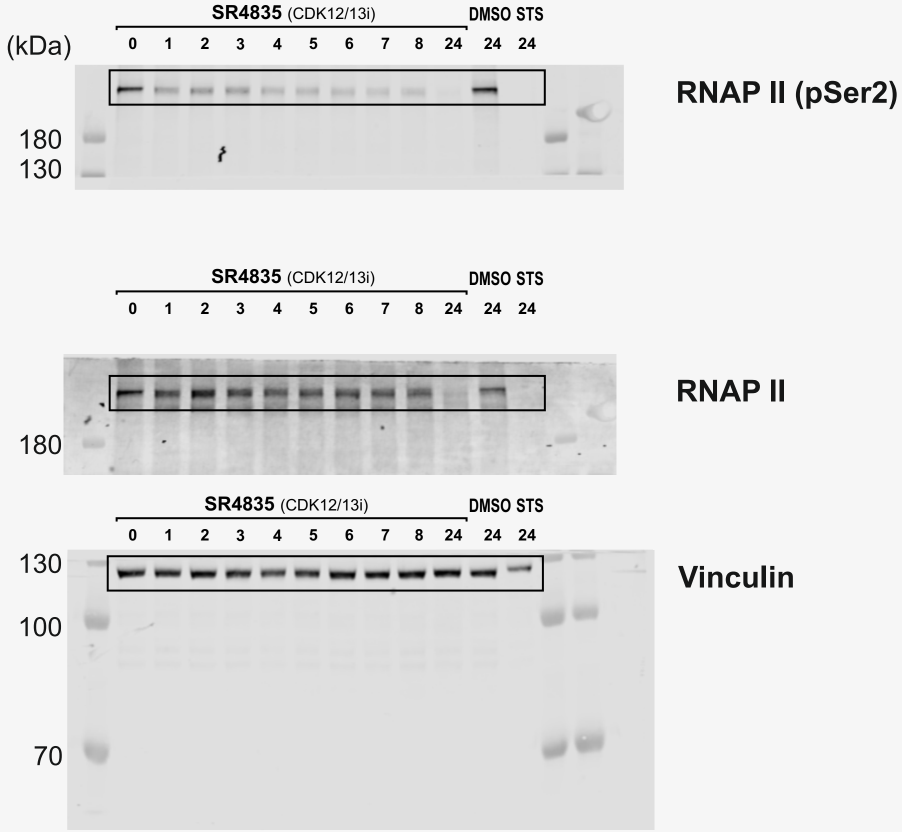

Figure 4e (right panel)

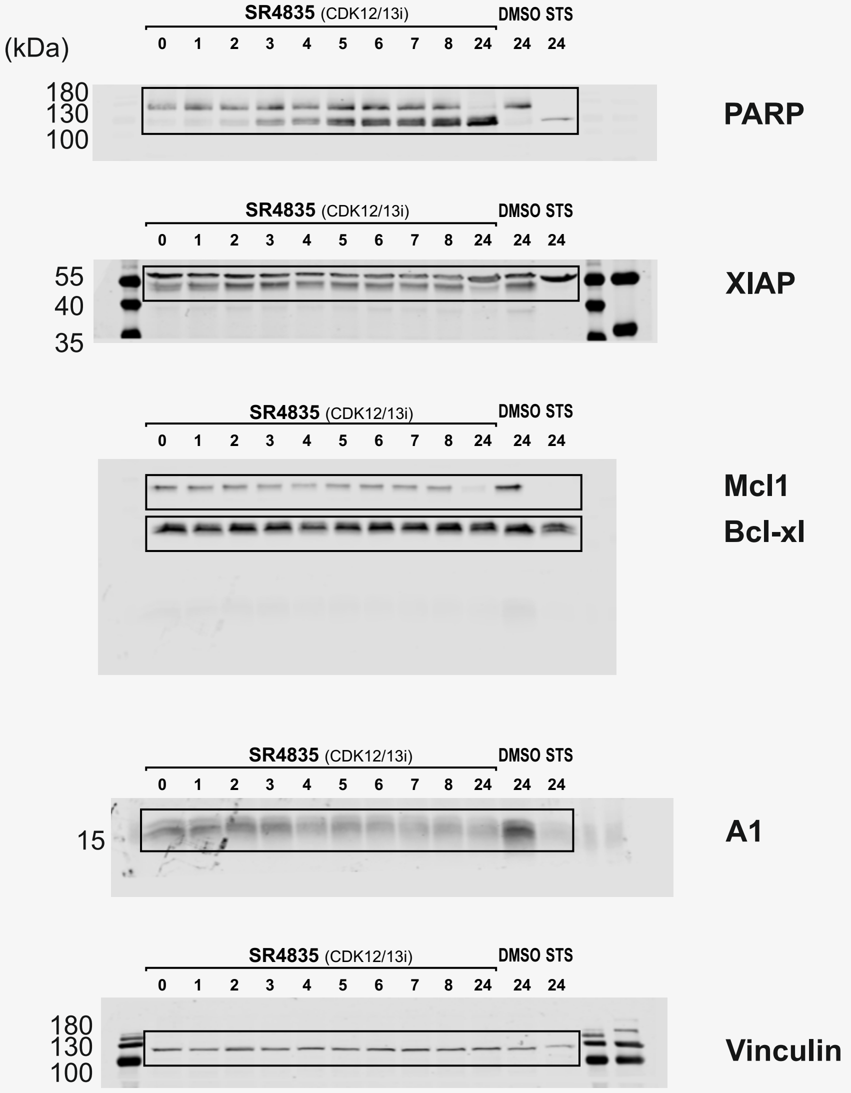

Figure 5b

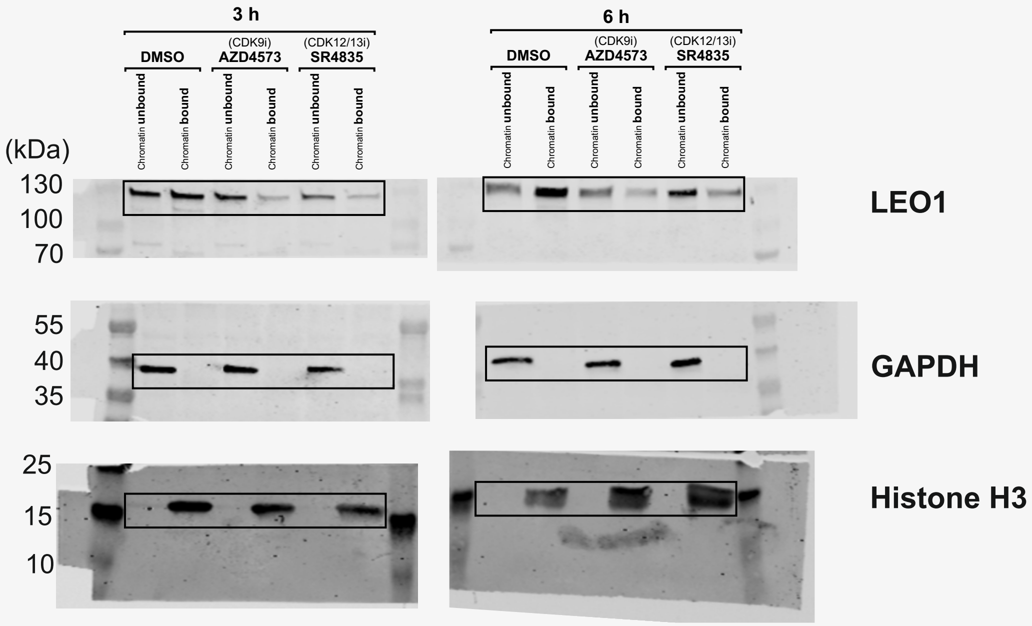

Figure 5b

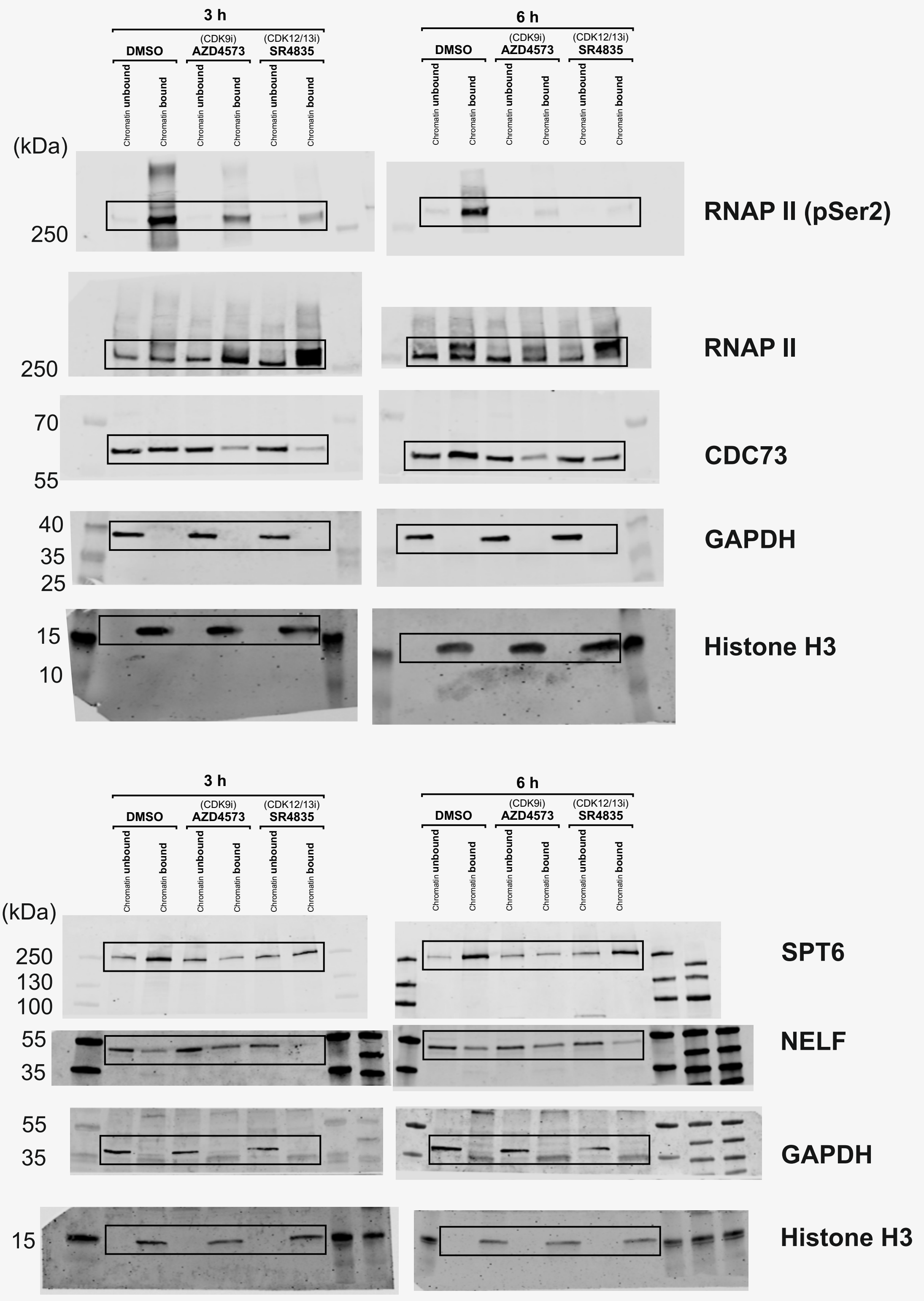

3 h
6 h
DMSO
(CDK9i)
AZD4573
(CDK12/13i)
SR4835
Chromatin unbound
Chromatin bound
(kDa)
250
70
55
40
35
25
15
10
130
100
RNAP II (pSer2)
RNAP II
CDC73
GAPDH
Histone H3
SPT6
NELF

Figure 6

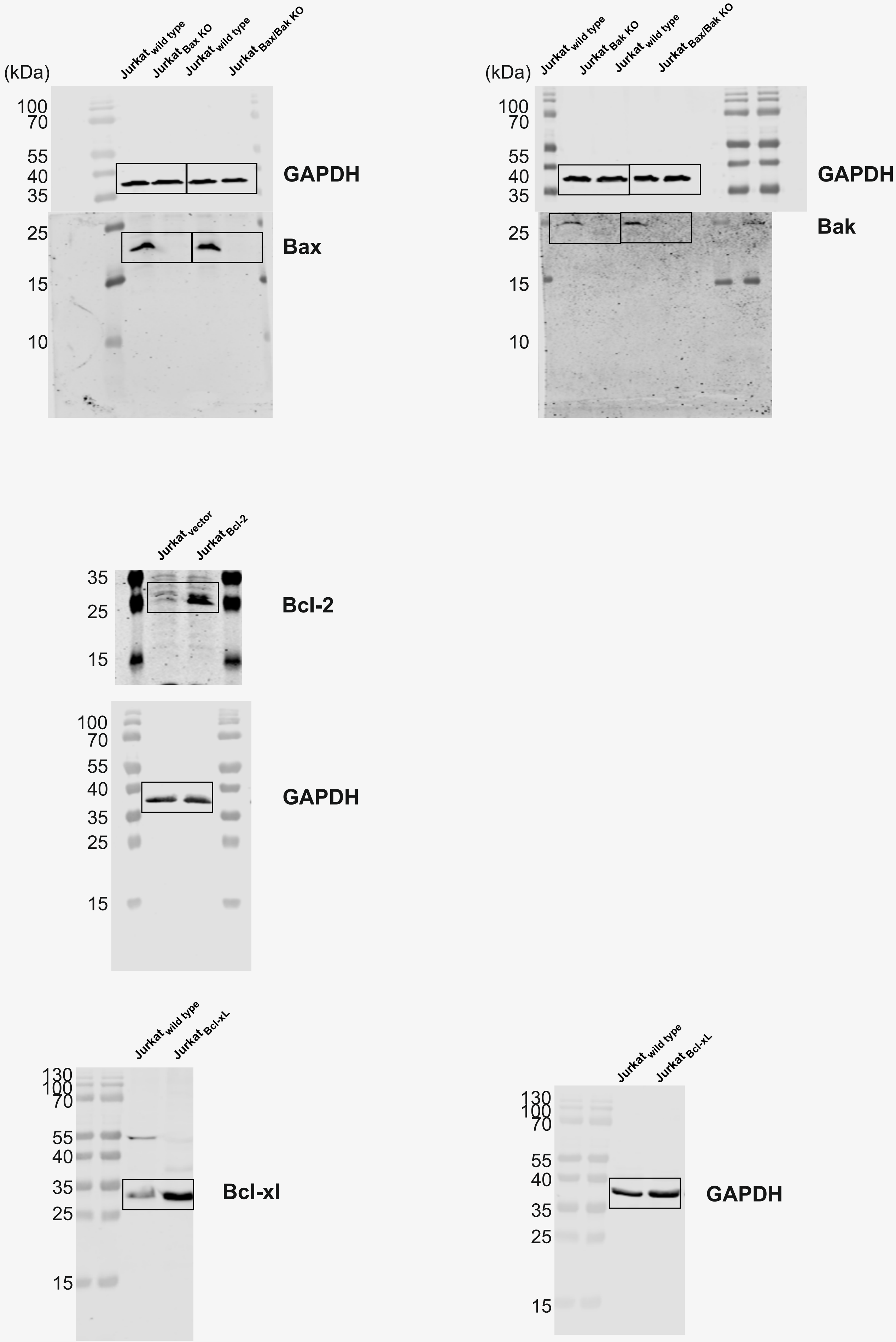

Jurkat wild type
Jurkat Bax KO
Jurkat wild type
Jurkat Bax/Bak KO
(kDa)
100
70
55
40
35
25
15
10
GAPDH
Bax
Jurkat wild type
Jurkat Bak KO
Jurkat wild type
Jurkat Bax/Bak KO
(kDa)
100
70
55
40
35
25
15
10
GAPDH
Bak
Jurkat vector
Jurkat Bcl-2
35
25
15
Bcl-2
100
70
55
40
35
25
15
GAPDH
Jurkat wild type
Jurkat Bcl-xL
130
100
70
55
40
35
25
15
Bcl-xl
Jurkat wild type
Jurkat Bcl-xL
130
100
70
55
40
35
25
15
GAPDH

Suppl. Figure S3 left Panel

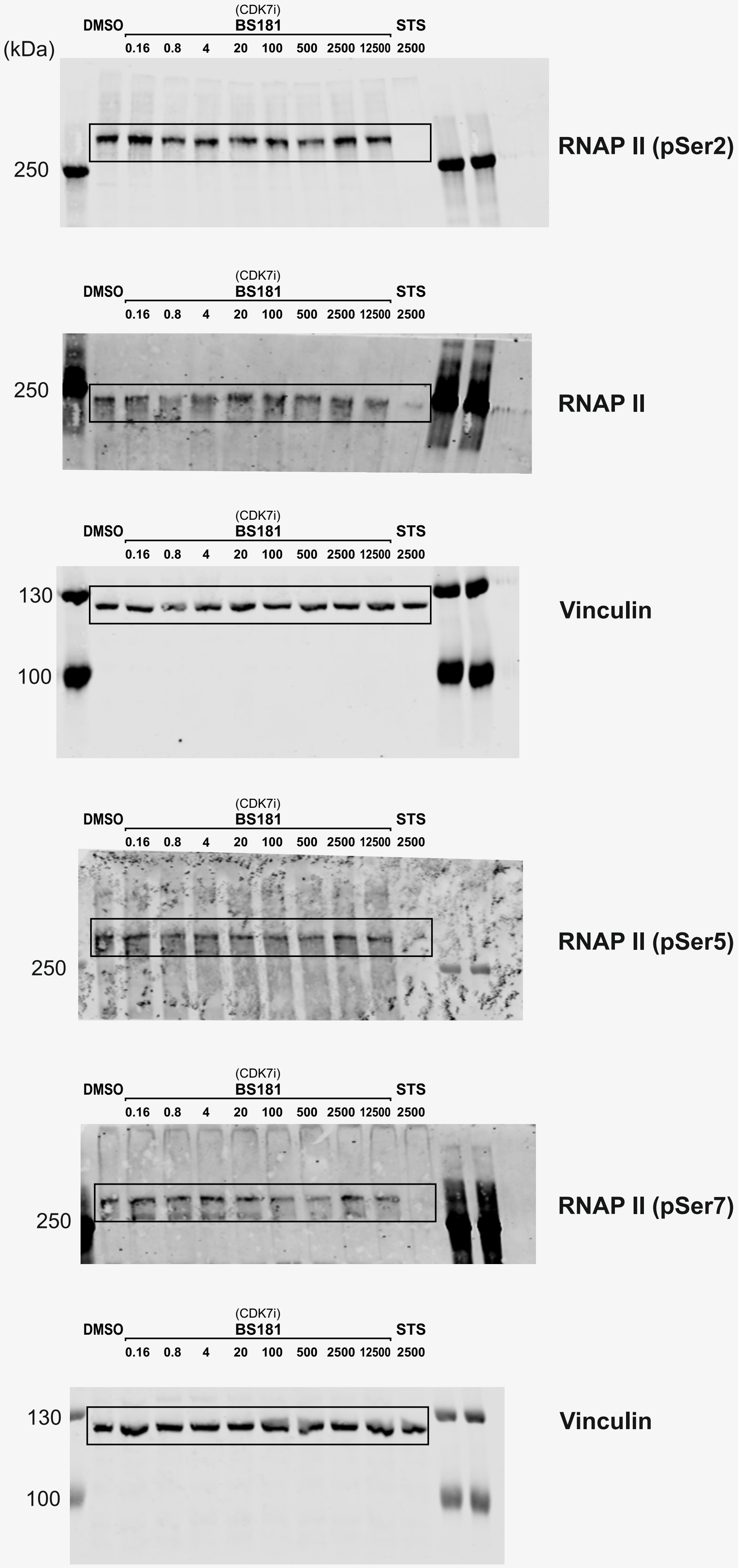

Suppl. Figure S3 left Panel

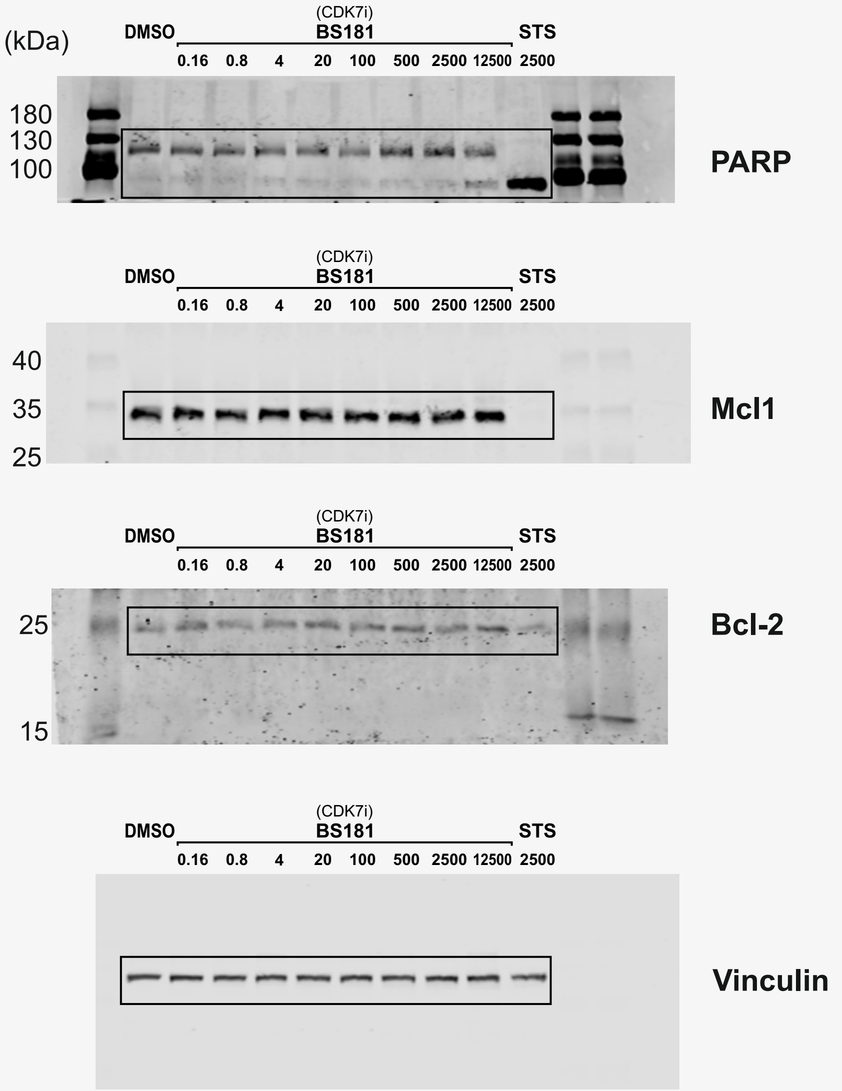

Suppl. Figure S3 middle Panel

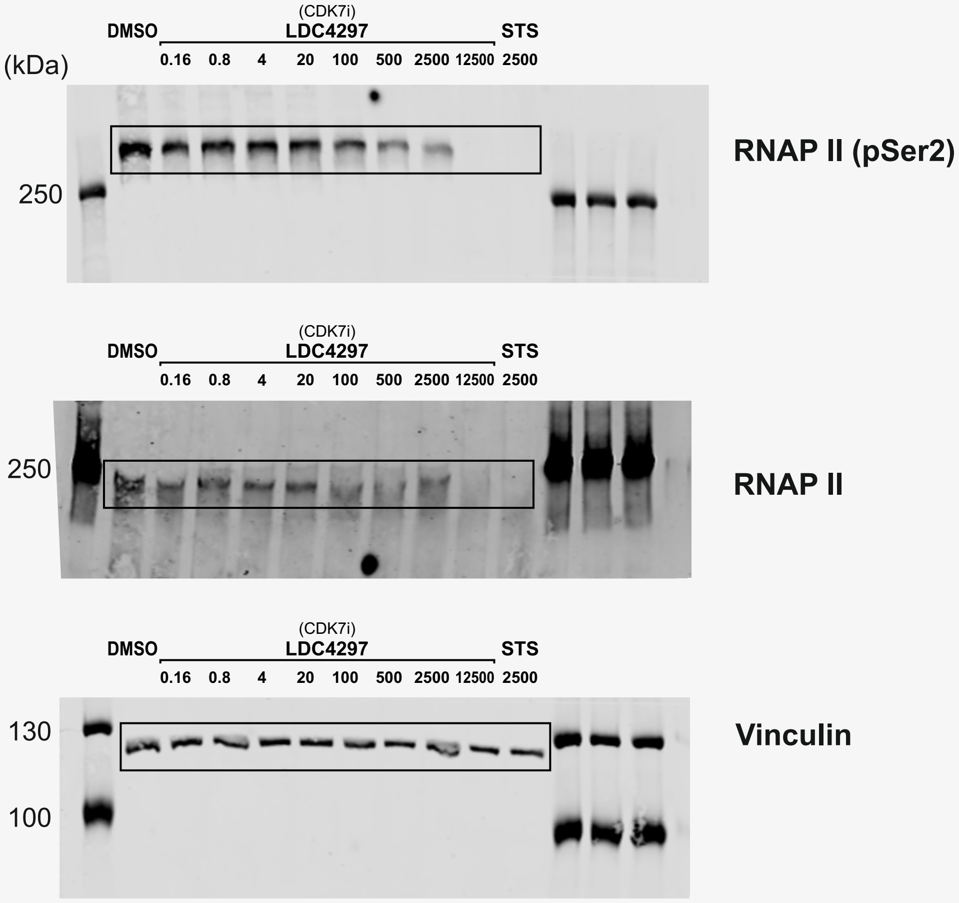

Suppl. Figure S3 middle Panel

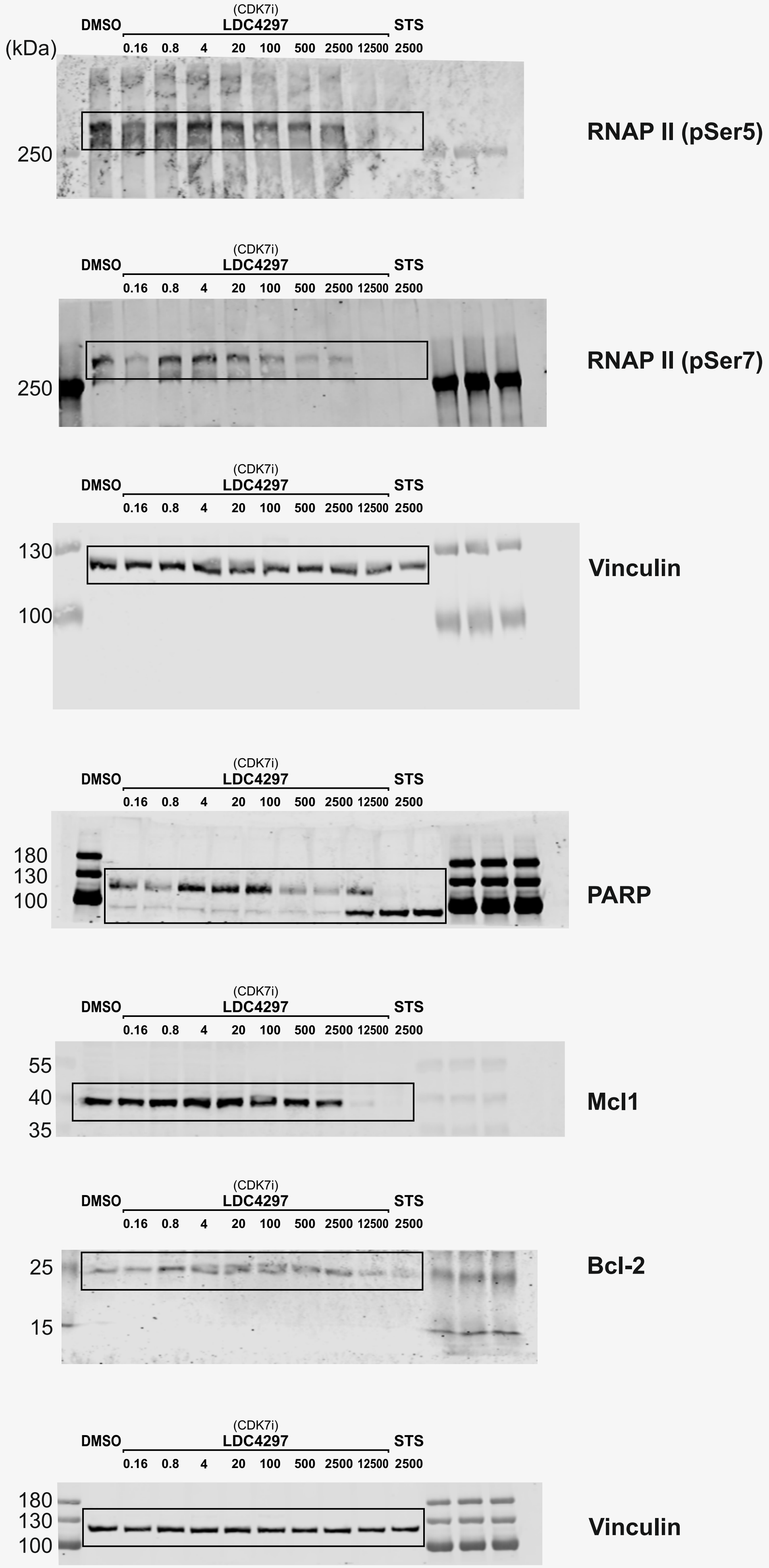

Suppl. Figure S3 right Panel

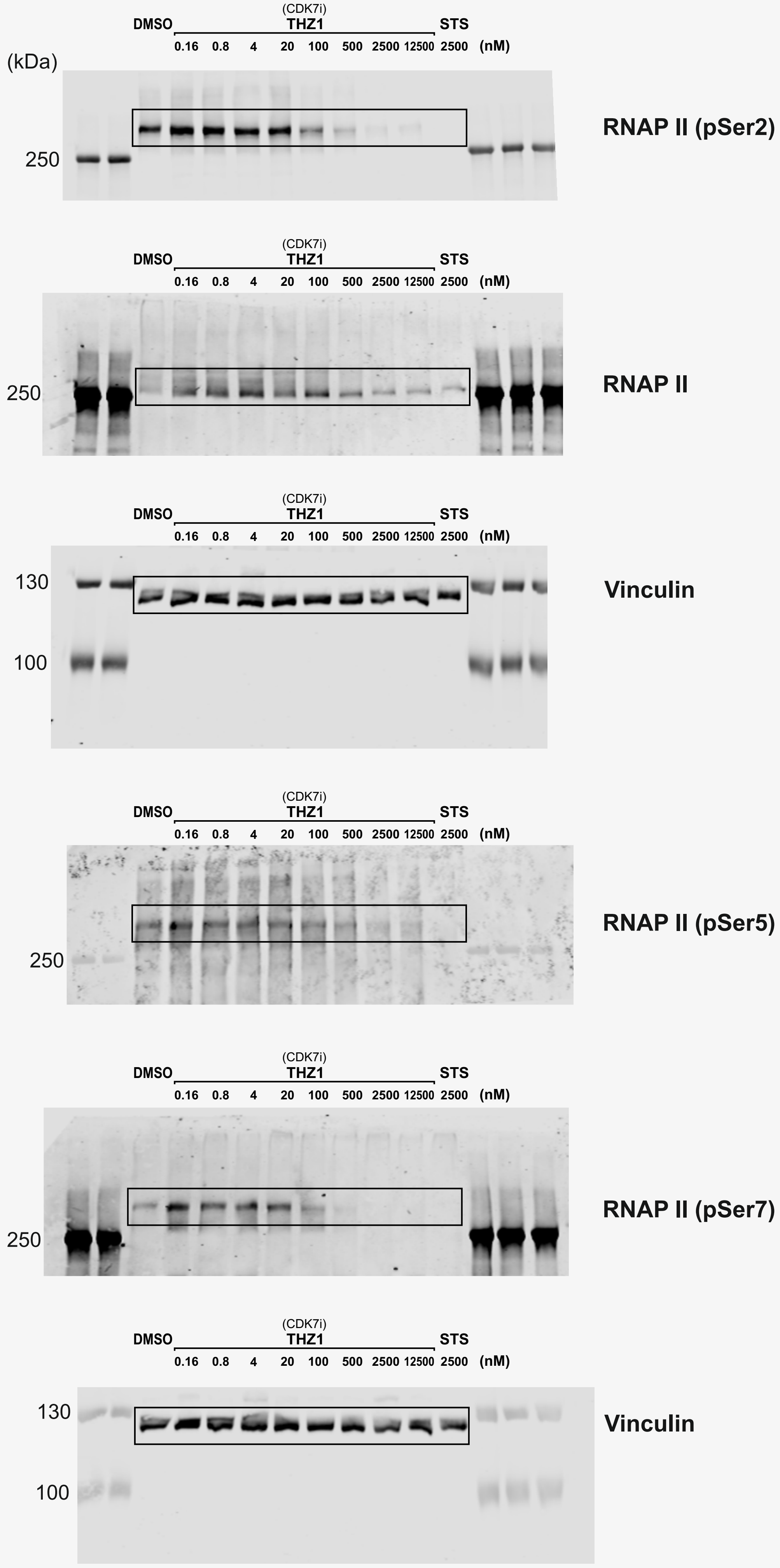

Suppl. Figure S3 right Panel

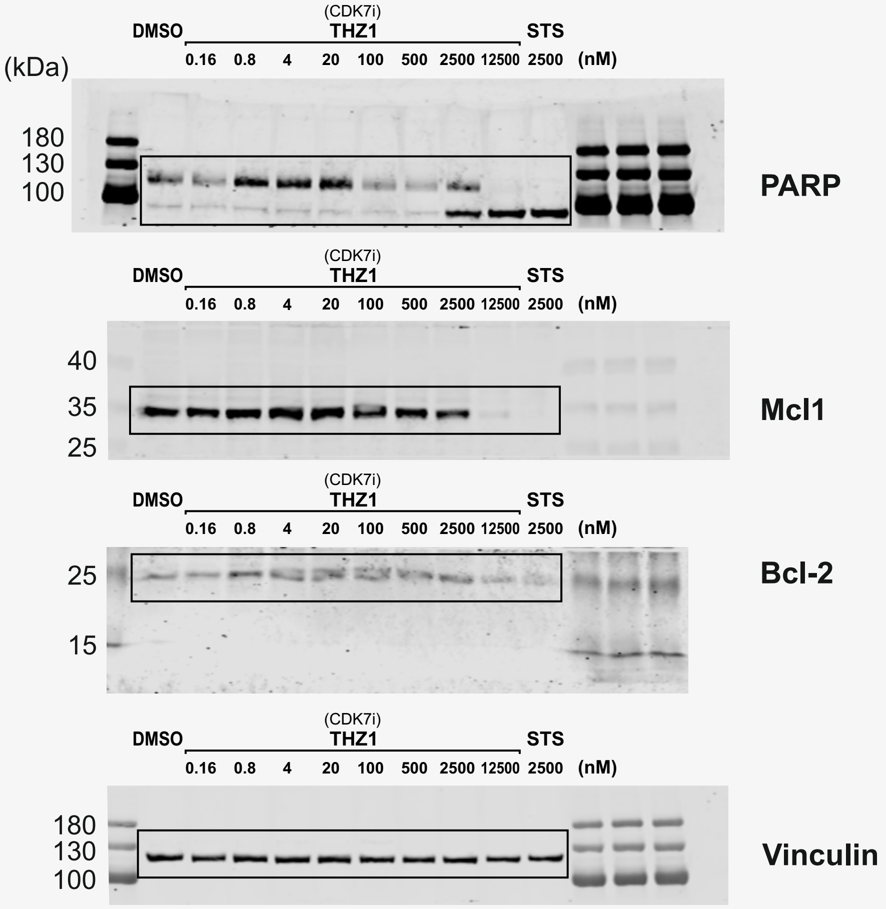

Suppl. Figure S4 left Panel

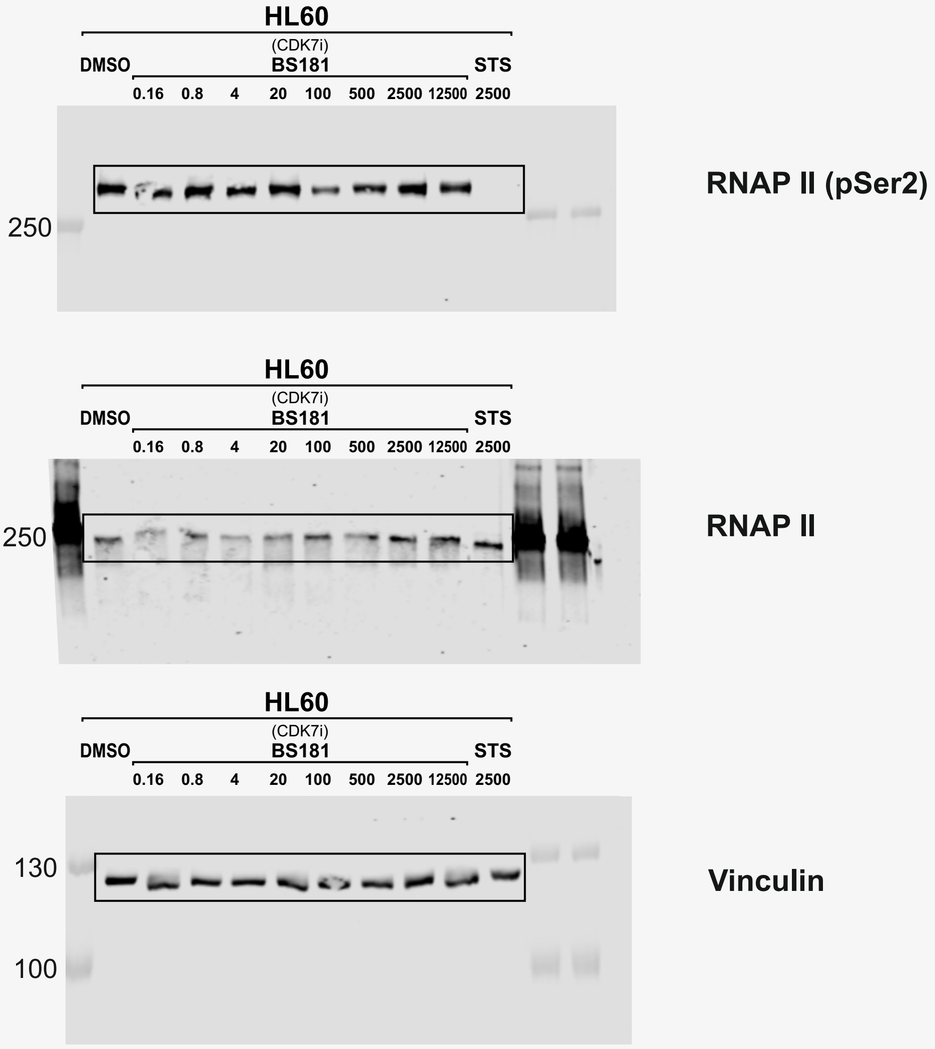

Suppl. Figure S4 left Panel

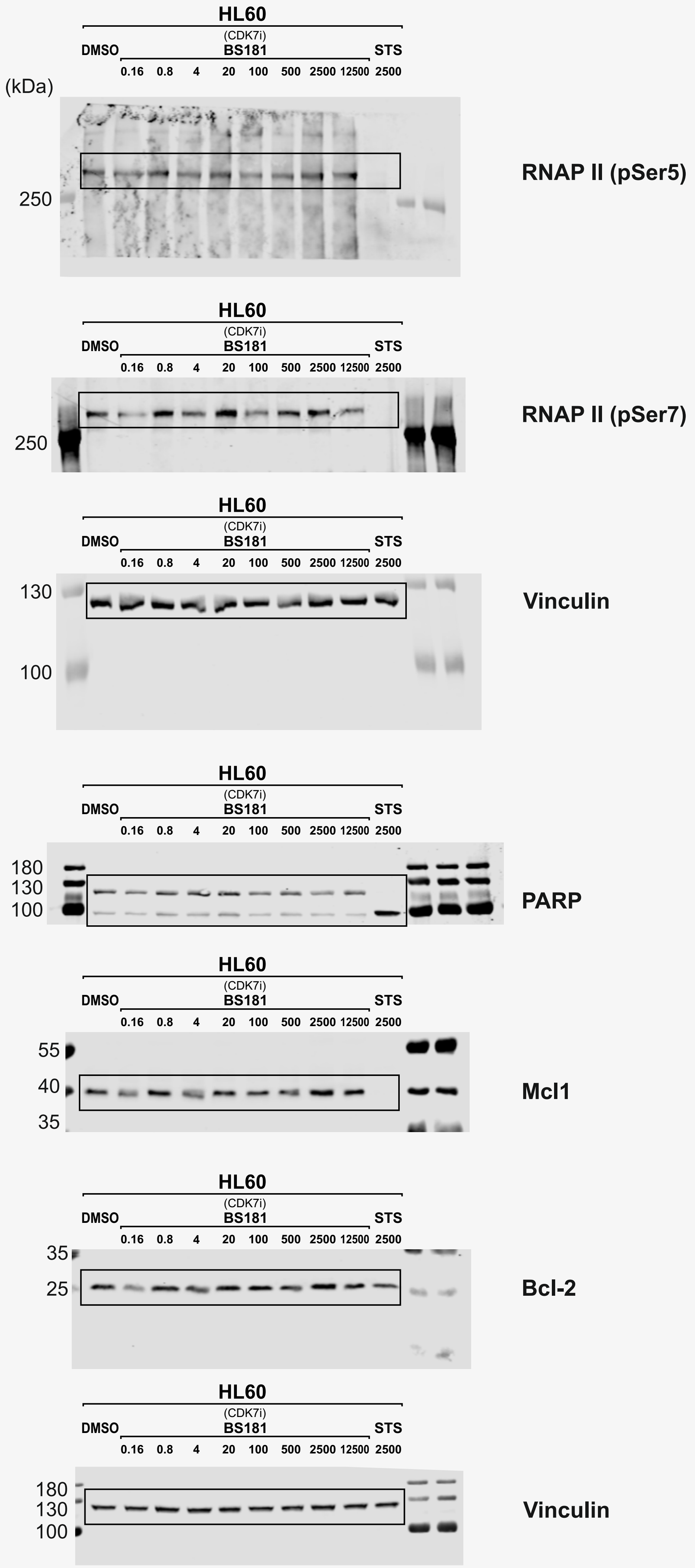

Suppl. Figure S4 middle Panel

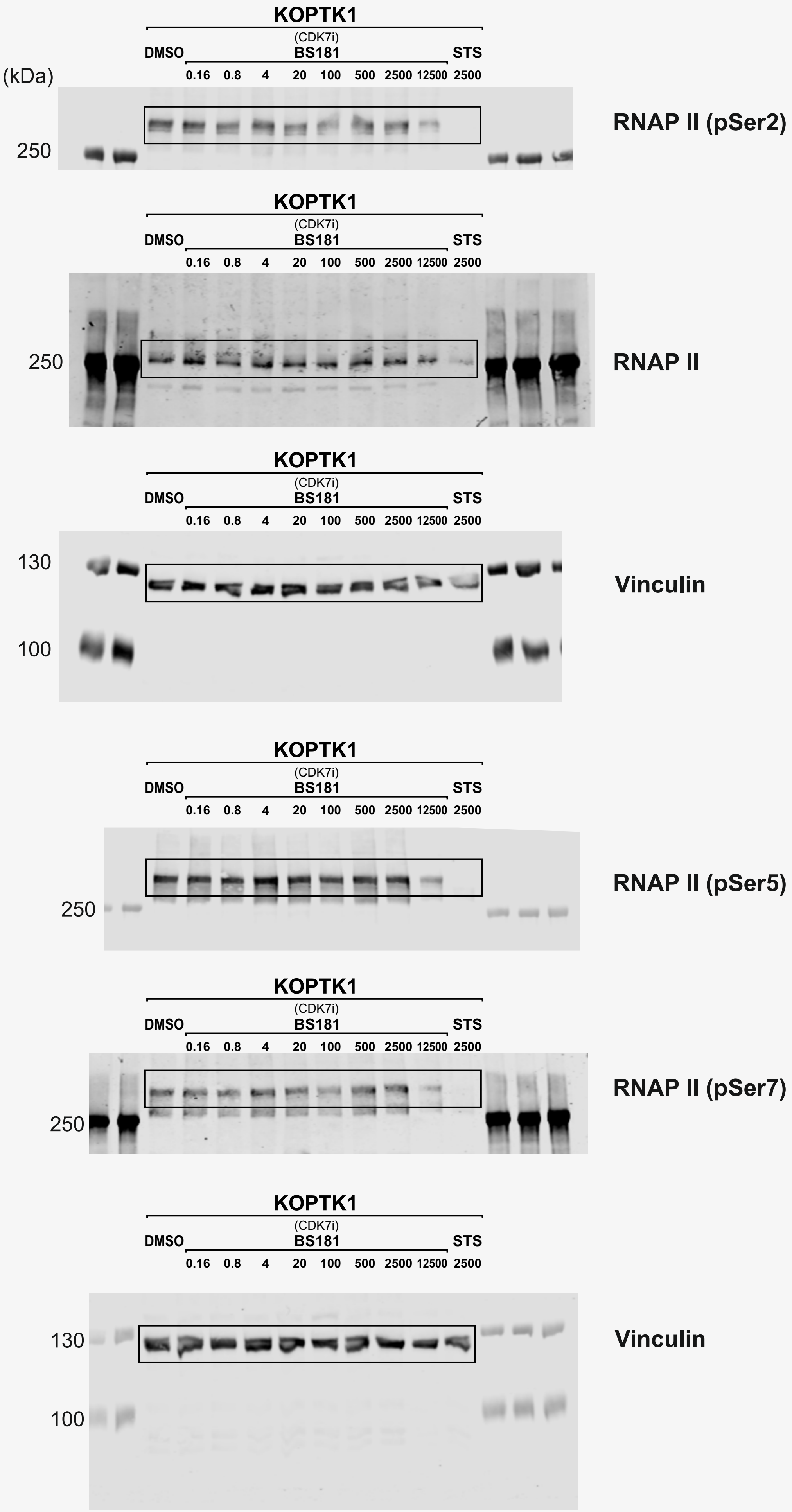

Suppl. Figure S4 middle Panel

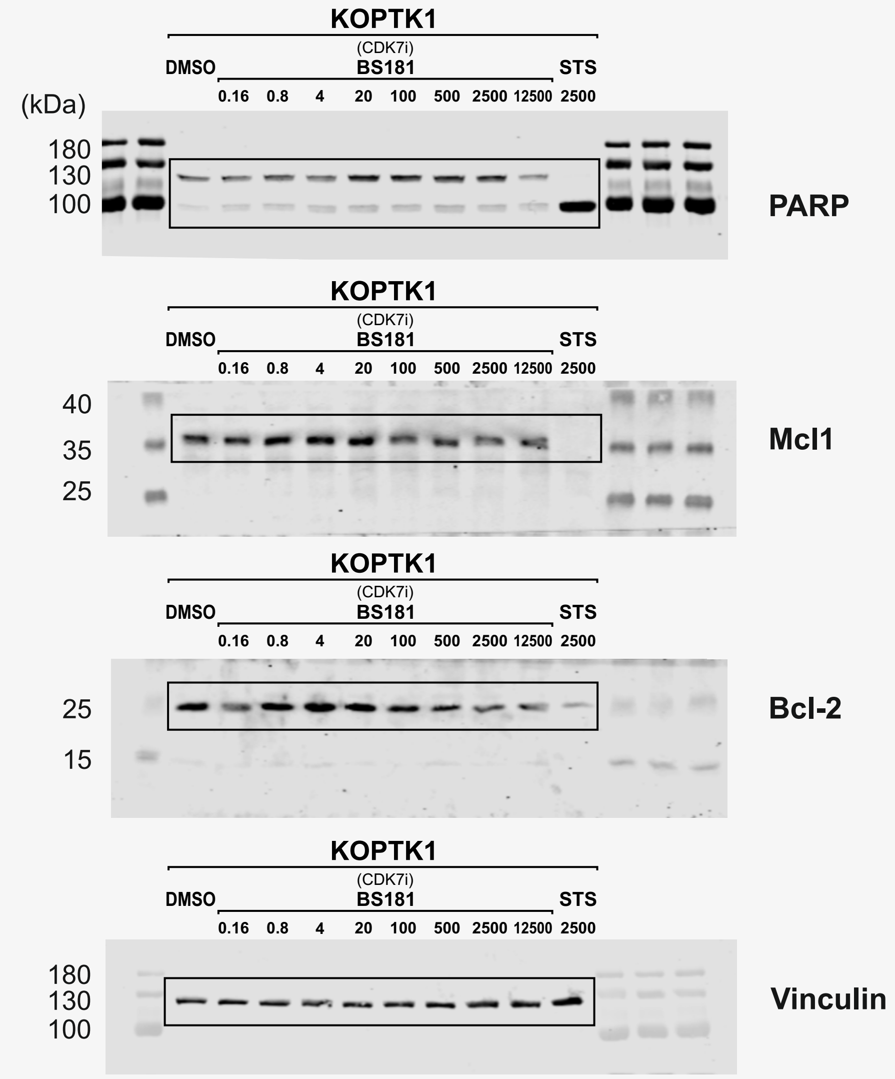

Suppl. Figure S4 right Panel

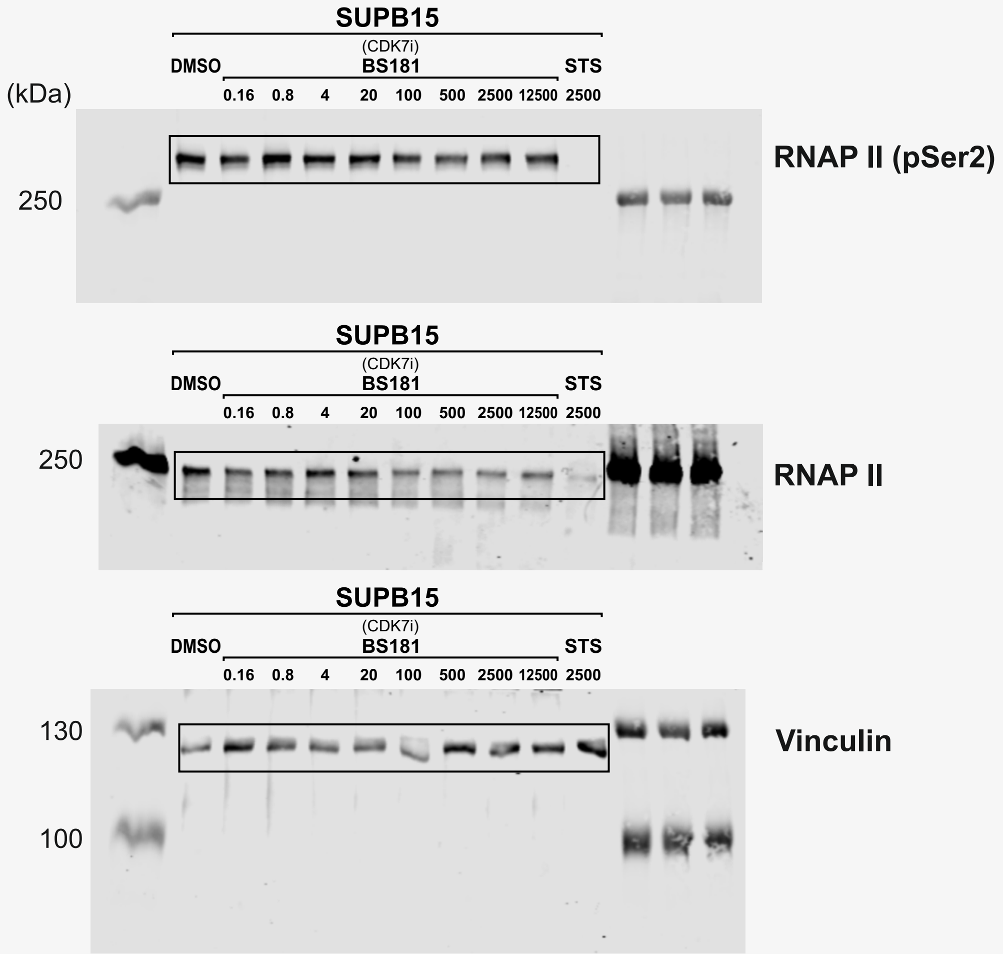

Suppl. Figure S4 right Panel

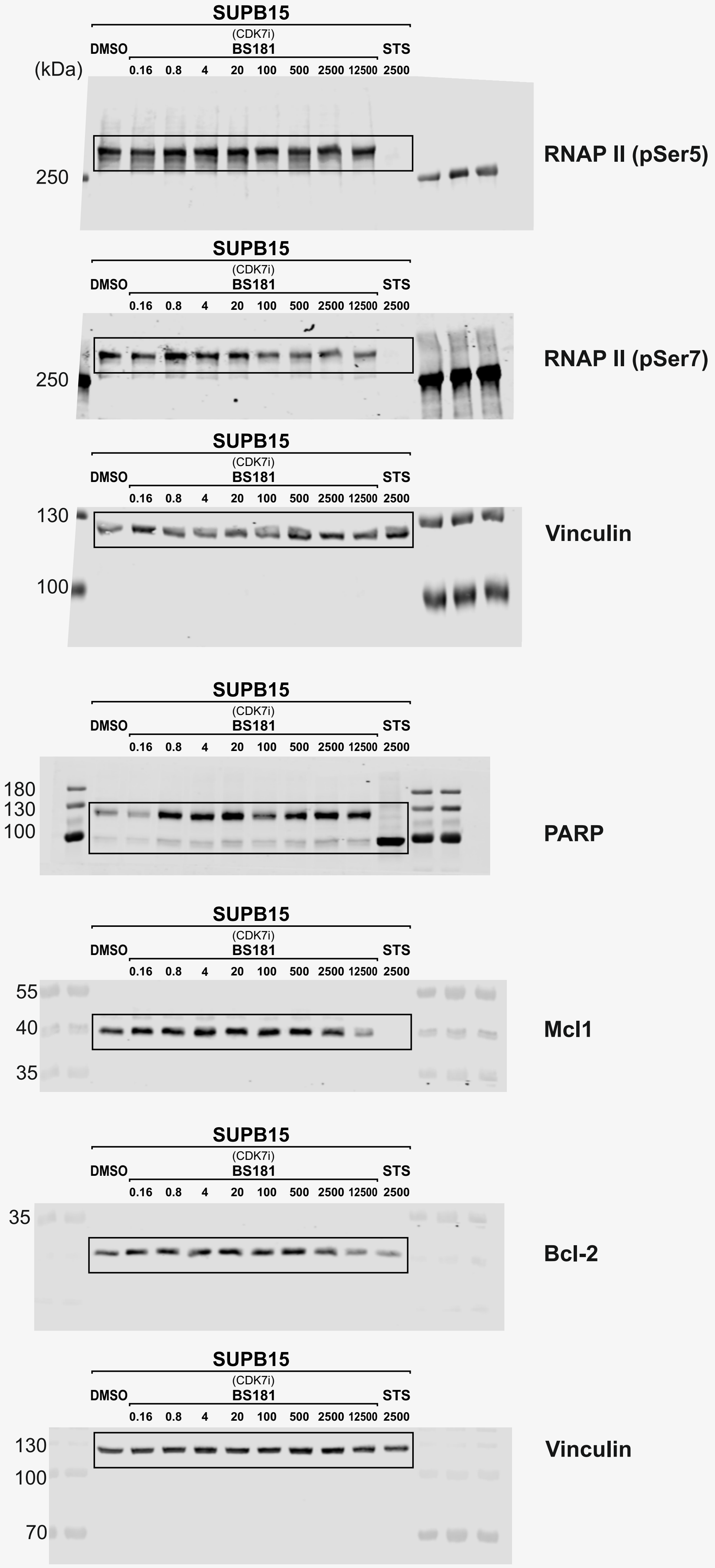

Suppl. Figure S6a left Panel

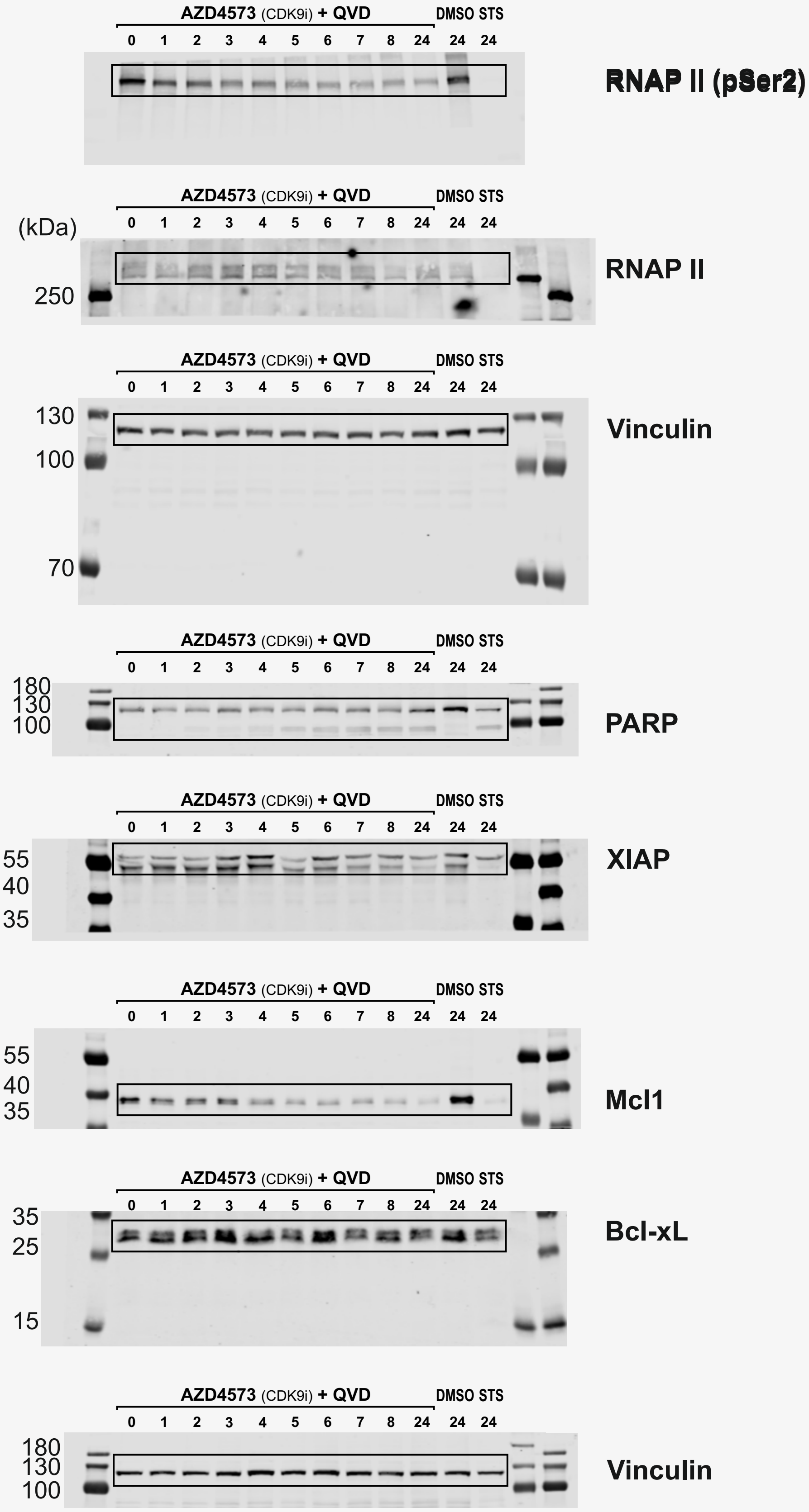

Suppl. Figure S6a right Panel

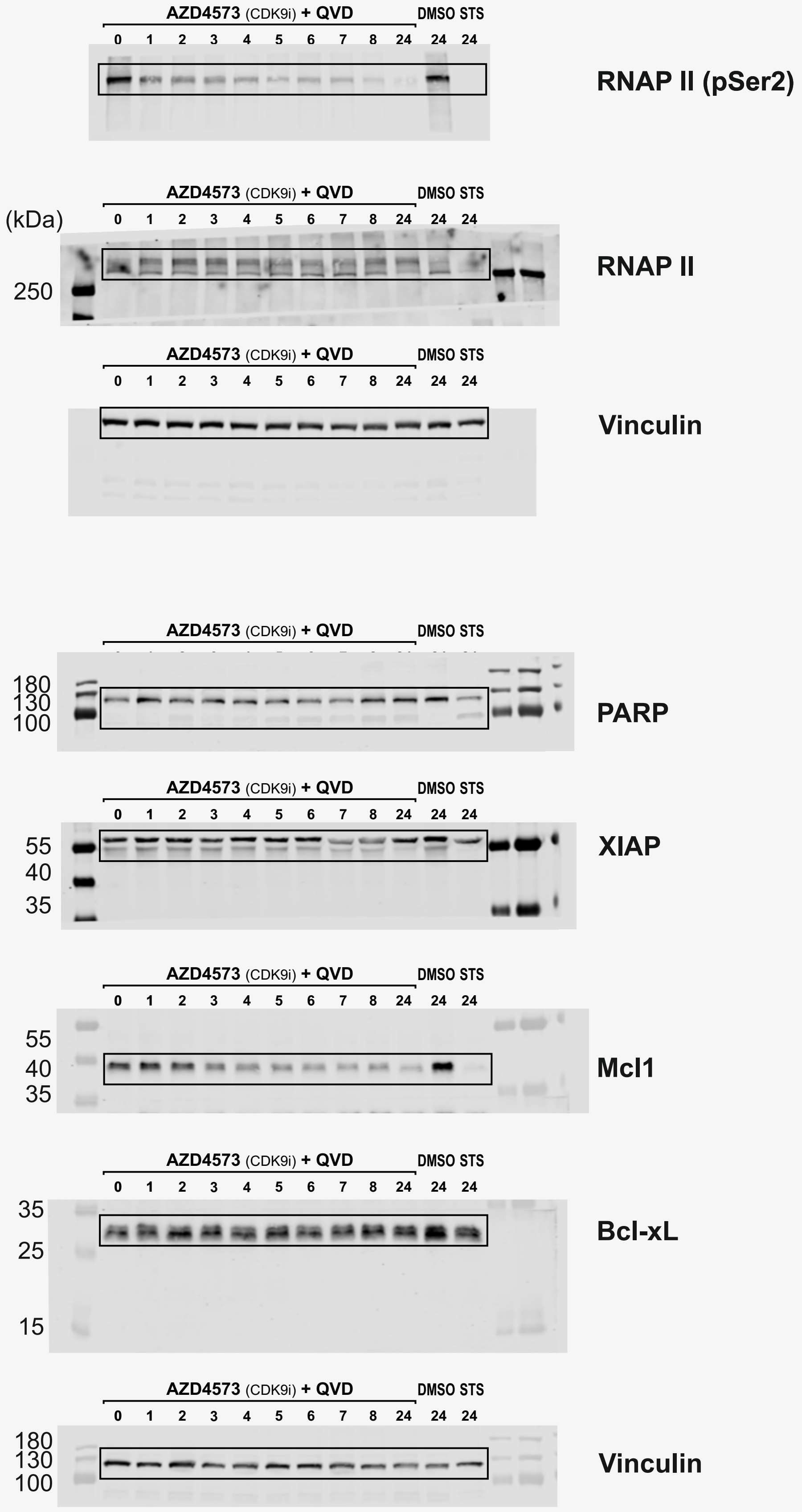

Suppl. Figure S6c left Panel

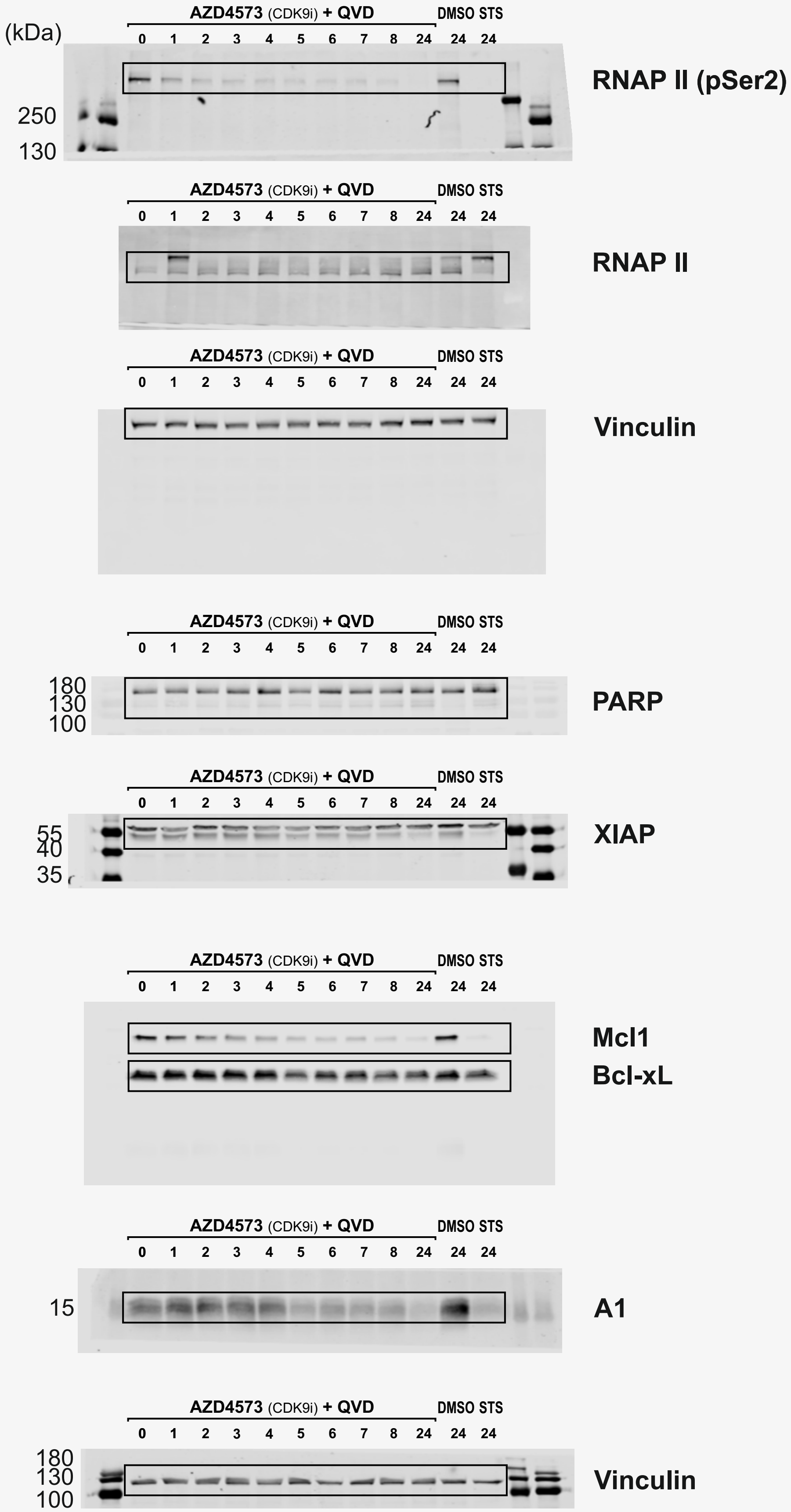

Suppl. Figure S6c right Panel

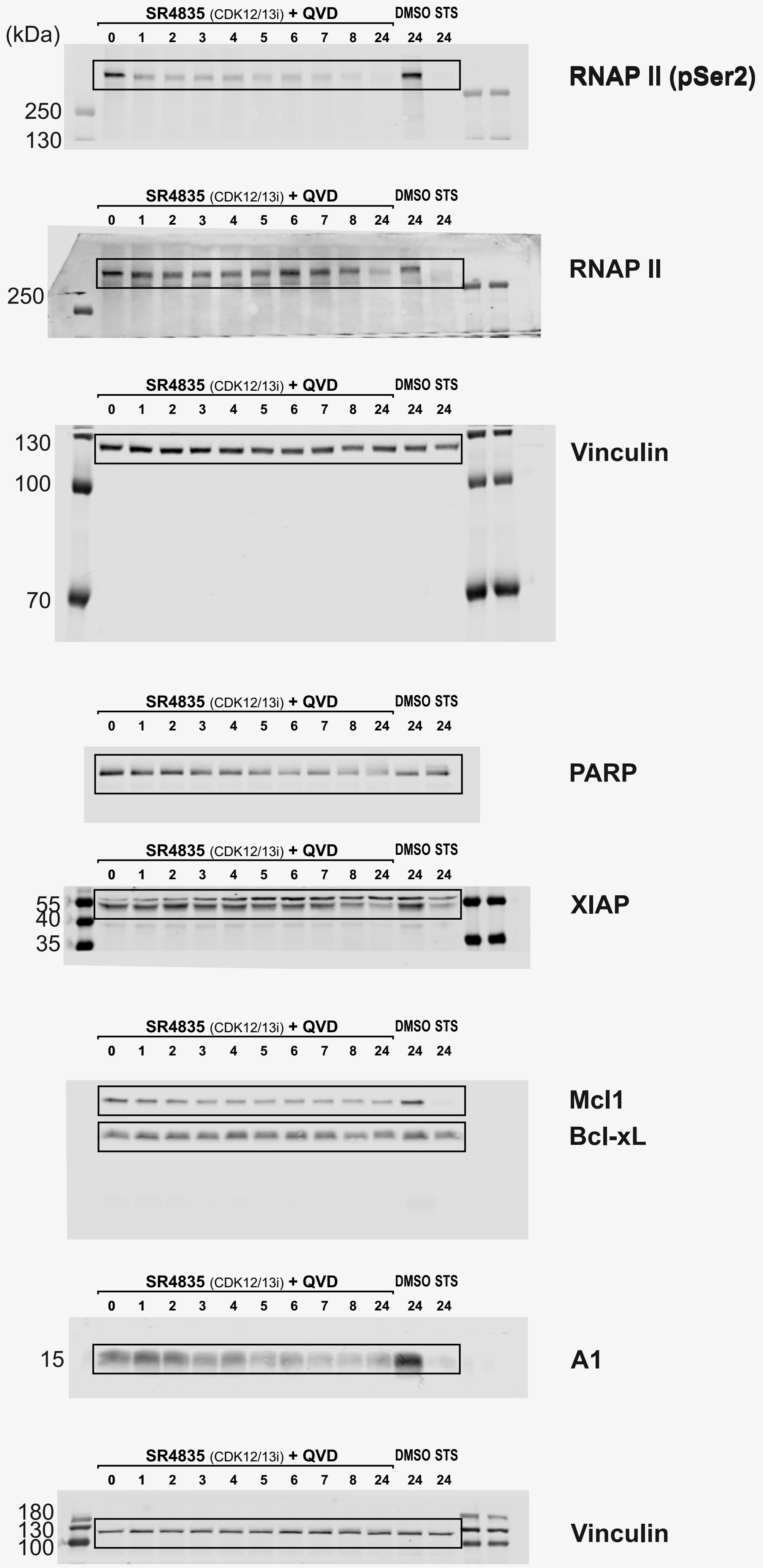

Suppl. Figure S7a

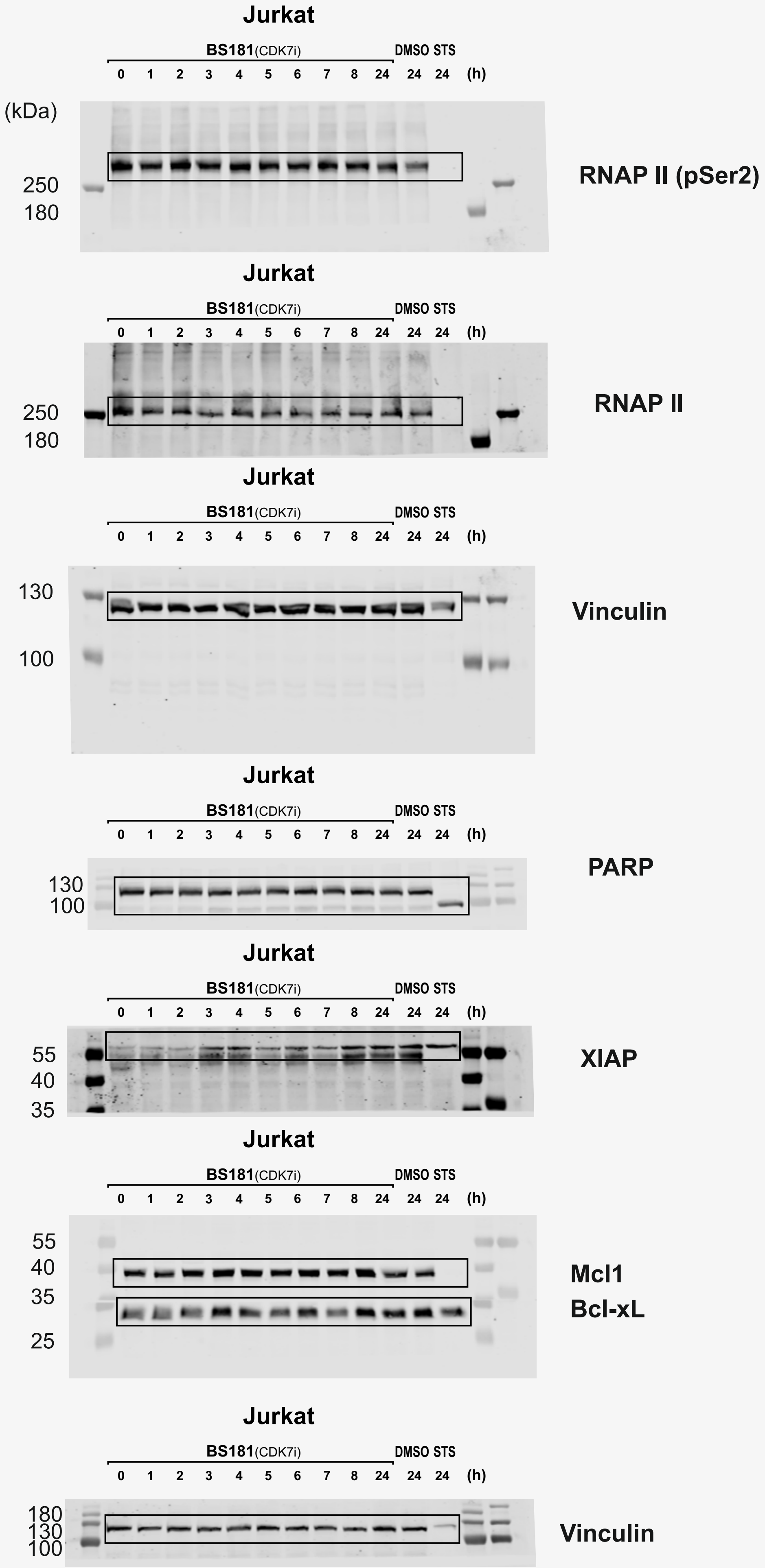

Suppl. Figure S7b

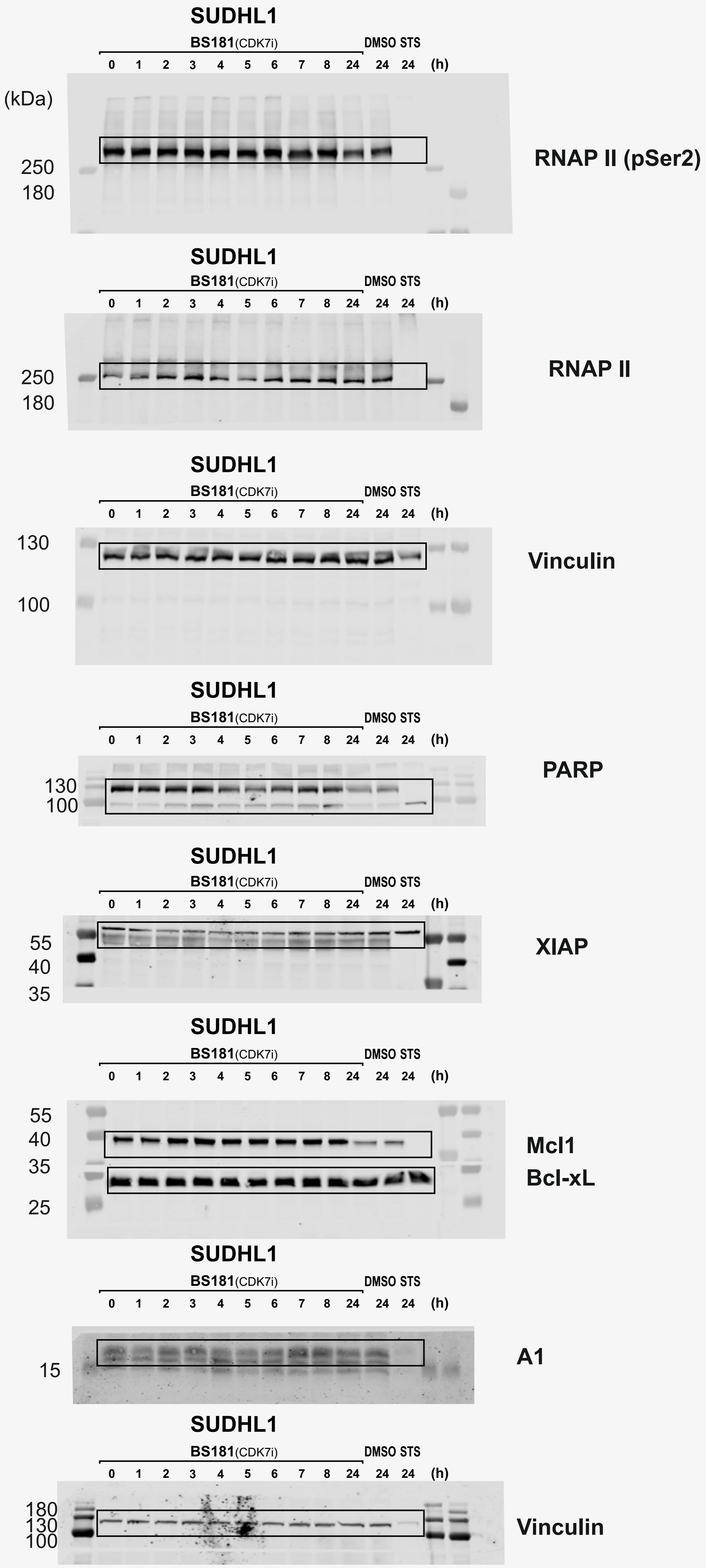

Suppl. Figure S8a

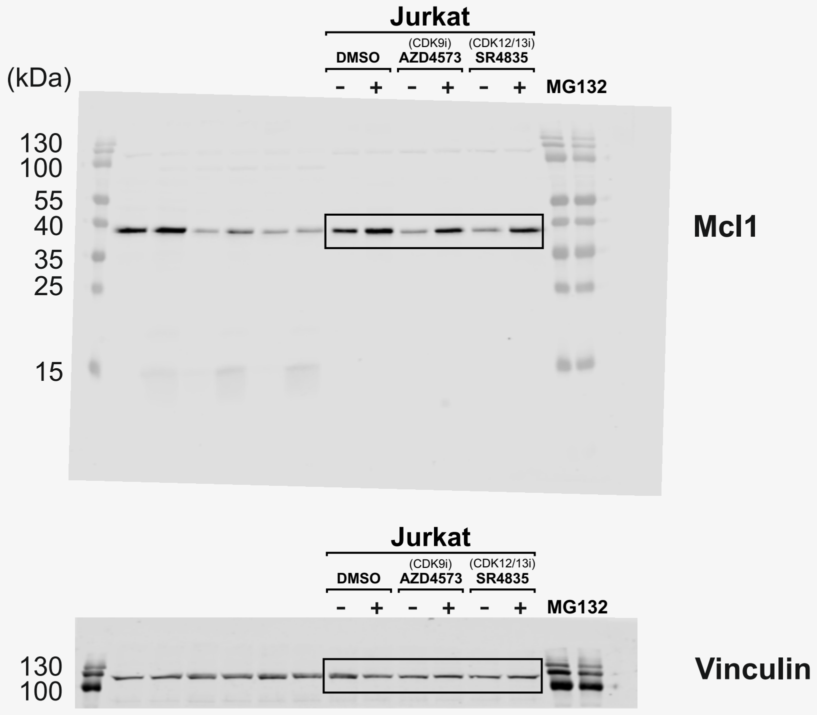

Suppl. Figure S8c

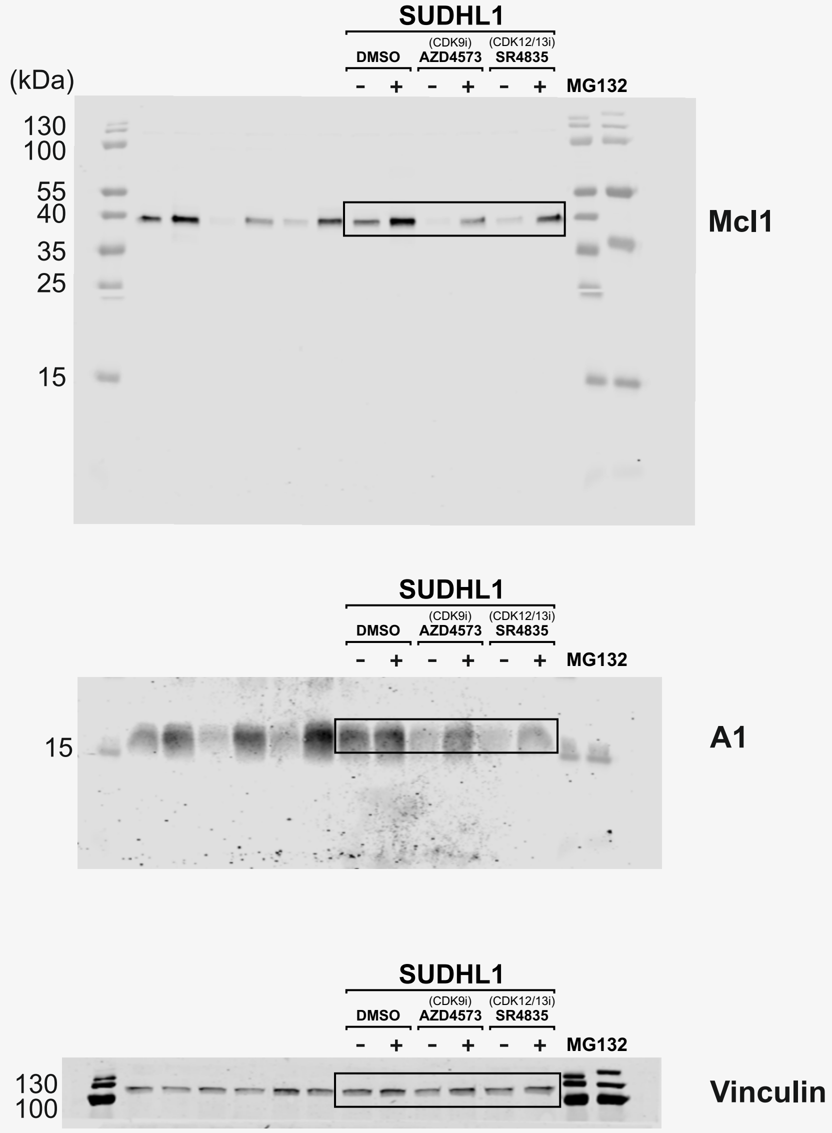

Suppl. Figure S10g

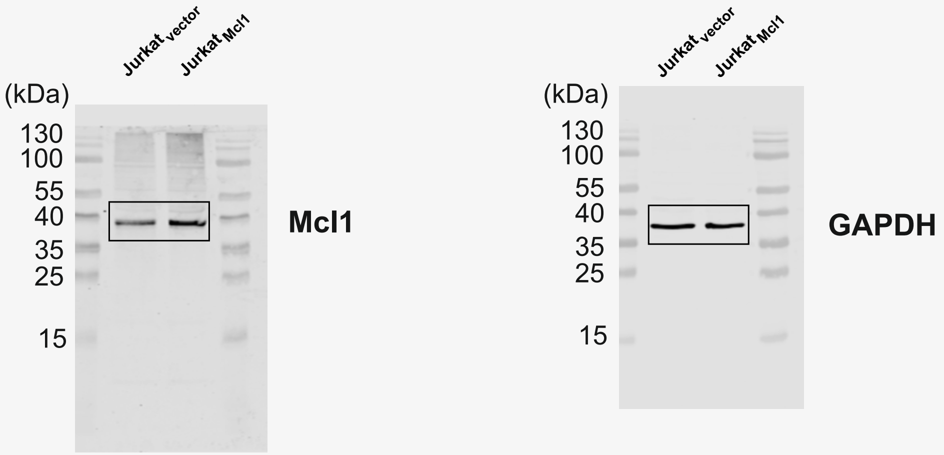

Suppl. Figure S10h

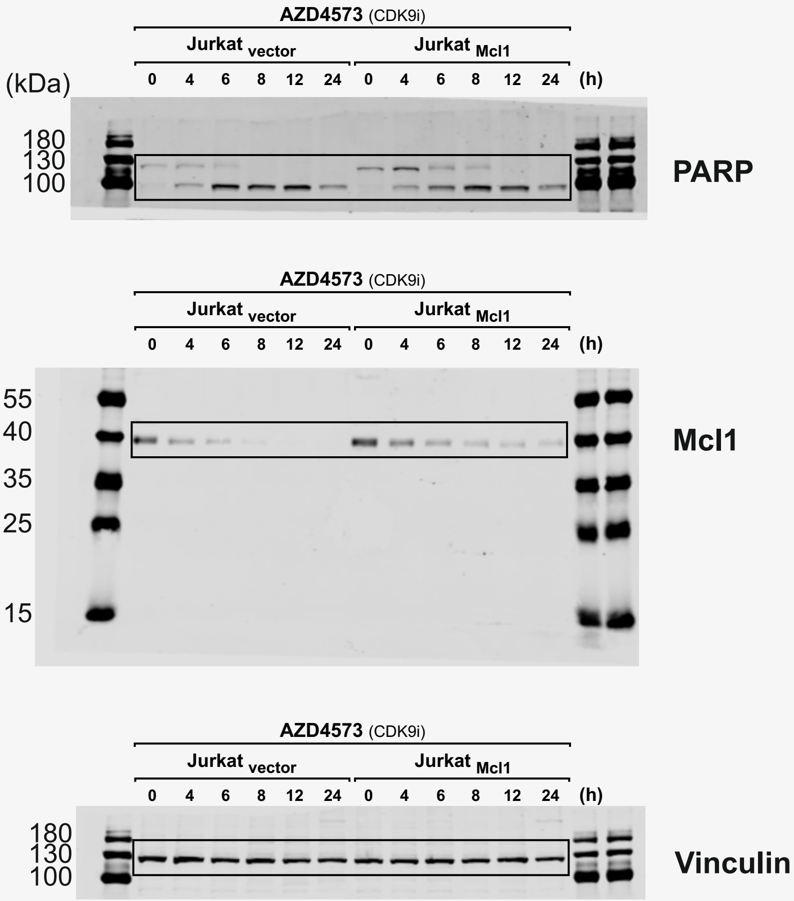

Suppl. Figure S10j

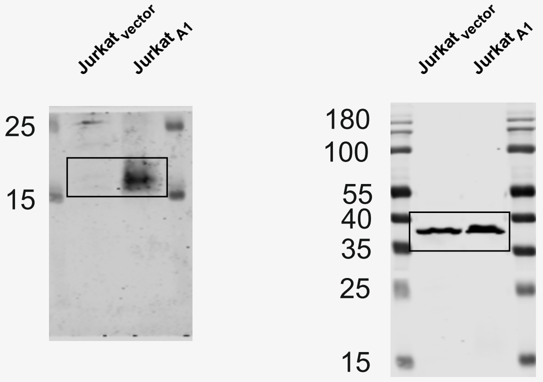

Supplement: Supplementary file 2 — Original Immunoblots [file 41419_2026_8889_MOESM2_ESM.pdf]
